# Acceptability, feasibility, appropriateness, uptake and cost perceptions of family mid-upper arm circumference supported by two-way SMS in western Kenya

**DOI:** 10.1371/journal.pone.0358775

**Published:** 2026-09-23

**Authors:** Esther M. Choo, Jonathan Lara-Arevalo, Catherine Achieng, Merceline Odhiambo, Maurine Anyango Okello, Mary Masheti, Kirkby D. Tickell, Mame M. Diakhate, Benson O. Singa, Christine J. McGrath, Arianna Rubin Means

**Affiliations:** 1 Department of Global Health, University of Washington, Seattle, Washington, United States of America; 2 Department of Nutrition, Gillings School of Global Public Health, University of North Carolina at Chapel Hill, Chapel Hill, North Carolina, United States of America; 3 Centre for Clinical Research, Kenya Medical Research Institute, Nairobi, Kenya; 4 Independent Consultant, Nairobi, Kenya; Connexi, INDIA

## Abstract

Early identification and treatment of acute malnutrition can decrease mortality and morbidity related to wasting or low weight-for-height. Teaching caregivers to measure mid-upper arm circumference (MUAC), an approach known as Family MUAC, may support early diagnosis of wasting. Family MUAC paired with mobile support from healthcare workers resulted in a 37% reduction in child wasting in the Mama Aweza trial in Kenya. This study examines the intervention’s acceptability, feasibility, appropriateness, uptake, perceptions of cost savings, and factors influencing engagement with Family MUAC. This qualitative study involved eight focus group discussions with caregivers enrolled in the Mama Aweza trial and fourteen in-depth interviews with caregivers who participated in the intervention arm, Family MUAC supported by two-way short message service (SMS). Caregivers were purposively selected based on engagement level with the intervention. Eighteen program staff and healthcare workers from three facilities in western Kenya participated in in-depth interviews. The Theoretical Domains Framework informed design of data collection tools. Caregiver and healthcare worker perceptions of Family MUAC with two-way SMS centered on seven themes. The intervention was viewed as acceptable, cost-saving, appropriate, feasible, and empowering. Caregiver uptake was high, and integrating child growth monitoring with other health services may help enhance sustainability. Barriers to uptake included inconsistent MUAC measurements, limited social support, and issues with phone sharing. Refresher trainings could increase accuracy of MUAC measurements and improve engagement. Direct communication with healthcare workers via SMS built trust and strengthened patient-provider relationships. Caregivers also reported perceived cost savings, as SMS communication reduced unnecessary travel to health centers when children were not malnourished. This study supports Family MUAC with two-way SMS as a promising approach to empower caregivers and highlights their critical role in child health and nutrition interventions. Future interventions can focus on improving measurement accuracy and mitigating negative social influences on caregiver-led interventions.

## Introduction

Nearly half of all deaths of children under five years of age are linked to undernutrition, primarily in low-and middle-income countries (LMICs) [1]. Over 45 million children are estimated to be acutely malnourished or wasted (a form of malnutrition characterized by low weight-for-height) globally, which can increase the risk of death amongst children under five by 12-fold [2]. Yet, it is estimated that only 1 in 3 children with wasting receive treatment [3]. Early identification of undernutrition and initiation of treatment can prevent wasting, and the World Health Organization (WHO) and UNICEF recommends integrating early detection and treatment of child wasting into routine primary health care services [4,5].

One promising approach to early detection and treatment is engaging with caregivers during their child’s infancy and empowering them with the knowledge and tools needed to measure their child’s mid-upper arm circumference (MUAC) at home, an approach known as Family MUAC. In some settings, home-based monitoring of a child’s MUAC by caregivers has led to earlier identification of malnutrition in comparison to active and passive screening by field workers [6,7]. Moreover, when provided with appropriate training, caregivers can perform MUAC assessments with a level of accuracy comparable to that of community healthcare workers [6]. However, there remains limited evidence on effective approaches to support caregivers in consistently performing MUAC measurements over time and in seeking appropriate care when measurements indicate childhood wasting [8].

Mobile health (mHealth) interventions, particularly two-way short message service (SMS) platforms that enable communication between caregivers and healthcare providers, offer a potentially scalable and low-cost solution to enhance health knowledge and reduce barriers to care [9]. Prior studies have demonstrated that such platforms can positively influence health behaviors, including improving exclusive breastfeeding practices and increasing timely uptake of contraceptive methods [10].

Integrating Family MUAC with a two-way SMS platform may therefore represent an effective and affordable strategy to improve early identification of childhood wasting. To test this approach, we conducted a randomized controlled trial to test the effectiveness of a caregiver-administered MUAC monitoring system with direct communication to healthcare workers via short message system (SMS). The study found that Family MUAC supported by two-way SMS was associated with a 37% reduction in wasting in children 5–12 months compared to standard of care involving community health active screening in western Kenya [8].

Effectively engaging in Family MUAC supported by an mHealth platform requires a series of caregiver and healthcare worker behaviors, including retaining knowledge on correct MUAC tape use, regularly using MUAC tapes at home, sending and responding to text message prompts to share MUAC measures, following-up at health centers when prompted to do so (e.g., in the event of a concerning MUAC measurement), and completing treatments when children are wasted. While there is considerable evidence regarding drivers of caregiver behaviors related to infant and young child feeding [11] and care seeking behaviors generally [12,13], there is minimal information about the factors that influence engagement with Family MUAC interventions. This evidence is necessary for designing programs promoting Family MUAC at scale that are culturally and contextually appropriate, and most likely to prompt targeted behaviors.

To address this evidence gap, we conducted a qualitative study to understand determinants of Family MUAC supported by two-way SMS in study sites in western Kenya. We studied caregivers whose children became acutely malnourished, whose children did not develop malnutrition, who had high engagement in the Family MUAC platform, and who had low engagement in the Family MUAC platform. The objectives of the caregiver focus group discussions (FGDs) and interviews were to assess acceptability, feasibility, appropriateness, and uptake of Family MUAC supported by two-way SMS, as well as caregivers’ perceptions of cost savings and drivers of engagement with the platform. This includes understanding drivers of high and low uptake of Family MUAC supported by two-way SMS and the comparative benefits and challenges of these tools.

## Materials and methods

### Study design

A qualitative study was conducted as part of a larger mixed methods study to investigate the determinants of engagement with Family MUAC supported by two-way SMS intervention for purposes of development and expansion. We used qualitative data to identify and compare responses between caregivers whose children did and did not become acutely malnourished during the Mama Aweza trial. Additionally, we compared caregivers with high and low engagement (high interactors and low interactors) with the SMS platform. Furthermore, we interviewed healthcare workers involved in the Mama Aweza trial to assess acceptability, feasibility, and uptake of Family MUAC supported by SMS. Study details are reported in accordance with the COREQ checklist [14] to ensure transparency (S1 Checklist).

### The Mama Aweza Trial

This analysis was conducted as part of the Mama Aweza trial, a randomized controlled trial conducted in Homa Bay and Migori Counties in Kenya [8,15]. The trial aimed to evaluate the efficacy of Family MUAC combined with a two-way SMS platform, called the Maternally Administered Malnutrition Monitoring System (MAMMS), in preventing wasting compared to standard of care (SOC) for children aged 5–12 months. The trial recruited caregivers of children visiting a maternal and child health clinic at the Migori County Referral and Teaching Hospital, St. Joseph’s Mission Hospital, and Homa Bay Teaching and Referral Hospital between August 2019 and January 2022. Caregivers of children with MUAC measurements between 12.5 and 14.0 cm at the time of recruitment were eligible to participate.

The trial compared two groups: both groups received training on how to measure their child’s MUAC. The intervention group was provided with two MUAC tapes and received weekly SMS reminders to measure and report MUAC, along with health education messages. The SOC group did not receive MUAC tapes or SMS support. The weekly health-related educational SMS messages included content based on topics including developmental milestones, recommended hygiene and sanitation practices, vaccination schedules, home and kitchen gardening, prevention of malaria, and childhood illness danger signs and were tailored to the child’s developmental stage. SMS messages also encouraged caregivers to report their child’s measurement either with the MUAC measurement color (red, yellow, green) or MUAC measurement number in centimeters. Measurements less than 11.5 cm were classified as red (severe acute malnutrition), between 11.5 cm and 12.5 cm as yellow (moderate acute malnutrition), and above 12.5 cm as green. When caregivers reported a child with a red or yellow measure, or MUAC below 12.5 cm, study staff including nutritionists and community health workers replied with a message to bring the child to the health facility for care. These trained health professionals involved in the intervention held prior training and experience in child nutrition, MUAC assessment and management of acute malnutrition. Caregiver-performed MUAC measurements identified as moderate or severely wasted were verified by a trained health professional. Messages were sent in a language chosen by caregivers (Luo, Kiswahili, Kuria or English). Caregivers received and sent messages at no cost to them. In contrast, children in the SOC group were screened for malnutrition during study follow-up visits every three months. Follow up assessments at 6 months post enrollment captured the primary outcome, time-to-diagnosis of confirmed wasting (MUAC <12.5 cm). Children diagnosed with wasting, as confirmed by a health professional at any visit in both arms, were provided treatment according to Kenya’s National Guidelines [16], with their progress monitored through scheduled follow-up visits.

### Participant sampling

After the 6 month follow up assessments had been completed, a purposive sample of caregivers were invited to participate in face-to-face summative FGDs and in-depth interviews (IDI) from Migori County Referral and Teaching Hospital between November 29 – December 3, 2021, and from Homa Bay Teaching and Referral Hospital between December 7–9, 2021. We recruited caregivers with a child identified as acutely malnourished during the study (MUAC less than 12.5 cm), and caregivers with children who did not develop acute malnutrition (MUAC greater than or equal to 12.5 cm) for participation in FGDs from each study group. We aimed to conduct eight FGDs each with 5–8 participants. Additionally, IDIs with caregivers from the intervention group were conducted to assess drivers of engagement with the platform. We purposively sampled caregivers who interacted more with the platform and those who interacted less with the platform to identify factors that influence engagement among these two groups. We defined high interactors as caregivers who responded to >90% of the automated messages with MUAC measurements within seven days, while low interactors were caregivers who responded to <30% of the automated messages with MUAC measurements within seven days [8]. An equal number of high engagement caregivers and low engagement caregivers were invited from each site. We invited participants once to participate in IDIs or FGDs.

Furthermore, IDIs were conducted with purposively sampled program staff and healthcare workers active at Migori County Referral and Teaching Hospital and St. Joseph’s Mission Hospital between June 27–29, 2022, and Homa Bay Teaching and Referral Hospital on June 30, 2022. The individual interviews assessed healthcare worker perceptions of Family MUAC supported by two-way SMS, potential barriers to sustaining or scaling the program, and opportunities to improve the program moving forward.

### Data collection

All FGDs and IDIs were conducted in the local language (Kiswahili, Luo, or Kuria), audio-recorded, transcribed, and translated into English by Merceline Odhiambo (MO), a Kenyan qualitative Research Assistant (RA). MO is a female social scientist with a bachelor’s degree in Sociology and Anthropology with over ten years of experience in collecting qualitative data. The interviewer did not have any relationship or contact with study participants prior to study commencement. Prior to study commencement, the RA received training on study procedures, qualitative data collection guides, and the application of the Theoretical Domains Framework. During enrollment of study participants, the RA shared the purpose of the study and participants were offered the opportunity to follow up with the Kenya based research team if they had feedback after the IDI or FGD concluded. All participants provided written informed consent in their preferred language (Kiswahili, Luo, Kuria, or English). All FGDs and IDIs were conducted in a private room within the hospital. The RA completed a debrief form within 24 hours of completing each FGD or IDI to provide a summary of each session.

### The Theoretical Domains Framework

We applied the Theoretical Domains Framework (TDF) to inform data collection and analysis. The TDF is an integrative framework that draws from 33 behavior change theories to characterize and evaluate behavioral determinants of implementation. The TDF comprises 14 domains with a total of 84 constructs across domains [17]. The semi-structured FGD and IDI interview guides incorporated all 14 domains from the TDF developed by the research team. The TDF also guided the development of the codebook.

### Data management and analysis

Audio recordings from the FGDs and IDIs were initially transcribed in the language in which they were conducted and then translated into English. Site investigators who did not perform the original transcriptions conducted two one-minute random spot checks per IDI or FGD by comparing the audio recordings and transcribed materials to verify transcription quality. Any discrepancies found during the audio-to-transcription comparison prompted a full review of the transcript.

We employed inductive, deductive coding and content analysis. We employed an *a priori* list of the 14 TDF-based domains, with 20 constructs embedded under the domains, as the foundation for the codebook. The codebook was structured using broader axial codes and sub-codes. Two primary coders, one based in Seattle, USA (JLA) and the other in Migori, Kenya (CA), conducted the coding with Dedoose-9.2.012. The first two transcripts were fully coded by both primary coders. When new codes emerged that were not covered in the initial codebook, the coders applied inductive thematic coding by adding new codes to the codebook. These new codes were discussed in consensus meetings, involving at least one additional researcher. When consensus could not be reached regarding coding, a third researcher (EMC) acted as a tiebreaker. During these meetings, code definitions were revised, inclusion and exclusion criteria for code application were defined, overlapping codes were combined, and codes were added or removed as necessary. After incorporating new codes from the first two transcripts, the coders proceeded by independently reading and coding all remaining transcripts.

Upon completion of the coding, differences in code application were discussed to reach a consensus on the final coding scheme. Researchers then organized coded content into thematic topics, and patterns in relevant topics were accessed to identify key themes that answered the research questions. Key themes were identified by examining concepts that were prevalent across FGDs or IDIs or highlighted areas of convergence or divergence among participants. Case memos summarized the patterns identified through coding. TDF determinants coded through the analysis were organized under key themes that emerged and visualized through a conceptual framework. The final themes were discussed among coders and the research team to ensure the conceptual framework captured the main factors influencing acceptability, feasibility, appropriateness, and uptake of Family MUAC supported by two-way SMS, as well as perceptions of cost savings. Relative comparisons were noted based on how often determinants were mentioned and compared between different caregiver strata to understand drivers of engagement. For example, we compared how often TDF domains were mentioned by high interactors and low interactors to determine barriers and facilitators to uptake of the intervention.

### Ethics

The study protocol received ethical approval from the University of Washington (STUDY00006221) and the Kenya Medical Research Institute (SERU 3821, V.1.4, 24 January 2020). An independent Data and Safety Monitoring Board composed of experts in pediatrics, nutrition program implementation, and trial methods was responsible for study oversight. All interviews were confidential, and all names or identifying information were removed from transcripts to protect the identity of the participants and their associated institutions.

## Results

We conducted eight FGDs (51 total FGD participants with group size ranging from 6–7 participants) and fourteen IDIs with female caregivers (Table 1). Among the eight FGDs, two focus groups were carried out for each of the following sets of caregivers: (1) caregivers in the intervention group whose child did not develop acute malnutrition (n = 12), (2) caregivers in the intervention group whose child became acutely malnourished (n = 13), (3) SOC participants whose child did not develop acute malnutrition (n = 13), and (4) SOC participants whose child became acutely malnourished over the duration of the study (n = 13). FGDs took between 40–104 minutes. All invited participants agreed to participate.

**Table 1:** IDI participants by interview location and title.

IDI participants	Interview location			Total interviews
	Migori County referral Hospital	Homa Bay County Hospital	St. Joseph Mission Hospital	
High SMS interactors (caregivers)	2	3	2	7
Low SMS interactors (caregivers)	2	3	2	7
				
Community health workers/counselors	3	2	2	7
Nurses	2	2	1	5
Study nutritionists	1	1		2
Ministry of Health staff/partners/nutritionists	2	1	1	4
TOTAL				32

Seven high interactors and seven low interactors participated in IDIs which lasted 24–43 minutes. Additionally, 18 healthcare workers (HCW) participated in IDIs. Overall, seven were community health workers or counselors, five were nurses, two were nutritionists engaged in the study, and four were Kenyan Ministry of Health clinical staff. HCW interviews ran 25–85 minutes.

**Fig 1:**
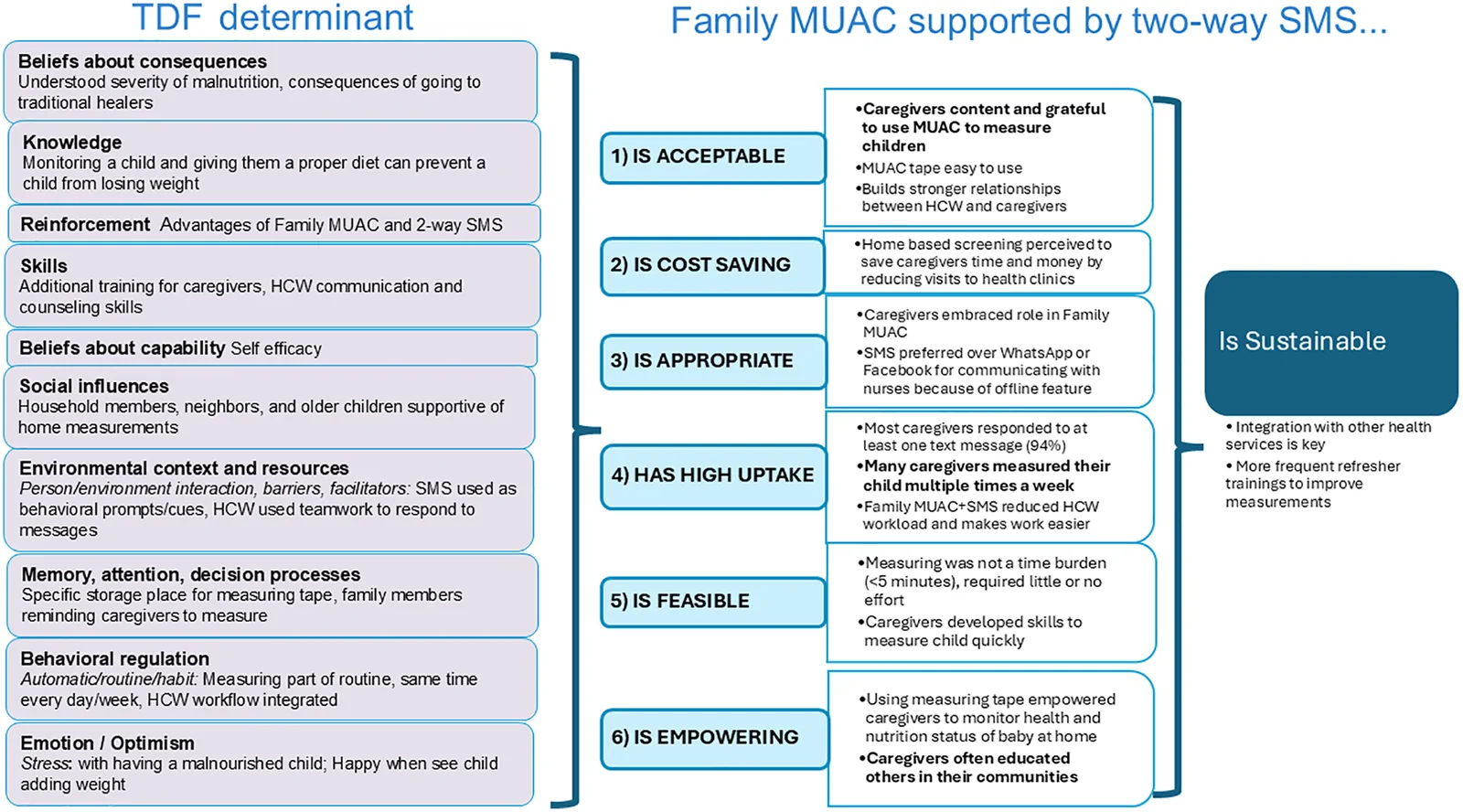
Conceptual framework of determinants driving caregiver engagement with Family MUAC supported by two-way SMS.

We identified seven themes driving caregiver and healthcare worker perceptions of Family MUAC supported by two-way SMS. These themes relate to the acceptability, perceived cost savings, appropriateness, uptake, feasibility, empowerment and sustainability of the intervention. The main themes were: 1) Family MUAC had high acceptability among caregivers and HCWs and strengthened the family-clinician relationship, 2) perceptions of costs savings contributed to increased acceptability of Family MUAC, 3) Family MUAC supported by two-way SMS is an appropriate mechanism to connect with caregivers and to relay health messages, 4) uptake of the Family MUAC is generally high, but can be compromised by individual and social-level barriers, 5) integrating Family MUAC supported by two-way SMS behaviors into routine practice made participation more feasible, 6) Family MUAC empowered caregivers to track their child’s nutritional status and became health advocates in their communities, and 7) HCWs reported that integrating Family MUAC into other health services and daily routine would increase sustainability (Fig 1).

The most influential TDF domains supporting these themes included beliefs about consequences, knowledge, reinforcement, skills, beliefs about capability, social influences, environment context and resources, memory attention and decision processes, behavioral regulation, and emotion (Fig 1). The determinants grouped under each theme are described in more detail below.

### 1) Family MUAC had high acceptability among caregivers and HCWs and strengthened the family-clinician relationship

Overall, Family MUAC supported by two-way SMS was perceived as acceptable. Of the caregivers randomized to the intervention group, 528 caregivers out of 532 felt very comfortable or somewhat comfortable using the MUAC tape [8]. Five primary TDF determinants drove the acceptability of the intervention: *beliefs about consequences*, *environmental context and resources*, *reinforcement*, *beliefs about capability*, and *emotion/optimism*. Most caregivers were content to measure their child’s MUAC, and considered the procedure with the MUAC tape easy to perform (TDF domain: *beliefs about capability*). No considerable difference was noted between high/low interactors and caregivers with acutely malnourished or malnourished children. Caregivers and children bonded over the activity of measuring MUAC, with children enjoying getting measured over time (Table 2).

**Table 2:** Additional supportive quotes for acceptability of Family MUAC with 2-way SMS intervention.

TDF domain (construct)	Additional supportive quotes
Beliefs about capability	“I can say it was easy because you didn’t have to use force to measure. You just sit down relaxed and the child also liked it when he sees it he runs and comes for the measurement, so we really enjoyed the process.” – FGD #2
Environmental context and resources (person x environment interaction)	Nurses are ‘human and jovial..and make you feel comfortable to share with them your child’s businesses” – FGD #2 “They could pass here and say ‘I’ve been sending you SMS, who has been answering that SMS’ [I: It made you guys become friends?] yes so that communication was a great bond between a caregiver and us. Also, those whose mark had fallen to yellow and red they could even share it here with us ‘that if it were not for you this one would have even died’ [I: So, they are giving you guys’ positive feedback and appreciation?] Yeah, they appreciated it and it brought us closer to one another, yes.” – Counselor ID #3

In addition, connecting directly with HCWs on a regular basis resulted in stronger relationships between HCWs and caregivers (TDF domain: *environmental context and resources*) through building trust and improved communication, resulting in greater satisfaction with the patient/provider relationship which is a key to delivery of quality health services. One counselor mentioned, confidence “in using MAMMS system to engage with the mothers because the system has made the interaction easier, and it has brought us together with the caregivers so that they share their feelings with us, and we also share our experience with them.” (Counselor IDI#2)

Caregivers also found HCWs to be friendly, polite, supportive, and trustworthy in their interactions (Table 2). One respondent remarked “with the nurse who was sending the SMS, we were assisting each other to benefit the health of my child, it was like she has joined me to monitor, she was like a support system for my child.” (FGD #2)

Caregivers understood the gravity of having a child identify as malnourished and the negative consequences of consulting traditional healers instead of trained HCWs (TDF domain: *beliefs about consequences*). Caregivers became motivated when they saw children put on weight (TDF domain: *emotion*) and it encouraged them to continue to engage with HCWs via SMS and measure MUAC: “It was easy, when I saw my child adding weight I was so motivated and happy, I was measuring and sending the message”(FGD #1).

The educational content delivered via SMS in the intervention group was perceived by caregivers to be useful and important to keep a child healthy, namely the importance of hygiene during breastfeeding and practicing personal hygiene to prevent child illness. Receiving these informational messages reinforced the utility of participating in this study and encouraged caregivers to read their messages (TDF domain: *reinforcement*). One high interactor mentioned:

“they would even send me some educative messages, whether I am feeding the baby well, how is the baby’s health, is my baby sleeping under a bed net. Sometimes when I was spreading the net I would check if it was torn or something. So I found the messages I was being sent very beneficial to me.. I was becoming knowledgeable with information that I didn’t have.” (High interactor IDI#2)

### 2) Perceptions about cost savings increased acceptability of Family MUAC

Caregivers perceived family MUAC supported by two-way SMS to be cost saving, primarily driven by the TDF determinant of *knowledge*. Connecting with a healthcare worker via free SMS messaging to discuss the health and growth of their child reduced the need to visit a health clinic. This may have reduced costs associated with transportation, food, and time spent bringing a child to the health facility, especially when a child was not malnourished. An FGD participant remarked:

“[Participating in Mama Aweza] relieved us of the burden of transport money, coming to town every day, every day, every day because we were taking measurement every week…at least in that regard they have really relieved us of the transport burden, we are very grateful.” (FGD #1)

Both HCWs and caregivers mentioned the benefits of reducing transportation costs through engaging in telehealth consultations via SMS. A counselor mentioned, “one advantage was that it was cost effective for the caregiver, because the caregiver was not coming and going back most of the time for the measurement. They were only supposed to come when the child was identified.” (Counselor IDI#1)

High interactors mentioned that they engaged more freely with the HCWs via SMS as it was at no cost to them. On the other hand, some low interactors erroneously believed that messages sent or received were at a cost to caregivers and found messages difficult to understand, which were barriers to uptake of the intervention (TDF domain: *knowledge*). One low interactor mentioned, “I just knew that [the messages] were not free because I have never seen any message that is free, at least I know they charge 1 shilling.” (Low interactor IDI#2)

### 3) Family MUAC supported by two-way SMS is an appropriate mechanism to connect with caregivers and to relay health messages

Both HCWs and caregivers perceived Family MUAC supported by two-way SMS as appropriate in rural and urban Kenyan contexts. Caregivers can take on the role of measuring and monitoring a child’s MUAC, even if they encountered some cultural and societal resistance. Caregivers were considered appropriate people to measure their child’s MUAC since they were in close proximity to the child throughout the day. A FGD participant shared that providing a MUAC tape allowed caregivers to track their child’s growth:

“For me I feel that mothers should be given the MUAC tape to take home because she is the one who will be measuring the child and she measures the child she will know whether the child is moving to the negative or the positive side. Apart from that a mother can even measure another child not just hers.” (FGD #4)

The use of SMS to send and receive measurements was preferred over other platforms, such as WhatsApp or Facebook, mainly due to the ability to send SMS without an internet connection. Connecting HCWs with caregivers via SMS messaging was perceived as appropriate and SMS messages were also tailored and relevant to the caregivers’ context. The cadence of weekly measurements was generally appropriate though some caregivers measured their children more frequently. A counselor expressed the appropriateness of weekly measurements by caregivers:

“You see weekly measurement was better because it was not tedious for the mothers, if we could tell them to measure the MUAC of their children daily it could be cumbersome and tedious but again if we could tell them to measure their children monthly, then it could be a long period so it was such a good interval for earlier tracking of the nutrition status of the participant.” – (Counselor IDI#3)

Some caregivers even preferred to receive daily messages as they found the content of SMS messages useful. One high interactor remarked, “I think [messages] can be sent even daily because it is helpful because maybe you are a person who probably didn’t go to school or do not know anything about health…then you learn from that [message] and take action.” (High interactor IDI#4)

### 4) Uptake of the Family MUAC is generally high, but can be compromised by individual and social-level barriers

Families in the intervention arm had high uptake of the intervention with 94% responding to one text message or more over the period of enrollment [8]. Caregivers in the intervention sent a median of 20 messages (interquartile range (IQR): 15–24) over the six-month enrollment period. Four determinants were identified as primarily driving caregiver uptake of Family MUAC supported by two-way text messaging including *environmental context and resources*, *memory/attention/decision processes*, *knowledge*, and *social influences*. Low interactors mentioned more barriers to uptake compared to high interactors. Barriers for uptake included 1) challenges with taking MUAC measurements, 2) negative social influences, and 3) phone issues and sharing. Barriers and facilitators for uptake of Family MUAC supported by two-way SMS are presented below.

Low interactors expressed frustration when multiple measurements were taken in the same sitting with inconsistent results. This confused caregivers as to what measurement to report to HCWs especially when measurements fell under different colors or numbers indicating that potential retraining is needed to improve accuracy and reliability of measurements. A low interactor mentioned “there was a way reading the number was a problem, sometimes I would get 14 sometimes 13.8 and sometimes 12 within the same measuring time so I didn’t know what to report” (Low interactor IDI#3)

In addition, low interactors reported the monotony of sending the same color measurements every week. These caregivers did not understand the advantage of monitoring MUAC measurements if a child was healthy. An example is given here:

“If I have measured and the child was in green the previous one, so it was green all the time I found that monotonous like there is no difference. So I never wanted to send…it reached a point I was getting tired of sending because of that monotony” (Low interactor IDI#3)

Low interactors perceived greater negative social stigma with measuring children compared to high interactors (TDF domain: *social influences*) (Table 3). Participants noted that many mothers-in-law of low interactors did not support caregivers enrolling into the program or measuring children (Table 3). In contrast, high interactors received more social support from other mothers, mothers-in-law, neighbors, husbands, and other children in the household leading to a higher uptake of family MUAC than low interactors. They benefited from a wider social network to consult for advice about their child’s health. One participant noted that:

**Table 3:** Additional supportive quotes for uptake of Family MUAC with 2-way SMS intervention.

TDF domain (construct)	Additional supportive quotes
Environmental context and resources (barriers)	“the challenges you can find is that today you have measured it is green, tomorrow you measure you find it has gone back to that yellow side, so you wonder why is it going up and coming down, so it used to go up and comes down.” – FGD #1
Social influences	“there are people who associate anything with HIV, so they might think that the child is HIV positive that is why the child is being measured.” – Low interactor IDI #4 “there was a lot of stigma especially with my mother in law. So at least then I used to feel like I was supported [by the Mama Aweza team], by the way they really helped me with my situation” – FGD #5 “Also my children usually reminded me, like “mum have you measured [child]” – FGD #7 “So anytime my mother saw me seated she would say ‘hey have you measured the child?’ then I go and get the tape, so my second child knew where the tape was kept and would rush to the bag so we get the tape. So the whole family was involved”. – FGD #1

“The father also got interested and joined… he would send the older child his name is [X], he will be like “X go and get the measuring tape so we can measure you and also measure [younger child]. Then I would tell him we have already measured the baby but he would insist he also wanted to measure. So I felt it was something good.” (High interactor IDI#1)

A supportive household environment encouraged greater engagement with Family MUAC and provided an external stimulus to measure children on a regular basis. Household members often reminded caregivers to measure their children.

Several high and low interactors mentioned sharing phones as a barrier to uptake of Family MUAC. Reading and sending messages were difficult when phones were shared with husbands who had different schedules. Sharing a phone presented an additional hurdle to send in measurements as caregivers needed access to the phone and their child at the same time to measure MUAC and send in measurements. Confidentiality was also a challenge for those with shared phones when HCWs exchanged direct SMS messages with caregivers.

High interactors stored MUAC tape in a specific place, which served to remind caregivers to measure their children (TDF domain: *memory, attention, decision processes*). One high interactor (#2) stated, “I was keeping one of the tapes in my bedroom, I had hanged it on the wall so every week when I wake up I would ask myself whether I had measured or not.” This decreased the chance of losing or misplacing the MUAC tape and reduced the time needed to measure the child.

### 5) Integrating Family MUAC supported by two-way SMS behaviors into routine practice made participation more feasible

Caregivers found Family MUAC supported by two-way SMS feasible to integrate into their routine and developed skills to measure a child quickly. The two TDF determinants influencing this theme include *skills* and *behavioral regulation*. High interactors were proactive to set a specific day and time of the week to measure their children. They incorporated child measurements into their weekly routine and anticipated the HCWs’ messages. Caregivers with non-acutely malnourished children were also able to develop a habit out of measuring their child compared to caregivers with acutely malnourished children. On the other hand, low interactors perceived taking MUAC measurements as a difficult task and remembered to measure their children only when they received reminder messages (Table 4). Generally, low interactors had challenges with incorporating measuring their child into routine practice.

**Table 4:** Additional supportive quotes for the feasibility of Family MUAC with 2-way SMS intervention.

TDF domain (construct)	Additional supportive quotes
Behavioral regulation (routine/automatic/habit)	“What made it easy for me, because I had already put it as one of my morning routines that I do every morning, so that that day in a week I just know one of the things I should do “ – FGD #6 “Mostly I wasn’t remembering unless I receive a message, I cannot lie.” – Low Interactor IDI #7 “In the program and they would send reminder messages and I would send the measurements every week and then now I became used to doing that and made it part and parcel of the things I do every week.” – High Interactor IDI #3 “Some respond… in the morning when we come there are some who responded overnight when we had already left. So, when we come in the morning, we will find the messages. Then in between there are people who prefer sending in the morning, maybe 9am or 10am after we started doing our duties. So, if at all there is no workload in the ground and screening, we will come in between and check the messages. In the afternoon also there are some who opted for the afternoon. So, you find that the SMS system is a continuous process.” – Counselor IDI #3
Skills	“It used to take me around 20 minutes but only if she is awake and is not cooperating, maybe she was playing and you are taking her away from her game, so sometimes she would cry and protest.” – Low Interactor IDI #4

Many caregivers measured their child before receiving the HCW’s reminder messages, so they could respond immediately when weekly messages were sent from the study - “I measured the baby in the evening around 6 to 7pm at least before the message comes.” – High interactor IDI#1

For high interactors, measuring a child was not a time burden (<5 minutes), requiring little or no effort once they learned strategies to calm a child for measurement (TDF domain: *behavioral regulation*), while it could take low interactors up to 20 minutes to measure their child (Table 4). High interactors reported developing *skills* to measure their child more than low interactors.

HCWs offered a different perspective on skills development. They mentioned that the initial training offered to caregivers on measuring MUAC was often insufficient to ensure high accuracy and reliability of measurements throughout the six-month study period. They emphasized the importance of building measurement skills through retraining. A HCW expressed:

“Now training them just once in the clinic and leave them to do the activity for the rest of the six months follow up was not such a good idea… we should have given them time to be going for refresher training of taking the MUAC at home because most of them when it came to [the] exit of the study, most of them were doing their own things.” (Counselor IDI#4)

HCWs recommended offering caregivers refresher training on MUAC measurements especially for caregivers who struggled to send in measurements on a regular basis.

In terms of feasibility of the two-way SMS system from the HCW perspective, many said it took between 30 mins – 1.5 hours to respond to messages daily depending on message load. For example, Monday morning would be busier as they were required to respond to messages that had accumulated over the weekend. Each day, HCWs would respond to 20–50 messages from caregivers (most of the time close to 30). Each provider established a workflow to read, respond to SMS messages, and access dashboard information that fit their schedules (Table 4). They checked the dashboard when they entered the office, responding to messages that came in the late evening of the previous day or early in the morning, at mid-day, and at the end of the work day.

### 6) Family MUAC empowered caregivers to track their child’s nutritional status and became health advocates in their communities

Caregivers equipped with MUAC tape felt empowered to track the health status of their child and share their new knowledge with others in their community. *Beliefs about capabilities* primarily influenced this theme. Caregivers recognized the ability of MUAC to track the nutrition status of children at the household and community level when MUAC measurements were taken on a regular basis. The MUAC tape was a tool to track growth and provided information on nutritional status to caregivers and HCW. One caregiver shared, “using the tape measure … will help me know how my child is doing in terms of nutrition for example if he is in colour yellow, green or red I will know the status of his health and I will know what that means” (FGD #3)

Caregivers felt empowered to share the skills and knowledge gained from the intervention with their neighbors and friends (TDF domain: *beliefs about capabilities*). Some caregivers were recognized as leaders in their community because of the new skills they acquired in measuring MUAC, which motivated them to measure other children and advise others within their community. In one FGD, a participant mentioned, “when I had the MUAC tape, I would see a child somewhere and I’d tell the mother to bring the child [so that] I measure the arm for her, and if I found the child was in yellow or red I would advise her to urgently take the child to the hospital.” (FGD #4)

Women equipped with MUAC tapes started counseling other women who had malnourished children. They used these newly developed skills to educate others on the messages they received through SMS messages but also on the benefits of tracking a child’s nutrition status over time (Table 5). One caregiver in an FGD mentioned, “if I see a child losing weight I will advise the mother to give the child a balanced diet or I simply discuss with her the problem … because now I know the child should be taken to hospital.” (FGD#5)

**Table 5:** Additional supportive quotes for Family MUAC with 2-way SMS intervention empowering caregivers.

TDF domain (construct)	Additional supportive quotes
Beliefs about capabilities	“This is about the health of my child‘s so that at least it helps you know if the child is okay or the child is reducing weight or his weight is increasing” – FGD #1 “When you are given the MUAC tape even your neighbor can learn from it, you can educate her on the benefits and she can also spread the message by educating other neighbors so this tape is very important because it will be like you have helped the people around there” – FGD #2

### 7) HCWs reported that integrating Family MUAC into other health services and daily routine would increase sustainability

HCWs perceived Family MUAC supported by two-way SMS as sustainable given that it reduced their workload, allowed for timely follow up of patients, and stored patient information securely. For example, a nutritionist expressed:

“I would want to use the system [if it was available in the future] because it will make my work easier by reducing the workload, it will help me with following up my clients, the information is safe because the system is having a password…so, the information of the clients will be confidential.” (Nutritionist IDI#3)

HCWs suggested that integrating Family MUAC supported by two-way SMS with other health services would lead to greater sustainability. For example, if women visit health centers for HIV/AIDS services, they can benefit from enrolling in family MUAC and receive integrated SMS messages about tracking their child’s nutrition. A study counselor (IDI#5) mentioned “a mother coming for a prevention of mother-to-child transmission of HIV (PMTCT) service and also coming for [MAMMS] so we could take the initiative of taking her to the PMTCT to fasten the service and then help us make other follow ups faster.” This suggests that family MUAC can be introduced to patients who are already visiting health centers for other services.

## Discussion

Study findings demonstrate that Family MUAC supported with two-way SMS is perceived as feasible, acceptable, cost-saving, and appropriate for empowering caregivers to monitor childhood acute malnutrition at the household level in western Kenya. Caregivers used the mHealth tool to facilitate timely communication with HCWs, who provided guidance and remote counsel. Key drivers of program uptake included a supportive social environment, phone ownership, and adequate training in MUAC measurement techniques. Conversely, barriers such as phone sharing, lack of social support, and inconsistent training were reported, reflecting challenges similar to those described in other studies [18–21].

The use of the mHealth platform to provide remote support was particularly effective in addressing logistical barriers. Caregivers emphasized that the free SMS feature reduced financial and time burdens associated with clinic visits, enabling them to seek timely advice while minimizing transportation and related costs. These findings are consistent with prior studies showing that mHealth interventions can reduce out-of-pocket expenditures and improve access to care by enabling remote consultations and follow-up [22,23], particularly in rural and hard-to-reach settings [24,25]. In many LMIC contexts, geographic distance and transportation challenges are key deterrents to care seeking, and mHealth solutions have been shown to mitigate these barriers by facilitating timely communication between caregivers and providers [25]. In Kenya, mHealth solutions to support healthcare delivery have been outlined in the National eHealth Policy and can be a key strategy to reach rural areas across the country taking advantage of the high coverage of Kenya’s mobile networks [26].

From the perspective of HCWs, the intervention also contributed to more efficient service delivery. Remote guidance reduced the need for home visits in non-urgent cases, allowing HCWs to allocate time and resources more effectively. Moreover, having direct access to HCWs via SMS increased caregivers’ acceptance of the program and fostered improved patient-provider relationships, which has been identified as a key determinant of sustained engagement in digital health interventions [27]. These results are consistent with existing literature on mHealth interventions, which frequently report improvements in caregiver-HCW communication, adherence to health recommendations, cost savings, and reduced healthcare burdens [27–30].

The intervention also had a notable impact on caregiver empowerment. Caregivers reported increased confidence with monitoring their child’s health and became health educators within their communities, a finding supported by previous studies in South Africa and Niger using a Family MUAC approach [7,31]. Equipped with MUAC tapes and new knowledge and skills, caregivers also measured neighborhood children and provided health advice and support to neighbors, suggesting the intervention’s potential to indirectly promote health awareness and engagement beyond the immediate beneficiaries. These findings highlighting the capacity of community-based health programs to strengthen caregiver confidence and participation in health advocacy [20,30]. By fostering this sense of empowerment, Family MUAC interventions like Mama Aweza could potentially contribute to broader public health goals, including the promotion of gender equality and community resilience [20].

Despite these benefits, concerns regarding the accuracy and consistency of MUAC measurements emerged as a challenge. While many caregivers reported confidence in their ability to perform the measurements, concerns about consistency and accuracy were common. Since training on MUAC use was only provided at study onset, low interactors expressed frustration and confusion resulting from inconsistencies in measurement, which may have contributed to lower levels of engagement with the program. Ongoing training and regular refresher sessions could help to enhance the reliability of caregiver-collected data. HCWs emphasized the need for continuous guidance to address caregiver questions and ensure accurate measurements. Prior studies have also underscored the necessity of sustained training in community-based health interventions to maintain data integrity and program effectiveness, and to improve sustainability [19,31–34]. At the same time, providing ongoing support through two-way SMS platforms may introduce additional demands on HCWs, particularly in terms of time required to review and respond to caregiver messages. Without careful program design, including appropriate staffing, task-shifting strategies, or automation of routine responses, these added responsibilities could increase workload and potentially limit the feasibility and scalability of such interventions within already resource-constrained health systems.

Technology access was another critical factor influencing program uptake. Caregivers without personal phones often relied on shared devices, which limited their ability to engage fully in the program. This challenge was particularly pronounced when the primary phone owner was away from the caregiver or child. Similar barriers have been identified in other mHealth studies [18,20,35,36], where limited device ownership and concerns about privacy when using shared phones hinder participant engagement [36,37]. These findings underscore the need to consider technological access when designing scalable interventions. Evidence from other digital health programs suggests that improving access to mobile devices through subsidized or low-cost provision is critical for maximizing participation and ensuring the effectiveness of mHealth interventions [23].

Social and cultural dynamics within households further influenced caregiver engagement. While some caregivers benefited from supportive family environments, others reported resistance or skepticism from key decision-makers, including spouses and mothers-in-law. Caregivers with supportive family members were more likely to remain active in the program, while those facing negative social influences or lacking support experienced difficulties maintaining participation. Additionally, prevailing gender norms that position women as primary caregivers may increase their responsibility for child health while limiting their autonomy in decision-making or access to resources [38]. Evidence from maternal and child nutrition programs suggests that engaging additional family members during program implementation, including male partners and senior female relatives, could enhance caregiver involvement and overall program success [21]. Approaches such as grandmother or husband support groups, digital health interventions that engage non-primary caregiver household members, and facility-based interventions such as couples counseling sessions during routine health visits could increase involvement and engagement in child nutrition programs [21]. Practical strategies observed in our study, such as designating a consistent location for MUAC tapes and integrating measurements into daily routines, further illustrate how household-level adaptations can support sustained uptake and engagement.

These findings can also be interpreted through a self-care lens. Family MUAC supported by two-way SMS represents a form of guided self-care, where caregivers are empowered to monitor and respond to their child’s health needs with remote support from the health system. For caregivers with adequate resources, knowledge, and social support, this model can enhance self-efficacy, promote timely care-seeking, and reduce reliance on facility-based services [39]. However, for caregivers facing structural constraints, including limited access to mobile technology, low literacy, or competing financial demands, such interventions may inadvertently increase burden or exacerbate existing inequities [39]. This dual potential highlights the need for careful consideration of equity in the design and implementation of self-care interventions, ensuring that additional support mechanisms are in place for those with fewer resources.

This study has limitations. Experiences from HCWs from the three referral hospitals in Kenya may not be representative of other areas in Kenya or globally. Additionally, the reliance on baseline training for caregivers introduced the potential for measurement errors, highlighting the need for ongoing skill reinforcement. Dependence on SMS technology also presents challenges for scalability in areas with limited access to mobile phones or network coverage [35]. We did not collect detailed socio-demographic characteristics from participants, which limits our ability to assess how factors such as literacy, socioeconomic status, or access to broader household resources may have influenced engagement with the intervention. Although we purposively sampled participants across sites and levels of engagement, the absence of these data restricts our ability to examine potential differences between groups. Consideration of these characteristics could provide further insights into barriers to engagement. In addition, the study enrolled caregivers who were literate or had access to someone to help them read and send SMS, which may limit transferability to other contexts with lower literacy rates or less access to mobile phones. Moreover, we collected perceptions about cost savings from caregivers but do not report on a full economic evaluation to quantify these perceived cost savings. A cost-effectiveness analysis or cost-benefit analysis could provide further evidence of cost savings. Addressing these limitations will be critical for adapting and scaling the intervention to diverse populations and contexts.

## Conclusions

In summary, this study provided evidence supporting the Family MUAC with two-way SMS intervention as an acceptable, feasible, and appropriate community-driven approach to reduce child wasting. The intervention empowered caregivers as active participants in health promotion activities. Providing guided support to improve the consistency and accuracy of caregiver-led measurements and identifying and empowering community champions of Family MUAC could promote sustainability of this intervention. These findings align with the Global Action Plan on Child Wasting that emphasizes community engagement, capacity building, and gender empowerment in support of the Sustainable Development Goals.

## Supporting information

S1 Checklist Consolidated criteria for reporting qualitative studies (COREQ) checklist. (DOCX)

S1 File Inclusivity in global research questionnaire. (DOCX)
