# Supplementary material for: Acceptability, feasibility, appropriateness, uptake and cost perceptions of family mid-upper arm circumference supported by two-way SMS in western Kenya
Source: PLoS One. 2026 Sep 23;21(9):e0358775. doi: 10.1371/journal.pone.0358775 (PMC13600513; doi:10.1371/journal.pone.0358775)
Supplement: Supplementary file 2 [file pone.0358775.s005.pdf]

PLOS One

Acceptability, feasibility, appropriateness, uptake and cost perceptions of Family MUAC supported by two-way SMS in western Kenya

--Manuscript Draft--

**Manuscript Number:**	PONE-D-25-63080
**Article Type:**	Research Article
**Full Title:**	Acceptability, feasibility, appropriateness, uptake and cost perceptions of Family MUAC supported by two-way SMS in western Kenya
**Short Title:**	Acceptability, feasibility, appropriateness, uptake, cost perceptions of Family MUAC with SMS
**Corresponding Author:**	Arianna R Means, Ph.D., MPH<br>University of Washington Seattle Campus: University of Washington<br>Seattle, WA UNITED STATES OF AMERICA
**Keywords:**	Family MUAC; digital health; Malnutrition; two-way SMS; Kenya
**Abstract:**	Early identification and treatment of acute malnutrition can decrease wasting-related mortality and morbidity. Teaching caregivers to measure mid-upper arm circumference (MUAC), an approach known as Family MUAC, may support early diagnosis of wasting. Family MUAC paired with mobile support from healthcare workers resulted in a 37% reduction in child wasting in the Mama Aweza trial. This study examines the intervention's acceptability, feasibility, appropriateness, uptake, cost savings, and factors influencing engagement with Family MUAC.<br>We conducted eight focus group discussions with caregivers enrolled in the trial and fourteen in-depth interviews with caregivers who participated in the intervention arm, Family MUAC supported by two-way short message system (SMS). Caregivers were purposively selected for interviews based on engagement level with the intervention. Additionally, eighteen program staff and health workers from three facilities in western Kenya participated in in-depth interviews. Data collection involved semi-structured guides informed by the Theoretical Domains Framework.<br>Caregiver and health worker perceptions of Family MUAC with two-way SMS centered on seven themes. The intervention was widely viewed as acceptable, cost-saving, appropriate, feasible, and empowering. Caregiver uptake was high, and integrating child growth monitoring with other health services may help enhance sustainability. Barriers to uptake included inconsistent MUAC measurements, limited social support, and issues with phone sharing. Refresher trainings could increase accuracy of MUAC measurements and improve engagement. Direct communication with healthcare workers via SMS built trust, improved communication, and strengthened patient-provider relationships. Caregivers also reported cost savings, as SMS communication reduced unnecessary travel to health centers when children were not malnourished.<br>This study supports Family MUAC with two-way SMS as a promising approach to empower caregivers with tools to prevent malnutrition and highlights their critical role in child health and nutrition interventions. Future interventions can focus on improving measurement accuracy and mitigating negative social influences on caregiver-led interventions.
**Order of Authors:**	Esther M Choo
	Jonathan Lara-Arevalo
	Catherine Achieng
	Merceline Odhiambo
	Maurine Anyango Okello
	Mary Masheti
	Kirkby D. Tickell
	Mame M. Diakhate
	Benson O. Singa

Powered by Editorial Manager® and ProduXion Manager® from Aries Systems Corporation

	Christine J. McGrath
	Arianna Rubin Means
**Additional Information:**	
**Question**	**Response**
**Financial Disclosure**<br><br>Enter a financial disclosure statement that describes the sources of funding for the work included in this submission. Review the submission guidelines for detailed requirements. View published research articles from *PLOS ONE* for specific examples.<br><br>This statement is required for submission and **will appear in the published article** if the submission is accepted. Please make sure it is accurate.<br><br>**Funded studies**<br>Enter a statement with the following details:<br>• Initials of the authors who received each award<br>• Grant numbers awarded to each author<br>• The full name of each funder<br>• URL of each funder website<br>• Did the sponsors or funders play any role in the study design, data collection and analysis, decision to publish, or preparation of the manuscript?<br><br>Did you receive funding for this work?	Yes
Please add funding details.<br>as follow-up to "**Financial Disclosure**<br><br>Enter a financial disclosure statement that describes the sources of funding for the work included in this submission. Review the submission guidelines for detailed requirements. View published research articles from *PLOS ONE* for specific examples.<br><br>This statement is required for submission and **will appear in the published article** if the submission is accepted. Please make	This work was supported by the Thrasher Research Foundation (ID#14656) and an unrestricted gift from Pamela H. Fowler.

sure it is accurate.<br><br>**Funded studies**<br>Enter a statement with the following details:<br>• Initials of the authors who received each award<br>• Grant numbers awarded to each author<br>• The full name of each funder<br>• URL of each funder website<br>• Did the sponsors or funders play any role in the study design, data collection and analysis, decision to publish, or preparation of the manuscript?<br><br>Did you receive funding for this work?"	
Please select the country of your main research funder (please select carefully as in some cases this is used in fee calculation).<br>as follow-up to "**Financial Disclosure**<br><br>Enter a financial disclosure statement that describes the sources of funding for the work included in this submission. Review the submission guidelines for detailed requirements. View published research articles from *PLOS ONE* for specific examples.<br><br>This statement is required for submission and **will appear in the published article** if the submission is accepted. Please make sure it is accurate.<br><br>**Funded studies**<br>Enter a statement with the following details:<br>• Initials of the authors who received each award<br>• Grant numbers awarded to each author<br>• The full name of each funder<br>• URL of each funder website<br>• Did the sponsors or funders play any role in the study design, data collection and analysis, decision to publish, or preparation of the manuscript?<br><br>Did you receive funding for this work?"	UNITED STATES - US

Powered by Editorial Manager® and ProduXion Manager® from Aries Systems Corporation

**Competing Interests**<br><br>Use the instructions below to enter a competing interest statement for this submission. On behalf of all authors, disclose any competing interests that could be perceived to bias this work—acknowledging all financial support and any other relevant financial or non-financial competing interests.<br><br>This statement is required for submission and **will appear in the published article** if the submission is accepted. Please make sure it is accurate and that any funding sources listed in your Funding Information later in the submission form are also declared in your Financial Disclosure statement.<br><br>View published research articles from *PLOS ONE* for specific examples.<br><br>**NO authors have competing interests**<br><br>Enter: *The authors have declared that no competing interests exist.*<br><br>**Authors with competing interests**<br><br>Enter competing interest details beginning with this statement:<br><br>*I have read the journal's policy and the authors of this manuscript have the following competing interests: [insert competing interests here]*<br><br>* typeset	The authors have declared that no competing interests exist
**Ethics Statement**<br><br>Enter an ethics statement for this submission. This statement is required if the study involved:<br><br>• Human participants<br>• Human specimens or tissue<br>• Vertebrate animals or cephalopods	The study protocol received ethical approval from the University of Washington (STUDY00006221) and the Kenya Medical Research Institute (SERU 3821, V.1.4, 24 January 2020). All participants provided written informed consent in their preferred language (Kiswahili, Luo, Kuria, or English).

Powered by Editorial Manager® and ProduXion Manager® from Aries Systems Corporation

• Vertebrate embryos or tissues<br>• Field research<br><br>Write "N/A" if the submission does not require an ethics statement.<br><br>General guidance is provided below. Consult the submission guidelines for detailed instructions. **Make sure that all information entered here is included in the Methods section of the manuscript.**	

**Format for specific study types**<br><br>**Human Subject Research (involving human participants and/or tissue)**<br>• Give the name of the institutional review board or ethics committee that approved the study<br>• Include the approval number and/or a statement indicating approval of this research<br>• Indicate the form of consent obtained (written/oral) or the reason that consent was not obtained (e.g. the data were analyzed anonymously)<br><br>**Animal Research (involving vertebrate animals, embryos or tissues)**<br>• Provide the name of the Institutional Animal Care and Use Committee (IACUC) or other relevant ethics board that reviewed the study protocol, and indicate whether they approved this research or granted a formal waiver of ethical approval<br>• Include an approval number if one was obtained<br>• If the study involved *non-human primates*, add *additional details* about animal welfare and steps taken to ameliorate suffering<br>• If anesthesia, euthanasia, or any kind of animal sacrifice is part of the study, include briefly which substances and/or methods were applied<br><br>**Field Research**<br><br>Include the following details if this study involves the collection of plant, animal, or other materials from a natural setting:<br>• Field permit number<br>• Name of the institution or relevant body that granted permission	
**Data Availability**<br><br>Authors are required to make all data underlying the findings described fully available, without restriction, and from the time of publication. PLOS allows rare exceptions to address legal and ethical concerns. See the PLOS Data Policy and FAQ for detailed information.	Yes - all data are fully available without restriction

A Data Availability Statement describing where the data can be found is required at submission. Your answers to this question constitute the Data Availability Statement and **will be published in the article**, if accepted.<br><br>**Important:** Stating 'data available on request from the author' is not sufficient. If your data are only available upon request, select 'No' for the first question and explain your exceptional situation in the text box.<br><br>Do the authors confirm that all data underlying the findings described in their manuscript are fully available without restriction?	
**Describe where the data may be found in full sentences. If you are copying our sample text, replace any instances of XXX with the appropriate details.**<br><br>• If the data are **held or will be held in a public repository**, include URLs, accession numbers or DOIs. If this information will only be available after acceptance, indicate this by ticking the box below. For example: *All XXX files are available from the XXX database (accession number(s) XXX, XXX.).*<br>• If the data are all contained **within the manuscript and/or Supporting Information files**, enter the following: *All relevant data are within the manuscript and its Supporting Information files.*<br>• If neither of these applies but you are able to provide **details of access elsewhere**, with or without limitations, please do so. For example:<br><br>*Data cannot be shared publicly because of [XXX]. Data are available from the XXX Institutional Data Access / Ethics Committee (contact via XXX) for researchers who meet the criteria for access to confidential data.*<br><br>*The data underlying the results presented in the study are available from (include the name of the third party*	The datasets used and/or analysed during the current study are available from the corresponding author upon request

Powered by Editorial Manager® and ProduXion Manager® from Aries Systems Corporation

and contact information or URL).<br>• This text is appropriate if the data are owned by a third party and authors do not have permission to share the data.<br><br>* typeset	
Additional data availability information:	Tick here if your circumstances are not covered by the questions above and you need the journal's help to make your data available.

Click here to access/download;Manuscript;MamaAweza_qual manuscript_PLOSOne 12_15_2025.docx

Full title: Acceptability, feasibility, appropriateness, uptake and cost perceptions of Family MUAC supported by two-way SMS in western Kenya

Short title: Acceptability, feasibility, appropriateness, uptake, cost perceptions of Family MUAC with SMS

Esther M. Choo[1], Jonathan Lara-Arevalo[2], Catherine Achieng[3], Merceline Odhiambo[4], Maurine Anyango Okello[3], Mary Masheti[3], Kirkby D. Tickell[5], Mame M. Diakhate[5], Benson O. Singa[3], Christine J. McGrath[5], Arianna Rubin Means[5*]

[1] Independent Consultant, Seattle, USA

[2] Department of Nutrition, Gillings School of Global Public Health, University of North Carolina at Chapel Hill, Chapel Hill, North Carolina, USA

[3] Centre for Clinical Research, Kenya Medical Research Institute, Nairobi, Kenya

[4] Independent Consultant, Nairobi, Kenya

[5] Department of Global Health, University of Washington, Seattle, Washington, USA

*Corresponding author:

Email: aerubin@uw.edu (ARM)

Abstract

Early identification and treatment of acute malnutrition can decrease wasting-related mortality and morbidity. Teaching caregivers to measure mid-upper arm circumference (MUAC), an approach known as Family MUAC, may support early diagnosis of wasting. Family MUAC paired with mobile support from healthcare workers resulted in a 37% reduction in child wasting in the Mama Aweza trial. This study examines the intervention's acceptability, feasibility, appropriateness, uptake, cost savings, and factors influencing engagement with Family MUAC.

We conducted eight focus group discussions with caregivers enrolled in the trial and fourteen in-depth interviews with caregivers who participated in the intervention arm, Family MUAC supported by two-way short message system (SMS). Caregivers were purposively selected for interviews based on engagement level with the intervention. Additionally, eighteen program staff and health workers from three facilities in western Kenya participated in in-depth interviews. Data collection involved semi-structured guides informed by the Theoretical Domains Framework.

Caregiver and health worker perceptions of Family MUAC with two-way SMS centered on seven themes. The intervention was widely viewed as acceptable, cost-saving, appropriate, feasible, and empowering. Caregiver uptake was high, and integrating child growth monitoring with other health services may help enhance sustainability. Barriers to uptake included inconsistent MUAC measurements, limited social support, and issues with phone sharing. Refresher trainings could increase accuracy of MUAC measurements and improve engagement. Direct communication with healthcare workers via SMS built trust, improved communication, and

strengthened patient-provider relationships. Caregivers also reported cost savings, as SMS communication reduced unnecessary travel to health centers when children were not malnourished.

This study supports Family MUAC with two-way SMS as a promising approach to empower caregivers with tools to prevent malnutrition and highlights their critical role in child health and nutrition interventions. Future interventions can focus on improving measurement accuracy and mitigating negative social influences on caregiver-led interventions.

Introduction

Nearly half of all deaths of children under five years of age are linked to undernutrition, primarily in low-and-middle-income countries (LMICs) [1]. Over 45 million children are estimated to be wasted or acutely malnourished (low weight-for-height) globally, which can increase the risk of death amongst children under five by 12-fold [2]. Yet, it is estimated that only 1 in 3 children with wasting receive treatment [3]. Early identification of undernutrition and initiation of treatment can prevent wasting, and the World Health Organization (WHO) recommends integrating early detection and treatment of child wasting into routine primary health care services [4].

One approach to early detection and treatment is engaging with caregivers during their child's infancy and empowering them with the knowledge and tools needed to measure their child's mid-upper arm circumference (MUAC) at home, an approach known as Family MUAC. In some settings, home-based monitoring of a child's MUAC by caregivers has led to earlier identification of malnutrition in comparison to active and passive screening by field workers [5, 6]. We conducted a randomized controlled trial to test the effectiveness of a caregiver-administered MUAC monitoring system with direct communication to healthcare workers via short message system (SMS). The study found that Family MUAC supported by two-way SMS was associated with a 37% reduction in wasting in children 5-12 months compared to standard of care in western Kenya [7].

Effectively engaging in Family MUAC supported by an mHealth platform requires a series of caregiver and health worker behaviors, including retaining knowledge on correct MUAC tape use, regularly using MUAC tapes at home, sending and responding to text message prompts to share

MUAC measures, following-up at health centers when prompted to do so (e.g. in the event of a concerning MUAC measurement), and completing treatments when wasted. While there is considerable evidence regarding drivers of caregiver behaviors related to infant and young child feeding [8] and care seeking behaviors generally [9, 10], there is minimal information about determinants of Family MUAC behaviors. This evidence is necessary for designing Family MUAC programs at scale that are culturally and contextually appropriate, and most likely to prompt targeted behaviors.

To address this evidence gap, we conducted an explanatory sequential mixed methods study to understand determinants of Family MUAC behaviors in study sites in western Kenya. We studied caregivers whose children became acutely malnourished, whose children did not develop malnutrition, who had high engagement in the Family MUAC platform, and who had low engagement in the Family MUAC platform. The objectives of the caregiver focus group discussions (FGDs) and interviews were to assess acceptability, feasibility, appropriateness, uptake, and cost savings of Family MUAC supported by two-way SMS and drivers of engagement with the platform. This includes understanding drivers of high and low uptake of Family MUAC supported by two-way SMS and the comparative benefits and challenges of using these tools. We also engaged health workers to understand perceived acceptability, feasibility, appropriateness, and costs of implementing Family MUAC as well as opportunities to improve Family MUAC if adopted by governments as part of broader child nutrition services.

Materials and Methods

Study Design

We employed a mixed-methods explanatory sequential design (quan → QUAL) to investigate the determinants of engagement with Family MUAC supported by two-way SMS intervention for purposes of development and expansion [11]. We used qualitative data to identify and compare responses between caregivers whose children did and did not become acutely malnourished during the study. Additionally, we compared caregivers with high and low engagement (high interactors and low interactors) with the SMS platform. Furthermore, we interviewed health workers involved in the Mama Aweza trial to assess acceptability, feasibility, and uptake of Family MUAC supported by SMS. Study details are reported in accordance with the COREQ checklist [12] to ensure transparency (Supplementary Material 1).

The Mama Aweza Trial

This analysis was conducted as part of the Mama Aweza trial, a randomized controlled trial conducted in Homa Bay and Migori Counties in Kenya. The trial aimed to evaluate the efficacy of family MUAC combined with a two-way SMS platform, called the Maternally Administered Malnutrition Monitoring System (MAMMS), in preventing wasting compared to standard of care (SOC) for children aged 5-12 months. The trial recruited caregivers of children visiting a maternal and child health clinic at the Migori County Referral and Teaching Hospital, St. Joseph's Mission Hospital, and Homa Bay Teaching and Referral Hospital between August 2019 and January 2022. Caregivers of children with MUAC measurements between 12.5 and 14.0 cm were eligible to participate.

The trial compared two groups: both groups received training on how to measure their child's MUAC. The intervention group was provided with two MUAC tapes and received weekly SMS reminders to measure and report MUAC, along with education messages. The SOC group did not receive MUAC tapes or SMS support. The weekly health-related educational SMS messages were tailored to the child's developmental stage and encouraged caregivers to report their child's measurement either with the MUAC measurement color (red, yellow, green) or MUAC measurement number. When caregivers reported a child with a red or yellow measure, or MUAC below 12.5 cm, study staff replied with a message to bring the child to the health facility for care. In contrast, children in the SOC group were screened for malnutrition during study follow-up visits every three months. Follow up assessments at 6 months post enrollment captured the primary outcome, time-to-diagnosis of confirmed wasting (MUAC <11.5 cm). Children diagnosed with wasting were provided treatment according to Kenya's National Guidelines, with their progress monitored through scheduled follow-up visits. More information about the Mama Aweza trial design can be found elsewhere [7, 13].

Participant sampling

After study exit, a purposive sample of caregivers were invited to participate in face-to-face summative FGDs and in-depth interviews (IDI) from Migori County Referral and Teaching Hospital between November 29 – December 3, 2021, and from Homa Bay Teaching and Referral Hospital between December 7 – 9, 2021. We recruited caregivers with a child identified as acutely malnourished during the study, and caregivers with children who did not develop acute malnutrition for participation in FGDs from each study group. We aimed to conduct eight FGDs each with 5-8 participants. Additionally, IDIs with caregivers from the intervention group were

conducted to assess drivers of engagement with the platform. We defined high interactors as caregivers who responded to >90% of the automated messages with MUAC measurements within seven days, while low interactors were caregivers who responded to <30% of the automated messages with MUAC measurements within seven days. We invited participants once to participate in IDIs or FGDs.

Furthermore, IDIs were conducted with purposively sampled program staff and health workers active at Migori County Referral and Teaching Hospital and St. Joseph's Mission Hospital between June 27 – 29, 2022, and Homa Bay Teaching and Referral Hospital on June 30, 2022. The individual interviews were used to assess health worker perceptions of Family MUAC supported by two-way SMS, potential barriers to sustaining or scaling the program, and opportunities to improve the program moving forward.

Data collection

All FGDs and IDIs were conducted in the local language (Kiswahili, Luo, or Kuria), audio-recorded, transcribed, and translated into English by MO, a Kenyan qualitative Research Assistant (RA). MO is a female social scientist with a bachelor’s degree in Sociology and Anthropology with over ten years of experience in collecting qualitative data. The interviewer did not have any relationship or contact with study participants prior to study commencement. During enrollment of study participants, the RA shared the purpose of the study and participants were offered the opportunity to follow up with the Kenya based research team if they had feedback after the IDI or FGD concluded. All participants provided written informed consent in their preferred language (Kiswahili, Luo, Kuria, or English). All FGDs and IDIs were conducted in a private room within the

hospital. The RA completed a debrief form within 24 hours of completing each FGD or IDI to provide a summary of each session.

The Theoretical Domains Framework

We used the Theoretical Domains Framework (TDF) to inform data collection and analysis. The TDF is an integrative framework that draws from 33 behavior change theories to characterize and evaluate behavioral determinants of implementation. The TDF comprises 14 domains with a total of 84 constructs across domains [14]. All 14 domains from the TDF were used to inform the semi-structured FGD and IDI interview guides developed by the research team. The TDF was also used to guide the development of the codebook.

Data Management and Analysis

Audio recordings from the FGDs and IDIs were initially transcribed in the language in which they were conducted and then translated into English. Site investigators who did not perform the original transcriptions conducted two one-minute random spot checks per IDI or FGD. During these spot checks, the audio recordings and transcribed materials were compared to verify transcription quality. Any discrepancies found during the audio-to-transcription comparison prompted a full review of the transcript.

We employed inductive, deductive coding and content analysis. We employed an *a priori* list of the 14 TDF-based domains, with 20 constructs embedded under the domains, as the foundation for the codebook. The codebook was structured using broader axial codes and sub-codes. Members of the research team read each transcript to familiarize themselves with the content before beginning the line-by-line coding process using Dedoose-9.2.012. Two primary coders,

one based in Seattle, USA (JLA) and the other in Migori, Kenya (CA), conducted the coding. The first two transcripts were fully coded by both primary coders. When new codes emerged that were not covered in the initial codebook, inductive thematic coding was applied, creating and adding new codes to the codebook. These new codes were discussed in consensus meetings, involving at least one additional researcher. The third researcher (EMC) acted as a tiebreaker when consensus could not be reached involving a coded statement. During these meetings, code definitions were revised, inclusion and exclusion criteria for code application were defined, overlapping codes were combined, and codes were added or removed as necessary.

After incorporating new codes from the first two transcripts, the coders proceeded with the remaining transcripts. The coding process was conducted in two rounds. In the first round, transcripts were equally divided between the coders, who independently read and coded their assigned transcripts. In the second round, the coders switched transcripts and independently read and coded their new assignments. A detailed management table was maintained to track transcript assignments.

Upon completion of the coding, differences in code application were discussed to reach a consensus on the final coding scheme. Researchers then organized coded content into thematic topics, and patterns in relevant topics were accessed to identify key themes that answered the research questions. Key themes were identified by examining concepts that were prevalent across FGDs or IDIs or highlighted areas of convergence or divergence among participants. Case memos were used to summarize the patterns identified through coding. TDF determinants coded through the analysis were organized under key themes that emerged and visualized through a

conceptual framework. The final themes were discussed among coders and the research team to ensure the conceptual framework captured the main factors influencing acceptability, feasibility, appropriateness, uptake, and cost savings of Family MUAC supported by two-way SMS. Relative comparisons were noted based on how often determinants were mentioned and compared between different caregiver strata to understand drivers of engagement. For example, we compared how often TDF constructs were mentioned by high interactors and low interactors to determine barriers and facilitators to uptake of the intervention.

Ethics

The study protocol received ethical approval from the University of Washington (STUDY00006221) and the Kenya Medical Research Institute (SERU 3821, V.1.4, 24 January 2020). An independent Data and Safety Monitoring Board composed of experts in pediatrics, nutrition program implementation, and trial methods was responsible for study oversight. All interviews were confidential, and all names or identifying information were removed from transcripts to protect the identity of the participants and their associated institutions.

Results

We conducted eight FGDs (51 total FGD participants) and fourteen IDIs with female caregivers. Among the eight FGDs, two focus groups were carried out for each of the following sets of caregivers: (1) caregivers in the intervention group whose child did not develop acute malnutrition, (2) caregivers in the intervention group whose child became acutely malnourished,

(3) SOC participants whose child did not develop acute malnutrition, and (4) SOC participants whose child became acutely malnourished over the duration of the study. FGDs took between 40 to 104 minutes. Seven high interactors and seven low interactors participated in IDIs which lasted 24 to 43 minutes. All invited participants agreed to participate.

Additionally, 18 healthcare workers (HCW) participated in IDIs. Overall, 39% were community health workers or counselors, 28% were nurses, 11% were nutritionists engaged in the study, and 22% were Kenyan Ministry of Health clinical staff. HCW interviews ran 25 to 85 minutes.

We identified seven themes driving caregiver and health worker perceptions of Family MUAC supported by two-way SMS. These themes relate to the acceptability, cost savings, appropriateness, uptake, feasibility, empowerment and sustainability of the intervention. The main themes were: 1) Family MUAC had high acceptability among caregivers and HCWs and strengthened the family-clinician relationship, 2) perceptions of costs savings contributed to increased acceptability of Family MUAC, 3) Family MUAC supported by two-way SMS is an appropriate mechanism to connect with caregivers and to relay health messages, 4) uptake of the Family MUAC is generally high, but can be compromised by individual and social-level barriers, 5) integrating Family MUAC supported by two-way SMS behaviors into routine practice made participation more feasible, 6) Family MUAC empowered caregivers to track their child's nutritional status and became health advocates in their communities, and 7) HCWs reported that integrating Family MUAC into other health services and daily routine would increase sustainability.

The most influential TDF ~~domains~~ supporting these themes included beliefs about consequences, knowledge, reinforcement, skills, beliefs about capability, social influences, environment context and resources, memory attention and decision processes, behavioral regulation, and emotion (Fig 1). The determinants grouped under each theme are described in more detail below.

Figure 1: Conceptual framework of determinants driving caregiver engagement with family MUAC supported by two-way SMS

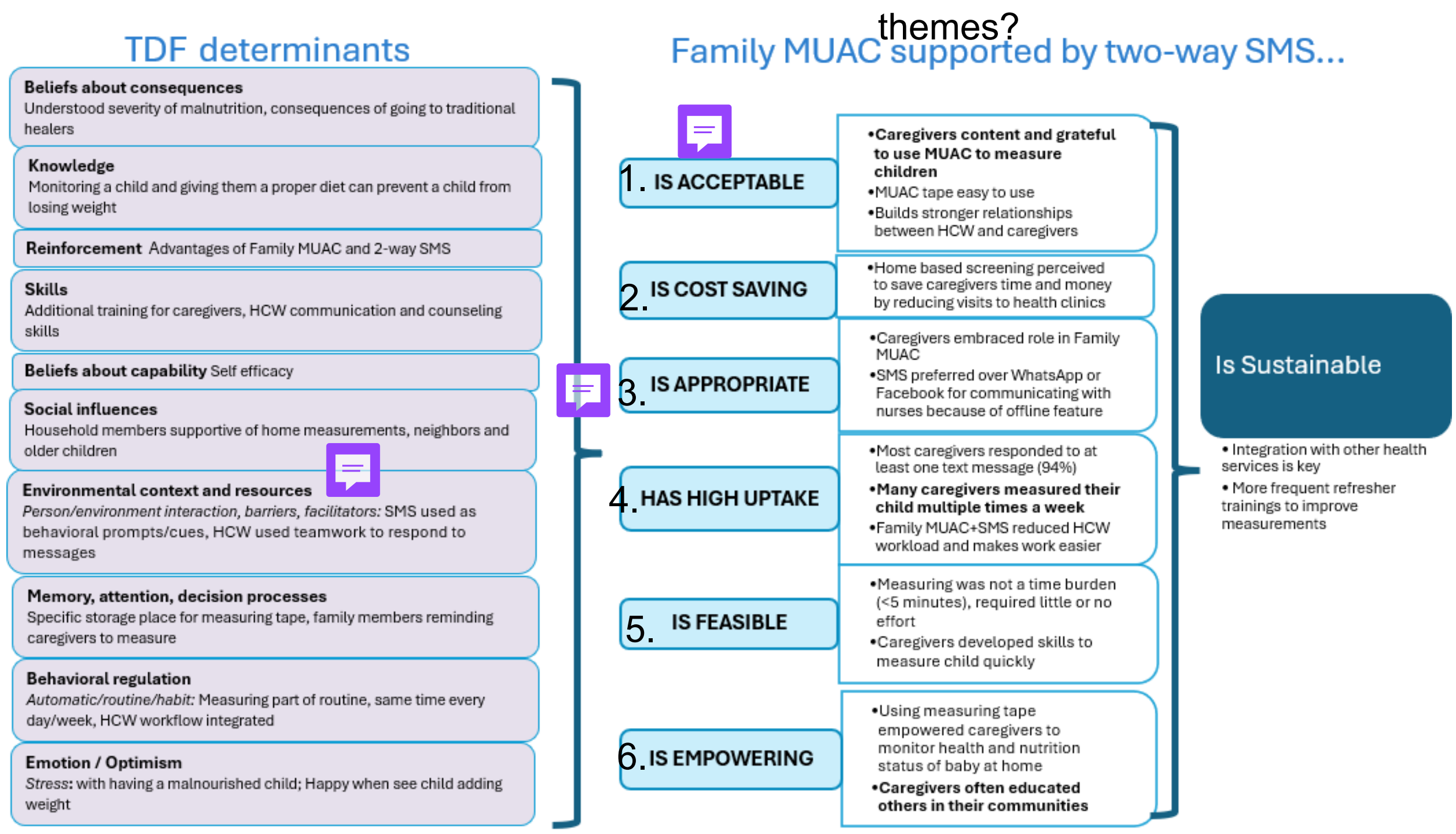

1. Family MUAC had high acceptability among caregivers and HCWs and strengthened the family-clinician relationship

Overall, Family MUAC supported by two-way SMS was perceived as acceptable. Of the caregivers randomized to the intervention group, 99% felt very comfortable or somewhat comfortable using the MUAC tape [7]. Five primary TDF determinants drove the acceptability of the intervention: *beliefs about consequences*, *environmental context and resources*, *reinforcement*, *beliefs about*

capability, and *emotion/optimism*. Most caregivers were content to use MUAC to measure their child and considered the MUAC tape easy to use (TDF ~~construct:~~ *beliefs about capability*). No considerable difference was noted between high/low interactors and caregivers with acutely malnourished or malnourished children. Caregivers and children bonded over the activity of measuring MUAC, with children enjoying getting measured over time. An FGD member mentioned "I can say it was easy because you didn't have to use force to measure [using MUAC tape]. You just sit down relaxed and the child also liked it when he sees it he runs and comes for the measurement, so we really enjoyed the process." (FGD #2)

In addition, connecting directly with a HCWs on a regular basis resulted in stronger relationships between HCWs and caregivers (TDF ~~construct:~~ *environmental context and resources*). Family MUAC with two-way SMS forged stronger bonds between caregivers and HCWs through building trust and improved communication, resulting in greater satisfaction with the patient/provider relationship which is a key to delivery of quality health services.

> "I am very confident in using MAMMS system to engage with the mothers because the system has made the interaction easier, and it has brought us together with the caregivers so that they share their feelings with us, and we also share our experience with them." (Counselor IDI#2)

Caregivers also found HCWs to be friendly, polite, supportive, and trustworthy in their interactions. One respondent remarked "with the nurse who was sending the SMS, we were assisting each other to benefit the health of my child, it was like she has joined me to monitor, she was like a support system for my child" (FGD #2)

Caregivers understood the gravity of having a child identify as malnourished and the negative consequences of consulting traditional healers instead of trained HCWs (TDF ~~construct:~~ *beliefs about consequences*). Caregivers became motivated when they saw children put on weight (TDF ~~construct:~~ *emotion*) and it encouraged them to continue to engage with HCWs via SMS and measure MUAC~~,~~ "It was easy, when I saw my child adding weight I was so motivated and happy, I was measuring and sending the message"(FGD #1).

The educational content delivered via SMS in the intervention group was perceived by caregivers to be useful and important to keep a child healthy, namely the importance of hygiene during breastfeeding and practicing personal hygiene to prevent child illness. Receiving these informational messages reinforced the utility of participating in this study and encouraged caregivers to read their messages (TDF construct: *reinforcement*).

> 'they would even send me some educative messages, whether I am feeding the baby well, how is the baby's health, is my baby sleeping under a bed net. Sometimes when I was spreading the net I would check if it was torn or something. So I found the messages I was being sent very beneficial to me and they continued to encourage me and I felt like I was becoming knowledgeable with information that I didn't have." (High interactor IDI#2)

2. Perceptions about cost savings increased acceptability of Family MUAC

Caregivers found family MUAC supported by two-way SMS to be cost saving, primarily driven by the TDF determinant of *knowledge*. Connecting with a healthcare worker via free SMS messaging to discuss the health and growth of their child reduced the need to visit a health clinic. This may

have reduced costs associated with transportation, food, and time spent bringing a child to the health facility, especially when a child was not malnourished.

> "[Participating in Mama Aweza] relieved us of the burden of transport money, coming to town every day, every day, every day because we were taking measurement every week…at least in that regard they have really relieved us of the transport burden, we are very grateful." (FGD #1)

Both HCWs and caregivers mentioned the benefits of reducing transportation costs through engaging in telehealth consultations via SMS. A counselor mentioned, "one advantage was that it was cost effective for the caregiver, because the caregiver was not coming and going back most of the time for the measurement. They were only supposed to come when the child was identified." (Counselor IDI#1)

High interactors mentioned that they engaged more freely with the HCWs via SMS because it was at no cost to them. On the other hand, low interactors were misinformed about the cost of SMS and found messages difficult to understand, which were barriers to uptake of the intervention (TDF construct: *knowledge*). Low interactors cited challenges with understanding the language of text messages and erroneously believed that messages sent or received were at a cost to caregivers. One low interactor mentioned, "I just knew that [the messages] were not free because I have never seen any message that is free, at least I know they charge 1 shilling." (Low interactor IDI#2)

3. Family MUAC supported by two-way SMS is an appropriate mechanism to connect with caregivers and to relay health messages

Both HCWs and caregivers perceived family MUAC supported by two-way SMS as appropriate in rural and urban Kenyan contexts. Caregivers can take on the role of measuring and monitoring a child's MUAC, even if they encountered some cultural and societal resistance. Caregivers were considered appropriate people to measure their child's MUAC since they were in close proximity to the child throughout the day. Providing a MUAC tape to caregivers allowed them to track their child's growth.

> "For me I feel that mothers should be given the MUAC tape to take home because she is the one who will be measuring the child and she measures the child she will know whether the child is moving to the negative or the positive side. Apart from that a mother can even measure another child not just hers." (FGD #4)

The use of SMS to send and receive measurements was preferred over other platforms, such as WhatsApp or Facebook mainly due to the ability to send SMS without an internet connection. Connecting HCWs with caregivers via SMS messaging was perceived as appropriate and SMS messages were also tailored and relevant to the caregivers' context. The cadence of weekly measurements was generally appropriate though some caregivers measured their children more frequently.

> "You see weekly measurement was better because it was not tedious for the mothers, if we could tell them to measure the MUAC of their children daily it could be cumbersome and tedious but again if we could tell them to measure their children monthly, then it

could be a long period so it was such a good interval for earlier tracking of the nutrition status of the participant." – (Counselor IDI#3)

Some caregivers even preferred to receive daily messages because they found the content of SMS messages useful. One high interactor remarked, "I think [messages] can be sent even daily because it is helpful because maybe you are a person who probably didn't go to school or do not know anything about health…then you learn from that [message] and take action." (High interactor IDI#4)

4. Uptake of the Family MUAC is generally high, but can be compromised by individual and social-level barriers

Families randomized to Family MUAC supported by two-way SMS arm had high uptake of the intervention with 94% responding to one text message or more over the period of enrollment [7]. Caregivers in the intervention sent a median of 20 messages (interquartile range (IQR): 15-24). Four determinants were identified as primarily driving caregiver uptake of Family MUAC supported by two-way text messaging including *environmental context and resources*, *memory/attention/decision processes*, *knowledge*, and *social influences*.

Several factors underlying uptake were identified under the TDF ~~construct~~ *environmental context and resources*. Low interactors mentioned more barriers to uptake compared to high interactors. Barriers for uptake included 1) challenges with taking MUAC measurements, 2) negative social influences, and 3) phone issues and sharing. Barriers and facilitators for uptake of Family MUAC supported by two-way SMS are presented below.

Low interactors expressed frustration when multiple measurements were taken in the same sitting with inconsistent results. This confused caregivers as to what measurement to report to HCWs especially when measurements fell under different colors or numbers indicating that potential retraining is needed to ensure high accuracy and to increase reliability of measurements. A low interactor mentioned “there was a way reading the number was a problem, sometimes I would get 14 sometimes 13.8 and sometimes 12 within the same measuring time so I didn’t know what to report" (Low interactor IDI#3)

In addition, low interactors reported the monotony of sending the same color measurements every week. These caregivers did not understand the advantage of monitoring MUAC measurements if a child was healthy. An example is given here:

> “If I have measured and the child was in green the previous one, so it was green all the time I found that monotonous like there is no difference. So I never wanted to send…it reached a point I was getting tired of sending because of that monotony” (Low interactor IDI#3)

Several high interactors also mentioned the challenges with measuring fussy, restless children especially towards the beginning of the study. In most cases, this was overcome with time as children became accustomed to being measured.

Low interactors perceived greater negative social stigma with measuring children compared to high interactors (TDF construct: *social influences*). Participants noted that many mothers-in-law of low interactors did not support caregivers enrolling into the program or measuring children. In contrast, high interactors received more social support from other mothers, mothers-in-law,

neighbors, husbands, and other children in the household leading to a higher uptake of family MUAC than low interactors. They benefited from a wider social network to consult for advice about their child's health.

> "The father also got interested and joined, yes so when it was Friday, even if he is not there because sometimes he comes home late, he would send the older child his name is [X], he will be like "X go and get the measuring tape so we can measure you and also measure [younger child]. Then I would tell him we have already measured the baby but he would insist he also wanted to measure. So I felt it was something good." (High interactor IDI#1)

A supportive household environment encouraged greater engagement with Family MUAC and provided an external stimulus to measure children on a regular basis. Household members often reminded caregivers to measure their children.

Sharing phones was mentioned as a barrier to uptake of Family MUAC by both high and low interactors. Reading and sending messages were difficult when phones were shared with husbands who had different schedules. Sharing a phone presented an additional hurdle to send in measurements as caregivers needed access to the phone and their child at the same time to measure MUAC and send in measurements. Confidentiality was also a challenge for those with shared phones when HCWs exchanged direct SMS messages with caregivers.

High interactors stored MUAC tape in a specific place, which served to remind caregivers to measure their children (TDF ~~construct~~: *memory, attention, decision processes*). One high interactor (#2) stated, "I was keeping one of the tapes in my bedroom, I had hanged it on the

wall so every week when I wake up I would ask myself whether I had measured or not." This decreased the chance of losing or misplacing the MUAC tape and reduced the time needed to measure the child.

5. Integrating Family MUAC supported by two-way SMS behaviors into routine practice made participation more feasible

Caregivers found Family MUAC supported by two-way SMS feasible to integrate into their routine and developed skills to measure a child quickly. The two TDF determinants influencing this theme include *skills* and *behavioral regulation* (Table 3). High interactors were proactive to set a specific day and time of the week to measure their children. They incorporated child measurements into their weekly routine and anticipated the HCWs' messages. Caregivers with non-acutely malnourished children were also able to develop a habit out of measuring their child compared to caregivers with acutely malnourished children. On the other hand, low interactors perceived taking MUAC measurements as a difficult task. They remembered to measure their children only when they received reminder messages. Generally, low interactors had challenges with incorporating measuring their child into routine practice.

Many caregivers measured their child before receiving the HCW's reminder messages, so they could respond immediately when weekly messages were sent from the study - "I measured the baby in the evening around 6 to 7pm at least before the message comes." - High interactor IDI#1

For high interactors, measuring a child was not a time burden (<5 minutes), requiring little or no effort once they learned strategies to calm a child for measurement (TDF ~~construct:~~ *behavioral*

regulation). High interactors reported developing *skills* to measure their child more than low interactors.

HCWs offered a different perspective on skills development. They mentioned that the initial training offered to caregivers on measuring MUAC was often insufficient to ensure high accuracy and reliability of measurements throughout the six-month study period. They emphasized the importance of building measurement skills through retraining. A HCW expressed:

> "Now training them just once in the clinic and leave them to do the activity for the rest of the six months follow up was not such a good idea…Now in training them, you know the brain of a human being, maybe it did not record well. So, what I think that we left, we would have given them time to be going for refresher training of taking the MUAC at home because most of them when it came to exist of the study, most of them were doing their own things." (Counselor IDI#4)

HCWs recommended offering caregivers refresher training on MUAC measurements especially for caregivers who struggled to send in measurements on a regular basis.

In terms of feasibility of using the two-way SMS system from the HCW perspective, many said it took between 30 mins – 1.5 hours to respond to messages daily depending on message load. For example, Monday morning would be busier because they were required to respond to messages that had accumulated over the weekend. Each day, HCWs would respond to 20-50 messages from caregivers (most of the time close to 30). Each provider established a workflow to read, respond to SMS messages, and access dashboard information that fit their schedules. They checked the dashboard when they entered the office, responding to messages that came in the

late evening of the previous day or early in the morning, at mid-day, and at the end of the work day.

6. Family MUAC empowered caregivers to track their child's nutritional status and became health advocates in their communities

Caregivers equipped with MUAC tape felt empowered to track the health status of their child and share their new knowledge with others in their community. One TDF determinant, *beliefs about capabilities*, primarily influenced this theme. Caregivers recognized the ability of MUAC to track the nutrition status of children at the household and community level when MUAC measurements were taken on a regular basis. The MUAC tape was a tool to track growth and provided information on nutritional status to caregivers and HCW. One caregiver expressed:

> "The benefit of measuring the child's arm using the tape measure … is that it will help me know how my child is doing in terms of nutrition for example if he is in colour yellow, green or red I will know the status of his health and I will know what that means" (FGD#3)

Caregivers felt empowered to share the skills and knowledge gained from the intervention with their neighbors and friends (TDF ~~construct~~: *beliefs about capabilities*). Some caregivers were recognized as leaders in their community because of the new skills they acquired in measuring MUAC, which motivated them to measure other children and advise others within their community.

"When I had the MUAC tape I was using to measure the child, I would see a child somewhere and I'd tell the mother to bring the child so that I measure the arm for her, and if I found the child was in yellow or red I would advise her to urgently take the child to the hospital." (FGD#4)

Women equipped with MUAC tapes started counseling other women who had malnourished children. They used these newly developed skills to educate others on the messages they received through SMS messages but also on the benefits of tracking a child's nutrition status over time. One caregiver in an FGD mentioned, "if I see a child losing weight I will advise the mother to give the child a balanced diet or I simply discuss with her the problem because sometimes [doing that] she might be able to know the problem the child is having, discuss with the mother then give advice because now I know the child should be taken to hospital." (FGD#5)

7. HCWs reported that integrating Family MUAC into other health services and daily routine would increase sustainability

HCWs perceived Family MUAC supported by two-way SMS as sustainable because it reduced their workload, allowed for timely follow up of patients, and stored patient information securely. For example, a nutritionist expressed:

"I would want to use the system [if it was available in the future] because it will make my work easier by reducing the workload, it will help me with following up my clients, the information is safe because the system is having a password, having a password in that only authorized persons are able to access. So, the information of the clients will be confidential." (~~Nutritionist~~ IDI#3)

HCWs suggested that integrating Family MUAC supported by two-way SMS with other health services would lead to greater sustainability. For example, if women visit health centers for HIV/AIDS services, they can benefit from enrolling in family MUAC and receive integrated SMS messages about tracking their child's nutrition. A study counselor (IDI#5) mentioned "a mother coming for a prevention of mother-to-child transmission of HIV (PMTCT) service and also coming for [MAMMS] so we could take the initiative of taking her to the PMTCT to fasten the service and then help us make other follow ups faster." This suggests that family MUAC can be introduced to patients who are already visiting health centers for other services.

Discussion

Study findings demonstrate that Family MUAC supported with two-way SMS is perceived as feasible, acceptable, cost-saving, and appropriate for empowering caregivers to monitor childhood acute malnutrition at the household level. Caregivers used a mHealth tool to facilitate timely communication with HCWs who provided guidance and remote counsel. Key drivers of program uptake included a supportive social environment, phone ownership, and adequate training in MUAC measurement techniques. Conversely, barriers such as phone sharing, lack of social support, and inconsistent training were reported, reflecting challenges similar to those described in other studies [15-18].

The use of the mHealth platform to provide remote support was particularly effective in addressing logistical barriers. The free SMS feature reduced financial and time burdens

associated with clinic visits, enabling caregivers to seek timely advice while minimizing transportation and related costs. HCWs benefited by optimizing their workload, as remote guidance reduced the need for home visits in many cases. Moreover, having direct access to HCWs via SMS increased caregivers' acceptance of the program and fostered improved patient-provider relationships. These results are consistent with existing literature on mHealth interventions, which frequently report improvements in caregiver-HCW communication, adherence to health recommendations, cost savings, and reduced healthcare burdens [19-22].

The intervention also had a notable impact on caregiver empowerment. Caregivers reported increased confidence with monitoring their child's health and became health educators within their communities. Equipped with MUAC tapes and new knowledge and skills, caregivers measured neighborhood children and provided health advice and support to neighbors, suggesting the intervention's potential to indirectly promote health awareness and engagement beyond the immediate beneficiaries. These findings align with previous research highlighting the capacity of community-based health programs to strengthen caregiver confidence and participation in health advocacy [17, 22]. By fostering this sense of empowerment, interventions like Mama Aweza could potentially contribute to broader public health goals, including the promotion of gender equality and community resilience [17].

Concerns regarding the accuracy and consistency of MUAC measurements emerged as a challenge. While many caregivers reported confidence in their ability to perform the measurements, concerns about consistency and accuracy were common. Since training on MUAC use was only provided at study onset, low interactors expressed frustration and confusion resulting from inconsistencies in measurement, which may have contributed to lower levels of

engagement with the program. Ongoing training and regular refresher sessions could help to enhance the reliability of caregiver-collected data. HCWs emphasized the need for continuous guidance to address caregiver questions and ensure accurate measurements. These observations align with prior studies that underscore the necessity of sustained training in community-based health interventions to maintain data integrity and program effectiveness [16, 23, 24].

Technology access was another critical factor influencing program uptake. Caregivers without personal phones often relied on shared devices, which limited their ability to engage fully in the program. This challenge was particularly pronounced when the primary phone owner was away from the caregiver or child. Similar barriers have been identified in other mHealth studies, emphasizing the need to consider technological access when designing scalable interventions [15, 17, 25, 26]. Social support within households further played a key role in caregiver engagement. Caregivers with supportive family members, such as spouses, were more likely to remain active in the program, while those facing negative social influences or lacking support experienced difficulties maintaining participation. Engaging additional family members during program implementation could enhance caregiver involvement and overall program success [18]. Storing MUAC tape in a specific place in the home and integrating measurements into a household routine also improved uptake among caregivers.

This study has limitations. Experiences from HCWs from the three referral hospitals in Kenya may not be representative of other areas in Kenya or globally. Additionally, the reliance on baseline training for caregivers introduced the potential for measurement errors, highlighting the need for ongoing skill reinforcement. Dependence on SMS technology also presents challenges for scalability in areas with limited access to mobile phones or network coverage [25]. In addition,

the study enrolled caregivers who were literate or had access to someone to help them read and send SMS, which may limit generalizability to other contexts with lower literacy rates. Addressing these limitations will be critical for adapting and scaling the intervention to diverse populations and contexts.

In summary, this study provided evidence supporting the Family MUAC with two-way SMS intervention as an acceptable, feasible, and appropriate community-driven approach to reduce child wasting. The intervention empowered caregivers as active participants in health promotion activities. Providing guided support to improve the consistency and accuracy of caregiver-led measurements and identifying and empowering community champions of Family MUAC could promote sustainability of this intervention. These findings align with the Global Action Plan on Child Wasting that emphasizes community engagement, capacity building, and gender empowerment in support of the Sustainable Development Goals.

Acknowledgements

The authors would like to thank caregivers from Migori County Referral and Teaching Hospital, St. Joseph's Mission Hospital, and Homa Bay Teaching and Referral Hospital who participated in in-depth interviews and focus group discussions. This study would not be possible without their support and openness to share their experiences.

References

1. Black RE, Victora CG, Walker SP. Maternal and child undernutrition and overweight in low-income and middle-income countries. Lancet. 2013;mid(ttp://www.ncbi.nlm.nih.gov/pubmed/23746772).

2. UNICEF. The State of the World's Children 2019 [updated 2019]. Available from: https://www.unicef.org/media/106506/file/The%20State%20of%20the%20World%E2%80%99s%20Children%202019.pdf.

3. UNICEF. NutriDash database 2013–2018 2020 [updated 2020]. Available from: https://www.unicefnutridash.org.

4. UNICEF. Integrating Early Detection and Treatment of Child Wasting into Routine Primary Health Care Services: A RESOURCE GUIDE TO SUPPORT NATIONAL PLANNING 2021 [updated 2021]. Available from: https://www.unicef.org/documents/integrating-wasting-treatment.

5. Alé FGB, Phelan KPQ, Issa H. Mothers screening for malnutrition by mid-upper arm circumference is non-inferior to community health workers: results from a large-scale pragmatic trial in rural niger. Arch Public Health. 2016;74(38). doi: 10.1186/s13690-016-0149-5pmid:http://www.ncbi.nlm.nih.gov/pubmed/27602207.

6. Blackwell N, Myatt M, Allafort-Duverger T. Mothers Understand And Can do it (MUAC): a comparison of mothers and community health workers determining mid-upper arm circumference in 103 children aged from 6 months to 5 years. Arch Public Health. 2015;73(26). doi: 10.1186/s13690-015-0074-z.

7. Tickell KD, Achieng C, Masheti M, Anyango M, Ndirangu A, Diakhate MM, et al. Family MUAC supported by a two-way SMS platform for identifying children with wasting: the Mama Aweza randomised controlled trial. Eclinicalmedicine. 2023.

8. Morgan EH, Schoonees A, Sriram U, Faure M, Seguin-Fowler RA. Caregiver involvement in interventions for improving children's dietary intake and physical activity behaviors. Cochrane Database of Systematic Reviews. 2020;(ue 1). doi: 10.1002/14651858.CD012547.pub2.

9. Adeoti IG, Cavallaro FL. Determinants of care-seeking behaviour for fever, acute respiratory infection and diarrhoea among children under five in Nigeria. PLoS One. 2022;15;17(9):e0273901. doi: 10.1371/journal.pone.0273901.

10. Bradley SE, Rosapep L, Shiras T. Where do caregivers take their sick children for care? An analysis of care seeking and equity in 24 USAID priority countries. Global Health: Science and Practice. 2020;30;8(3):518-33.

11. Ivankova NV, Creswell JW, Stick SL. Using mixed-methods sequential explanatory design: From theory to practice. Field methods. 2006;18(1):3-20.

12. Tong A, Sainsbury P, Craig J. Consolidated criteria for reporting qualitative research (COREQ): a 32-item checklist for interviews and focus groups. Int J Qual Health Care. 2007;19(6):349-57. Epub 20070914. doi: 10.1093/intqhc/mzm042. PubMed PMID: 17872937.

13. Tickell KD, Diakhate MM, Goodman JL, Unger JA, Richardson BA, Means AR, et al. Impact of a two-way short message service (SMS) to support maternally administered

childhood mid-upper arm circumference monitoring and expand malnutrition screening in Kenya: the Mama Aweza trial protocol. BMJ open. 2020;1;10(9):e036660.

14. Atkins L, Francis J, Islam R. A guide to using the Theoretical Domains Framework of behaviour change to investigate implementation problems. Implementation Sci. 2017;12:77. doi: 10.1186/s13012-017-0605-9.

15. Addotey-Delove M, Scott RE, Mars M. Healthcare Workers' Perspectives of mHealth Adoption Factors in the Developing World: Scoping Review. International Journal of Environmental Research and Public Health. 2023;20(2). doi: 10.3390/ijerph20021244.

16. Buttarelli E, Woodhead S, Rio D. Family MUAC: A review of evidence and practice. 2020.

17. Obasola OI, Mabawonku I, Lagunju I. A Review of e-Health Interventions for Maternal and Child Health in Sub-Sahara Africa. Matern Child Health J. 2015;19:1813-24. doi: 10.1007/s10995-015-1695-0.

18. Martin SL, McCann JK, Gascoigne E, Allotey D, Fundira D, Dickin KL. Engaging family members in maternal, infant and young child nutrition activities in low- and middle-income countries: A systematic scoping review. Matern Child Nutr. 2021;17:e13158.

19. Mahmood H, McKinstry B, Luz S, Fairhurst K, Nasim S, Hazir T, et al. Community health worker-based mobile health (mHealth) approaches for improving management and caregiver knowledge of common childhood infections: A systematic review. J Glob Health. 2020;Dec;10(2):020438. doi: 10.7189/jogh.10.020438.

20. Charanthimath U. Community health worker evaluation of implementing an mHealth application to support maternal health care in rural India. Frontiers in Global Women's Health. 2021;2:645690.

21. Paduano S, Incerti F, Borsari L, Benski AC, Ernest A, Mwampagatwa I, et al. Use of a mHealth System to Improve Antenatal Care in Low and Lower-Middle Income Countries: Report on Patients and Healthcare Workers' Acceptability in Tanzania. International Journal of Environmental Research and Public Health. 2022;19(22). doi: 10.3390/ijerph192215342.

22. Early J, Gonzalez C, Gordon-Dseagu V, Robles-Calderon L. Use of Mobile Health (mHealth) Technologies and Interventions Among Community Health Workers Globally: A Scoping Review. Health Promotion Practice. 2019;20(6):805-17. doi: 10.1177/1524839919855391.

23. Saida RH, Masinde L, Mugambi L. Assessing Factors Hindering Effectiveness of Family MUAC Program on Nutrition Surveillance and Early Detection of Malnutrition Cases Among Children Aged 6-59 Months in Banisa Sub County. Journal of Medicine, Nursing & Public Health. 2024;7(1):117-26.

24. Karamolahi PF, Khalesi ZB, Niknami M. Efficacy of mobile app-based training on health literacy among pregnant women: A randomized controlled trial study. European Journal of Obstetrics & Gynecology and Reproductive Biology. 2021;X 12:100133.

25. Allsop MJ, Namisango E, Powell RA. A survey of mobile phone use in the provision of palliative care services in the African region and priorities for future development. Journal of telemedicine and telecare. 2019;25(4):230-40.

26. Chang LW. Impact of a mHealth intervention for peer health workers on AIDS care in rural Uganda: a mixed methods evaluation of a cluster-randomized trial. AIDS and Behavior. 2011;15:1776-84.

Click here to access/download
Supporting Information
COREQ checklist MamaAweza.docx

UNIVERSITY of WASHINGTON
HUMAN SUBJECTS DIVISION

IRB CONTINUING REVIEW APPROVAL

November 24, 2021

Dear Christine McGrath:

On 11/24/2021, University of Washington IRB Committee J reviewed the following application:

Type of Review:	Continuing Review
Title of Study:	Developing Low-Cost Universal Malnutrition Screening for Low Income Countries – the MAMMS Trial
Investigator:	Christine McGrath
STUDY ID	STUDY00006221
CR ID:	CR00005261
Funding:	Name: Thrasher Research Fund, Grant Office ID: A132680, Funding Source ID: N/A
IND, IDE, or HDE:	None

IRB Approval

Under FWA #00006878, the IRB renewed approval for your activity from 11/24/2021 to 11/23/2022.

- COVID NOTE: See the HSD website for the latest COVID guidelines for conducting human subjects research.
- Your application qualified for expedited review ("minimal risk"; Category 4, 5, 6, & 7).

Thank you for your commitment to ethical and responsible research. We wish you great success!

Sincerely,

Theresa Naluai-Cecchini
IRB Administrator, Committee J
206.543.3494
tnaluai@uw.edu
hsdteamj@uw.edu

KENYA MEDICAL RESEARCH INSTITUTE

P.O. Box 54840-00200, NAIROBI, Kenya
Tel: (254) 2722541, 2713349, 0722-205901,0733-400003, Fax: (254) (020) 2720030
Email: director@kemri.org, info@kemri.org, Website. www.kemri.org

KEMRI/RES/7/3/1 **April 29, 2021**

TO: **DR. BENSON SINGA,**
PRINCIPAL INVESTIGATOR.

THROUGH: **THE DEPUTY DIRECTOR, CCR,**
NAIROBI.

Dear Sir,

RE: **SERU 3821 *(REQUEST FOR ANNUAL RENEWAL)*: DEVELOPING LOW-COST UNIVERSAL MALNUTRITION SCREENING FOR LOW INCOME COUNTRIES – THE MAMMS TRIAL**

Thank you for the continuing review report for the period **May 3, 2020 to March 18, 2021.**

This is to inform you that the expedited review team of the KEMRI Scientific and Ethics Review Unit (SERU) conducted the annual review of the above referenced application and was of the informed opinion that the progress made during the reported period is satisfactory. The study has therefore been granted **approval**.

This approval is valid from **May 03, 2021** for a period of **one (1) year**. Please note that authorization to conduct this study will automatically expire on **May 02, 2022.** If you plan to continue with data collection or analysis beyond this date, please submit an application for continuation approval to SERU by **March 21, 2022**.

You are required to submit any amendments to this protocol and any other information pertinent to human participation in this study to the SERU for review prior to initiation.

You may continue with the study.

Yours faithfully,

ENOCK KEBENEI,
THE ACTING HEAD,
KEMRI SCIENTIFIC AND ETHICS REVIEW UNIT.

CONFIDENTIAL

KENYA MEDICAL RESEARCH INSTITUTE

P.O. Box 54840-00200, NAIROBI, Kenya
Tel: (254) 2722541, 2713349, 0722-205901, 0733-400003, Fax: (254) (020) 2720030
Email: director@kemri.org, info@kemri.org, Website. www.kemri.org

KEMRI/RES/7/3/1 **April 7, 2022**

TO: **DR. BENSON SINGA,**
PRINCIPAL INVESTIGATOR.

THROUGH: **THE DEPUTY DIRECTOR, CCR,**
NAIROBI.

Dear Sir,

RE: **SERU 3821 *(REQUEST FOR ANNUAL RENEWAL)*: DEVELOPING LOW-COST UNIVERSAL MALNUTRITION SCREENING FOR LOW INCOME COUNTRIES – THE MAMMS TRIAL**

Thank you for the continuing review report for the period **03 May 2021 to 17 March 2022.**

This is to inform you that the expedited review team of the KEMRI Scientific and Ethics Review Unit (SERU) conducted the annual review of the above referenced application and was of the informed opinion that the progress made during the reported period is satisfactory. The study has therefore been granted **approval**.

This approval is valid from **May 03, 2022** for a period of **one (1) year**. Please note that authorization to conduct this study will automatically expire on **May 02, 2023.** If you plan to continue with data collection or analysis beyond this date, please submit an application for continuation approval to SERU by **March 21, 2023**.

You are required to submit any amendments to this protocol and any other information pertinent to human participation in this study to the SERU for review prior to initiation.
You may continue with the study.

Yours faithfully,

PROF. CHARLES OBONYO,
THE ACTING HEAD,
KEMRI SCIENTIFIC AND ETHICS REVIEW UNIT.

UNIVERSITY of WASHINGTON
HUMAN SUBJECTS DIVISION

IRB APPROVAL OF APPLICATION

December 5, 2018

Dear Christine McGrath:

On 12/5/2018, University of Washington IRB Committee J reviewed the following application:

Type of Review:	Initial Study
Title of Study:	Developing Low-Cost Universal Malnutrition Screening for Low Income Countries – the MAMMS Trial
Investigator:	Christine McGrath
IRB ID:	STUDY00006221
Funding:	Name: Thrasher Research Fund, Grant Office ID: A132680, Funding Source ID: N/A
IND, IDE, or HDE:	None

IRB Approval

Under FWA #00006878, the IRB approved your activity from 12/5/2018 to 12/4/2021.

- **Depending on the nature of your study, you may need to obtain other approvals or permissions to conduct your research. For example, you might need to apply for access to data or specimens (e.g., to obtain UW student data). Or, you might need to obtain permission from facilities managers to approach possible subjects or conduct research procedures in the facilities (e.g., Seattle School District; the Harborview Emergency Department).**
- Your application qualified for expedited review ("minimal risk"; Category 5).
- This 3-year approval period applies only to research that is not federally-supported. If you obtain federal support during this time, you must immediately submit a Continuing Review and a Modification with the new funding information.
- This approval applies only to the activities described in your application (including any references to specific grant sections). It does not include other activities that may be described in your grant or contract.
- This approval applies only to accessing and obtaining of identifiable data. It does not apply to all other activities conducted by the local site.

Thank you for your commitment to ethical and responsible research. We wish you great success!

Sincerely,

Lindsey Westlake
Review Administrator
206-897-1748
scaggl@uw.edu

4333 Brooklyn Ave. NE, Box 359470 Seattle, WA 98195-9470
main 206.543.0098 fax 206.543.9218 hsdinfo@u.washington.edu www.washington.edu/research/hsd

KENYA MEDICAL RESEARCH INSTITUTE

P.O. Box 54840-00200, NAIROBI, Kenya
Tel:(254) (020) 2722541, 2713349, 0722-205901, 0733-400003, Fax: (254) (020) 2720030
E-mail: director@kemri.org, info@kemri.org, Website.www.kemri.org

KEMRI/RES/7/3/1 **May 3, 2019**

TO: **DR. BENSON SINGA,**
PRINCIPAL INVESTIGATOR

THROUGH: **THE DIRECTOR, CCR,**
NAIROBI

8/May/2019

Dear Sir,

RE: **KEMRI/SERU/CCR/0121/3821 *(RESUBMITTED INITIAL)*: DEVELOPING LOW-COST UNIVERSAL MALNUTRITION SCREENING FOR LOW INCOME COUNTRIES-The MAMMS TRIAL**

Reference is made to your letter dated March 13, 2019. The KEMRI Scientific and Ethics Review Unit (SERU) acknowledges receipt of the following revised study documents on March 21, 2019:

1. Responses to reviewers' comments version 13Mar2019
2. Revised Protocol version 1.2 dated 13 March 2019.
3. Dated and signed CVs for Non-KEMRI Investigators
4. Alima Training materials (Appendix E)
5. FGD and IDI tools_05Mar2019

This is to inform you that the Committee noted that the issues raised during the 284th Committee B meeting of the KEMRI Scientific Ethics Review Unit (SERU) held on **February 20, 2019** have been adequately addressed.

Consequently, the study is **granted approval** for implementation effective this day, **May 03, 2019** for a period of one year. Please note that authorization to conduct this study will automatically expire on **May 02, 2020**. If you plan to continue data collection or analysis beyond this date, please submit an application for continuation approval by **March 21, 2020.**

You are required to submit any proposed changes to this study to the SERU for review and the changes should not be initiated until a written approval from the SERU is received. Please note that any unanticipated problems resulting from the implementation of this study should be brought to the attention of SERU and you should advice the SERU when the study is completed or discontinued.

You may embark on the study.

Yours faithfully,

RUKithinji

ENOCK KEBENEI,
for. **THE ACTING HEAD,**
KEMRI SCIENTIFIC AND ETHICS REVIEW UNIT.
