# Supplementary material for: Acceptability, feasibility, appropriateness, uptake and cost perceptions of family mid-upper arm circumference supported by two-way SMS in western Kenya
Source: PLoS One. 2026 Sep 23;21(9):e0358775. doi: 10.1371/journal.pone.0358775 (PMC13600513; doi:10.1371/journal.pone.0358775)
Supplement: Supplementary file 3 [file pone.0358775.s006.pdf]

# KENYA MEDICAL RESEARCH INSTITUTE

P.O. Box 54840-00200, NAIROBI, Kenya
Tel:(254) (020) 2722541, 2713349, 0722-205901, 0733-400003, Fax: (254) (020) 2720030
E-mail: director@kemri.org, info@kemri.org, Website.www.kemri.org


**KEMRI/RES/7/3/1** **May 3, 2019**

**TO:** **DR. BENSON SINGA,**
**PRINCIPAL INVESTIGATOR**

**THROUGH:** **THE DIRECTOR, CCR,**
**NAIROBI**

8/5/2019

Dear Sir,

**RE:** **KEMRI/SERU/CCR/0121/3821 *(RESUBMITTED INITIAL)*: DEVELOPING LOW-COST UNIVERSAL MALNUTRITION SCREENING FOR LOW INCOME COUNTRIES-The MAMMS TRIAL**

Reference is made to your letter dated March 13, 2019. The KEMRI Scientific and Ethics Review Unit (SERU) acknowledges receipt of the following revised study documents on March 21, 2019:

1. Responses to reviewers' comments version 13Mar2019
2. Revised Protocol version 1.2 dated 13 March 2019.
3. Dated and signed CVs for Non-KEMRI Investigators
4. Alima Training materials (Appendix E)
5. FGD and IDI tools_05Mar2019

This is to inform you that the Committee noted that the issues raised during the 284th Committee B meeting of the KEMRI Scientific Ethics Review Unit (SERU) held on **February 20, 2019** have been adequately addressed.

Consequently, the study is **granted approval** for implementation effective this day, **May 03, 2019** for a period of one year. Please note that authorization to conduct this study will automatically expire on **May 02, 2020**. If you plan to continue data collection or analysis beyond this date, please submit an application for continuation approval by **March 21, 2020.**

You are required to submit any proposed changes to this study to the SERU for review and the changes should not be initiated until a written approval from the SERU is received. Please note that any unanticipated problems resulting from the implementation of this study should be brought to the attention of SERU and you should advice the SERU when the study is completed or discontinued.

You may embark on the study.

Yours faithfully,

RCTKithinji

for: **ENOCK KEBENEI,**
**THE ACTING HEAD,**
**KEMRI SCIENTIFIC AND ETHICS REVIEW UNIT.**
