# Supplementary material for: Acceptability, feasibility, appropriateness, uptake and cost perceptions of family mid-upper arm circumference supported by two-way SMS in western Kenya
Source: PLoS One. 2026 Sep 23;21(9):e0358775. doi: 10.1371/journal.pone.0358775 (PMC13600513; doi:10.1371/journal.pone.0358775)
Supplement: Supplementary file 6 [file pone.0358775.s007.docx]

Dear Editor,

Thank you for sharing additional reviewer comments on our manuscript, “Acceptability, feasibility, appropriateness, uptake and cost perceptions of Family Mid-Upper Arm Circumference supported by two-way SMS in western Kenya”. Please find our responses to each comment below:

Reviewer #8: Comment 1
The study relies exclusively on self-reported perceptions of cost savings rather than a formal economic evaluation. While caregivers expressed that the intervention reduced transportation expenses, no cost-effectiveness or cost-benefit analysis was conducted to substantiate these claims. This limits the ability to draw robust conclusions about the actual financial impact of the intervention.

Response: Thank you for this comment. We acknowledge that a formal economic evaluation was not conducted and acknowledge this limitation in the discussion section on page 30:

*“we collected perceptions about cost savings from caregivers but do not report on a full economic evaluation to quantify these perceived cost savings. A cost-effectiveness analysis or cost-benefit analysis could provide further evidence of cost savings.”*

Comment 2
The definition of high and low engagement was based solely on response rates to SMS messages (above 90% versus below 30%). However, response frequency does not necessarily reflect meaningful engagement, understanding, or correct performance of MUAC measurements. A more nuanced composite measure of engagement would have strengthened the analysis.

Response: Thank you for this thoughtful suggestion. We were interested in capturing engagement with the two-way SMS system between caregivers and healthcare workers. According to the data collected from program participants, this was captured through response frequency. You are correct that a more nuanced definition of engagement could have been applied, but we did not aim to capture accuracy of measurements as part of engagement. Instead, our engagement measure sought to capture caregivers’ sustained and consistent sharing of MUAC measurements via the two-way SMS system over the course of the study period.

Comment 3
The purposive sampling strategy for interviews was clearly described, yet the absence of detailed sociodemographic data (e.g., literacy level, household wealth, maternal education) prevents an assessment of whether specific subgroups differentially benefited or struggled with the intervention. This omission weakens the ability to comment on equity implications.

Response: Thank you for this suggestion. We did not attempt to comment on equity implications due to our inability to present sociodemographic data on sampled participants. We acknowledge this as a limitation in our study. On page 29, we indicate:

*“We did not collect detailed socio-demographic characteristics of participants, which limits our ability to assess how factors such as literacy, socioeconomic status, or access to broader household resources may have influenced engagement with the intervention. Although we purposively sampled participants across sites and levels of engagement, the absence of these data restricts our ability to examine potential differences between groups.”*

Comment 4
The study acknowledges that caregivers needed to be literate or have access to someone who could read and send SMS messages. This represents a substantial selection bias, and the findings may not be transferable to settings with lower literacy rates or less access to mobile phones. The manuscript would benefit from a more explicit discussion of how this limits generalisability.

Response: Interestingly, no caregivers were excluded from the Mama Aweza study because they were unable to read or write or did not have someone who knew how to read or write. Reportedly, caregivers were not excluded because they did not have mobile phones but only that their phones did not use the compatible service provider (Safaricom; the most common provider in the region). Nonetheless, you are correct that this is a limitation of the intervention, and we acknowledge that this intervention may perform less well in areas of low literacy. We thank you for your comment and consider it pertinent, so we have added to our limitations section on page 30:

*“In addition, the study enrolled caregivers who were literate or had access to someone to help them read and send SMS, which may limit transferability to other contexts with lower literacy rates or less access to mobile phones.”*

Comment 5
Concerns about measurement accuracy were raised primarily by low-interactor caregivers and health workers, yet the study did not independently verify caregiver-performed MUAC measurements against a gold standard. Without objective validation, it remains unclear whether the reported reductions in wasting were attributable to accurate home monitoring or to other factors.

Response: To clarify, when a caregiver screened a child as wasted (red or yellow colored section of the MUAC tape) at home, caregivers were asked to bring the child to the health facility where a trained health professional confirmed the MUAC measurement. All caregiver-performed MUAC measurements identified as moderately or severely wasted were objectively verified by a study staff/trained health professional. If the caregiver reported yellow/red and it was verified by the trained staff to not be red/yellow, the caregiver received refresher MUAC training. Additional details have been added on page 5:

*“Caregiver-performed MUAC measurements identified as moderate or severely wasted were verified by a trained health professional.”*

Additionally*: “children diagnosed with wasting, as confirmed by a health professional at any visit in both arms, were provided treatment according to Kenya’s National Guidelines, with their progress monitored through scheduled follow-up visits.”*

Comment 6
The Theoretical Domains Framework was appropriately applied to guide data collection and analysis. However, the presentation of results would be strengthened by a more systematic mapping of specific TDF constructs to individual themes, rather than the relatively high-level grouping currently provided.

Response: Thank you, we identified the specific constructs that stood out as outlined in Figure 1 and in the additional tables added to the manuscript. For example, on Table 2 we highlighted the construct of person x environment interaction under the TDF domain environmental context and resources and provided illustrative quotes to support this finding. On Figure 1 we specified the construct Automatic/routine/habit under the domain of behavioral regulation.

Comment 7
The finding that some caregivers mistakenly believed SMS messages incurred a cost, which reduced engagement, is important. Yet the manuscript does not report whether any efforts were made during the trial to correct this misunderstanding proactively. This represents a missed opportunity to improve intervention design.

Response: Your observation has been noted. These qualitative interviews and focus groups were conducted after the trial concluded and therefore we were unable to provide feedback during trial implementation to correct these misunderstandings. These findings can be helpful to help improve the design of other mobile SMS interventions.

Comment 8
Health workers reported spending between 30 minutes and 1.5 hours daily responding to messages, with peaks of up to 50 messages per day. The feasibility of scaling this intervention to routine health system settings, where staff already face heavy workloads, was not adequately explored. This raises concerns about sustainability outside of a research context.

Response: We agree that the additional workload associated with responding to caregiver messages is an important consideration for the feasibility and sustainability of scaling this intervention within routine health system settings. Although health care workers in our study were able to establish workflows that allowed them to read and respond to messages within their schedules and generally perceived the intervention as feasible to integrate into their workstreams, our findings also indicate that the time and staffing demands associated with providing ongoing two-way SMS support would need to be carefully considered before scale-up. We have highlighted this consideration in the Discussion, noting that appropriate staffing, task-shifting strategies, or automation of routine responses may be important for supporting the feasibility and sustainability of this approach at scale (page 27)*:*

*“providing ongoing support through two-way SMS platforms may introduce additional demands on HCWs, particularly in terms of time required to review and respond to caregiver messages. Without careful program design, including appropriate staffing, task-shifting strategies, or automation of routine responses, these added responsibilities could increase workload and potentially limit the feasibility and scalability of such interventions within already resource-constrained health systems.”*

Comment 9
Social support from family members, particularly husbands and children, emerged as a strong facilitator of engagement, while resistance from mothers-in-law was a barrier. The study does not propose specific strategies to address negative social influences or to engage resistant family members in future implementations.

Response: Thank you for this timely comment. We agree that engaging mothers-in-law and fathers could improve adoption of this intervention. There are various ways to engage resistant family members within child nutrition programs. We have added additional insights based on your comment on pages 28-29:

*“Evidence from maternal and child nutrition programs suggests that engaging additional family members during program implementation, including male partners and senior female relatives, could enhance caregiver involvement and overall program success [21]. Approaches such as grandmother or husband support groups, digital health interventions that engage non-primary caregiver household members, and facility-based interventions such as couples counseling sessions during routine health visits could increase involvement and engagement in child nutrition programs [21].”*

Comment 10
The intervention's potential to empower caregivers and transform them into community health advocates is compelling. However, the data supporting this claim are largely anecdotal, consisting of a few illustrative quotes. No systematic attempt was made to measure the extent or impact of this community-level diffusion of knowledge and skills.

Response: This is correct, it was not within our scope to measure the extent of community-level diffusion of empowering caregivers as health advocates. Since the interviews and focus groups only involved caregivers who participated in the trial, we did not aim to measure community member perceptions of caregivers involved in the intervention. This should not undermine the finding that caregivers felt increased agency due to their participation in the Family MUAC + SMS intervention and the internal motivation they felt to measure their children, and children in their neighborhoods.

Comment 11
Phone sharing was identified as a significant barrier to uptake, yet the manuscript does not discuss whether alternative communication channels (e.g., voice calls, community health worker visits, or group messaging) were considered or tested for caregivers without private phone access. This limits the practical relevance of the findings for the most disadvantaged populations.

Response: Thank you for this insightful comment. As SMS was a central component of our intervention (along with participation in Family MUAC), we targeted women who had any access to a phone – either individually owned or a shared phone. The two-way SMS approach was being evaluated as a potentially more scalable and less resource-intensive alternative to the routine CHW visits that are part of the standard of care in Kenya. Our findings highlight phone sharing as an important barrier to equitable participation in digital health interventions. While testing alternative communication channels or providing phones was beyond the scope of this study, future research should examine strategies such as phone provision or alternative communication modalities to address this barrier and support equitable scale-up.

Comment 12
The conclusion states that the intervention is "acceptable, feasible, and appropriate" based on qualitative data from a single region in western Kenya. While these findings are valuable for hypothesis generation, the lack of quantitative generalizability data or a formal implementation outcomes framework means the conclusions should be framed more cautiously, acknowledging the context-specific nature of the results.

Response: We appreciate your comment. We applied the Theoretical Domains Framework, an implementation science framework to structure data analysis and identify themes in a systematic way. Applying an implementation science framework is a common way to rigorously assess the implementation outcomes of acceptability, feasibility, appropriateness, perceived cost of a program or intervention. On the note about the “generalizability” of the data, we have added that the findings apply to the western Kenyan context on page 25 and we noted that the transferability of our study findings to contexts with different characteristics may be limited on page 30. However, consistent with qualitative research principles, the goal is not to produce universally generalizable results, but rather to explore topics in depth and provide rich, context-specific insights that may support transferability to similar settings.

Reviewer #9: Introduction:
Page 3 Line 43: It seems that the present study is only qualitative and not a mixed methods one. No quantitative data is provided.

Response: Thank you for this insightful comment. We used quantitative cut-offs to define the sampling frame for engagement, but we do acknowledge that most data and analysis presented are qualitative. Our quantitative data analyses were minimal and served to set up the qualitative analyses. Examples of the quantitative data we analyzed can be found on page 17:

*“Families in the intervention arm had high uptake of the intervention with 94% responding to one text message or more over the period of enrollment [1]. Caregivers in the intervention sent a median of 20 messages (interquartile range (IQR): 15-24) over the six-month enrollment period.”*

However, due to the limited nature of our quantitative analysis compared to qualitative data and results, we have revised the study design to reflect a qualitative study and removed the study’s description as mixed methods as reflected in the Abstract on page 2,

*“This qualitative study involved eight focus group discussions with caregivers enrolled in the Mama Aweza trial and fourteen in-depth interviews with caregivers who participated in the intervention arm”*

*Additionally, on page 3, “We employed a qualitative design to investigate the determinants of engagement with Family MUAC supported by two-way SMS intervention for purposes of development and expansion. We used qualitative data to identify and compare responses between caregivers whose children did and did not become acutely malnourished during the study.”*

Methods:
How was validity and reliability of qualitative data gathered determined?

Response: Thank you for this comment. To enhance the rigor and trustworthiness of the qualitative data and analysis, all IDIs and FGDs were audio-recorded, transcribed, and translated into English. To ensure transcription quality, investigators who did not conduct the original transcriptions performed random spot checks for each transcript by comparing the audio recordings with the transcripts. Any discrepancies prompted a full review of the transcript. Additionally, two primary coders independently coded the first two transcripts using the a priori TDF-based codebook and inductively identified new codes. Emerging codes and differences in code application were discussed in consensus meetings involving additional members of the research team, and a third researcher acted as a tiebreaker when consensus could not be reached. Code definitions and inclusion and exclusion criteria were refined throughout this iterative process. Finally, the final themes and conceptual framework were discussed among the coders and research team to ensure that they reflected the patterns identified across the FGDs and IDIs.

Results:
By presenting the number of each group in the text, Table 1 can be removed:
“(1) caregivers in the intervention group whose child did not develop acute malnutrition (n=12), (2) caregivers in the intervention group whose child became acutely malnourished (n=13), (3) SOC participants whose child did not develop acute malnutrition (n=13), and (4) SOC participants whose child became acutely malnourished over the duration of the study (n=13).”

Response: Thank you, this has been modified as suggested, and Table 1 was removed.

Despite the claim of authors for using mixed method, only the results of qualitative study have been presented.
Although tables have been added to the text of qualitative findings at the suggestion of the previous reviewers, the way of presenting the findings is not appropriate and it is better to present qualitative tables containing themes and sub-themes.

Response: We have taken into consideration your feedback and revised the study design to a qualitative study. Additionally, Figure 1 depicts themes and sub-themes for this study through a schematic. Tables 2-5 included at the request of previous reviewers present additional data and constructs organized by theme. While Figure 1 is the overarching summary of themes and constructs, the additional tables provide more nuanced insights under each theme.

Discussion:
The following related article is not referenced:
Abdissa, H.G.; Duguma, G.B.; Gerbaba, M.; Noll, J.; Sori, D.A.; Koricha, Z.B. Acceptability, Feasibility, and Appropriateness of Mobile Phone Messaging-Based Message-Framing Intervention for Promoting Maternal and Newborn Care Practices. Int. J. Environ. Res. Public Health 2025, 22, 864. [https://doi.org/10.3390/ijerph22060864](https://urldefense.com/v3/__https:/doi.org/10.3390/ijerph22060864__;!!K-Hz7m0Vt54!n4i5oeWx3qcg5tHpUfHDpF0SoXMwzA3it_RZu0d-avrzhubljO4hnNtCOYW-c0aAGe-mr35jXaftr04$).

Response: Thank you for this suggestion. We cited this reference to support our insights on phone sharing as a barrier in mHealth interventions.

Reviewer #10: In review of the article, the authors have taken into account the vast majority of previous peer review comments and made the requested improvements where noted in the article. In particular, the explanations in the methods section, the more detailed presentation of participant characteristics, the expansion of limitations, and the clarification of various conceptual points have increased the publishability of the study.

However, although the Discussion section has been supplemented with additional sources as mentioned in the previous revision, the improvement remains limited, and a more detailed discussion of the similarities and differences between the findings and previous studies, particularly regarding caregiver involvement, measurement accuracy, social support mechanisms, and the sustainability of the intervention, would be better.

Response: Thank you for this comment and for highlighting these gaps in our discussion. We added more to the discussion to reflect on the similarities and differences between our study findings and previous studies. In particular, differences with other studies involving caregiver involvement and social support mechanisms are now highlighted on page 28,

“*Evidence from maternal and child nutrition programs suggests that engaging additional family members during program implementation, including male partners and senior female relatives, could enhance caregiver involvement and overall program success [22]. Approaches such as grandmother or husband support groups, digital health interventions that engage non-primary caregiver household members, and facility-based interventions such as couples counseling sessions during routine health visits could increase involvement and engagement in child nutrition programs [22].”*

On page 28, “*HCWs emphasized the need for continuous guidance to address caregiver questions and ensure accurate measurements. Prior studies have also underscored the necessity of sustained training in community-based health interventions to maintain data integrity and program effectiveness, and to improve sustainability.”*

Additionally, we demonstrate similarities between our study and previous studies on page 27, “*Caregivers reported increased confidence with monitoring their child’s health and became health educators within their communities, a finding supported by previous studies in South Africa and Niger using a Family MUAC approach.”*

Also, in response to the previous revision, while tables regarding the number of FGD participants have been added, reporting the number of participants in each focus group interview separately would be a more transparent approach.

Response: Thank you for suggesting this clarification. We have added on page 9, “We conducted eight FGDs (51 total FGD participants with group size ranging from 6-7 participants)” to clarify the number of participants in each group.

Furthermore, the lack of sociodemographic data for participants is a significant limitation of the study. Therefore, it was not possible to assess potential differences between high and low-interaction participants in terms of age, education level, socioeconomic status, or other individual characteristics. This partially limits the interpretation and transferability of the findings.

Response: We acknowledge this as a limitation of the study. We did not collect additional demographic information from caregivers in the qualitative sample, in part to protect confidentiality. We have outlined the lack of reporting on demographic characteristics for interview participants in the discussion, on page 29:

*“We did not collect detailed socio-demographic characteristics of participants, which limits our ability to assess how factors such as literacy, socioeconomic status, or access to broader household resources may have influenced engagement with the intervention. Although we purposively sampled participants across sites and levels of engagement, the absence of these data restricts our ability to examine potential differences between groups.”*

Though we did collect the same number of participants from each facility to ensure all sites had equal representation.

Overall, the revisions made are satisfactory, and the remaining issues are minor improvements. Therefore, I believe the article can be published after minor revisions.

Reviewer #11: The methodology section presented in " PONE-D-25-63080_R1.docx" is highly rigorous, well-structured, and directly aligned with your study’s objectives. The choice of an explanatory sequential mixed-methods design (quan → QUAL) is highly appropriate for contextualising the quantitative trial results through the lived experiences of the caregivers.
Furthermore, your precise, metrics-driven purposive sampling strategy which uses stratifying by clinical outcomes and utilizing strict mathematical thresholds for high and low platform engagement significantly strengthens the comparative validity of your findings. The systematic application of the Theoretical Domains Framework (TDF) combined with a robust dual-coder analysis framework ensures excellent theoretical and analytical depth.

two minor comments:
1. Page 20, title: it would be better if you avoid using the acronym SMS in the title to improve readability.

Response: Thank you for this suggestion. We have considered this recommendation; however, we have retained the acronym “SMS” in the title because it is a widely recognized and commonly used term in the digital health literature. We believe that retaining “SMS” improves conciseness without compromising readability.

2. As a minor formatting note for the final layout, please ensure that the title and caption for Figure 1 are placed directly below the figure, as is customarily done in academic writing.

Response: Thank you for flagging this for editors to consider when typesetting the document.

Reviewer #12: The authors have responded comprehensively to the reviewers' comments and have addressed the majority of the concerns raised during peer review. Clarifications regarding participant eligibility, MUAC color classifications, study procedures, intervention implementation, limitations, and contextualization of the findings have substantially improved the manuscript. However, I still have two minor concerns.

First, the relationship between the previously published RCT and the current qualitative study remains somewhat unclear. I think some clarity is required in study design. I believe the manuscript is essentially a qualitative follow-up study informed by trial data. I want to know the qualitative component was planned from the outset as part of a mixed-methods design, or the qualitative study was conducted later as a separate follow-up study. Because the relationship between the current qualitative study and the previously published RCT is not immediately clear from the abstract unless a reader is familiar with the earlier publication.

The manuscript would benefit from greater clarity regarding the overall study design specially for readers not familiar with previous publication if authors can please clarify whether the study was originally conceptualized and conducted as a mixed-methods study (with both quantitative and qualitative components planned a priori) or whether the qualitative work was undertaken subsequently?

Response: Both the quantitative and quantitative components were planned a priori as outlined in the trial registration ([clinicaltrials.gov](https://clinicaltrials.gov/): [NCT03967015](https://clinicaltrials.gov/show/NCT03967015)) and published protocol paper.^[1]^ This qualitative component was planned from the outset, at the design stage for the Mama Aweza trial. The research team leveraged caregivers enrolled in the parent trial to purposively sample participants and to identify and interview caregivers with high and low engagement. We confirm that the qualitative study informed by trial data was pre-planned before the Mama Aweza trial was carried out. We also revised the abstract and methods sections to reflect the qualitative design of this present study.

Second, while the authors have acknowledged the absence of socio-demographic information as a limitation, factors such as literacy, education, phone ownership, and socioeconomic status appear highly relevant to understanding differences between high and low interactors in an SMS-based intervention. Consideration of these characteristics would have strengthened interpretation of the findings and identification of barriers to engagement.

Response: Thank you for highlighting this consideration. We acknowledge this is a significant limitation of this study and have expanded the discussion to capture this constraint more fully. Although the qualitative interviews identified important differences in barriers and facilitators of engagement between high and low interactors, including challenges related to phone ownership and phone sharing, we were unable to systematically assess whether differences in characteristics such as literacy, education, socioeconomic status, and other household resources contributed to differences in engagement. We have clarified that the absence of these data limits our ability to interpret differences between engagement groups and identify potential sociodemographic barriers to participation.

We have included additions on page 30,

“*Although we purposively sampled participants across sites and levels of engagement, the absence of these data restricts our ability to examine potential differences between groups. Consideration of these characteristics could provide further insights into barriers to engagement.”*

I also have a suggestion regarding title and abstract: Authors should mention study design in the abstract
Abstract word count is 315. I suppose it shoudnt exceed 300? The abstract doesn’t reflect it is a mixed method clearly!

Response: We added that the study is a qualitative study in the abstract and reduced the Abstract to under 300 words.

The title may benefit from replacing Family MUAC with "family MUAC screening" in the study title like “Acceptability, feasibility, appropriateness, uptake and cost perceptions of Family Mid- Upper Arm Circumference screening supported by two-way SMS in western Kenya” to more clearly convey that the intervention involves caregiver-led screening for acute malnutrition rather than MUAC measurement alone.

Response: Thank you for this suggestion. We have retained Family MUAC in the title because it is the established term used in the literature to describe this caregiver-led approach to screening children for acute malnutrition through mid-upper arm circumference measurement. Therefore, we believe that adding “screening” to the title would be somewhat redundant. However, we agree that it is important to clearly convey the caregiver-led screening nature of the intervention and have ensured that this is clearly described in the manuscript.

One more point Please use consistent terminology throughout the manuscript (e.g., 'health workers' or 'healthcare workers') to improve clarity.

Response: We have corrected terminology to refer to healthcare workers throughout the manuscript.

Apart from these points, I believe the authors have responded satisfactorily to the reviewers' comments and the manuscript has been considerably improved.

Reviewer #13: The comparison between high and low interactors is a central component of the study. However, no socio-demographic characteristics are presented for these groups. Without such information, it is difficult to determine whether differences in engagement reflect behavioral determinants identified through the TDF or underlying socio-economic differences. Please discuss this limitation more explicitly.

Response: We acknowledge this as a limitation of the study. We did not collect additional demographic information from caregivers in the qualitative sample, in part to protect confidentiality. We have outlined the lack of reporting on demographic characteristics for interview participants in the discussion, on page 29:

*“We did not collect detailed socio-demographic characteristics of participants, which limits our ability to assess how factors such as literacy, socioeconomic status, or access to broader household resources may have influenced engagement with the intervention. Although we purposively sampled participants across sites and levels of engagement, the absence of these data restricts our ability to examine potential differences between groups.”*

We did however collect the same number of participants from each facility to ensure all sites had equal representation.

The statement that the World Health Organization (WHO) recommends integrating early detection and treatment of child wasting into routine primary health care services appears to be supported by a UNICEF guidance document. Please verify the citation and either provide an appropriate WHO reference or revise the text to accurately reflect the source of the recommendation.

Response: Thank you for flagging this citation. We have updated the citation to reflect the WHO document reflecting this recommendation.

The abbreviation SMS is defined differently in the manuscript. In the Abstract, SMS is expanded as “short message system,” whereas in the Introduction it is defined as “short message service.” Please ensure consistent and correct terminology throughout the manuscript.

Response: Thank you we have corrected the Abstract to reflect the correct definition of SMS, which is Short Message Service.

The manuscript would benefit from additional information regarding the qualifications and training of the research and healthcare personnel involved in the study. Although the interviewer’s educational background and experience in qualitative data collection are described, it would be helpful to clarify whether specific training was provided on the study procedures, use of the Theoretical Domains Framework, and qualitative interviewing techniques. In addition, please indicate whether the healthcare workers involved in the intervention had prior training and expertise in child nutrition, MUAC assessment, and malnutrition management, as these factors may have influenced implementation and participant experiences.

Response: We have added details about the qualitative researcher and health professionals who participate in the intervention as suggested. These additions can be found on page 6-7:

*“Prior to study commencement, the RA received training on study procedures, qualitative data collection guides, and the application of the Theoretical Domains Framework,”*

and page 5:

*“These trained health professionals involved in the intervention held prior training and experience in child nutrition, MUAC assessment, and management of acute malnutrition.”*

For readability, please avoid beginning the description of the interviewer with initials only ("MO"). Consider introducing the individual's full name and role at first mention and then using initials thereafter, if preferred.

Response: This has been updated.

The manuscript states that seven focus group discussions were conducted with healthcare workers from different professional backgrounds. However, it is unclear whether the focus groups were profession-specific or composed of mixed cadres. Please provide additional details regarding the composition of each focus group, as group structure may influence participant interaction and the nature of the data generated.

Response: Focus groups were only conducted with caregivers. No healthcare workers participated in focus group discussions. Healthcare workers were invited to participate in in-depth interviews by cadre as presented in Table 2 (pasted below). Interviews were profession specific.

| IDI participants | Interview location | | | Total interviews |
| --- | --- | --- | --- | --- |
|  | Migori County referral  Hospital | Homa Bay County Hospital | St. Joseph Mission Hospital |  |
| High SMS interactors (caregivers) | 2 | 3 | 2 | 7 |
| Low SMS interactors (caregivers) | 2 | 3 | 2 | 7 |
|  |  |  |  |  |
| Community health workers/counselors | 3 | 2 | 2 | 7 |
| Nurses | 2 | 2 | 1 | 5 |
| Study nutritionists | 1 | 1 |  | 2 |
| Ministry of Health staff/partners/nutritionists | 2 | 1 | 1 | 4 |
| TOTAL | | | | 32 |

Since the intervention combines caregiver-led MUAC monitoring and two-way SMS support, it is difficult to disentangle which component contributed most strongly to acceptability and uptake. This limitation should be emphasized more clearly.

Response: Thank you for this insight. We agree that the intervention combined caregiver-led Family MUAC monitoring with two-way SMS support. The objective of this study was to assess acceptability, uptake, and other perceptions of the intervention as a whole, rather than to disentangle the individual contributions of the Family MUAC and two-way SMS components. Therefore, we do not attribute the observed perceptions or levels of uptake to either component individually.

The manuscript provides valuable insights into the acceptability and implementation of the intervention. However, the discussion could more explicitly connect the identified themes to the positive outcomes observed in the parent trial. For example, which determinants of engagement (e.g., increased caregiver self-efficacy, earlier detection of wasting, improved caregiver–healthcare worker communication) are most likely to have contributed to the reported reduction in wasting? Strengthening this link would enhance the explanatory value of the qualitative findings.

Response: Thank you for this helpful comment. We agree that the qualitative findings can provide insight into potential pathways through which the intervention may have supported the positive outcomes observed in the parent trial. However, the objective and design of the present study were not intended to quantify or establish causal relationships between individual determinants of engagement and the reduction in wasting. Therefore, we cannot determine which specific determinants contributed most strongly to the trial outcome. The qualitative findings should instead be interpreted as identifying potential mechanisms and contextual factors that may help explain how caregivers engaged with the intervention, rather than establishing the contribution of individual determinants to the reduction in wasting.

1. Tickell, K.D. ∙ Diakhate, M.M. ∙ Goodman, J.L. ∙ et al. Impact of a two-way short message service (SMS) to support maternally administered childhood mid-upper arm circumference monitoring and expand malnutrition screening in Kenya: the Mama Aweza trial protocol. *BMJ Open.* 2020; 10, e036660
