# Supplementary material for: Acceptability, feasibility, appropriateness, uptake and cost perceptions of family mid-upper arm circumference supported by two-way SMS in western Kenya
Source: PLoS One. 2026 Sep 23;21(9):e0358775. doi: 10.1371/journal.pone.0358775 (PMC13600513; doi:10.1371/journal.pone.0358775)
Supplement: Peer review history

# Author comment: Author response to Decision Letter 0

**DOI:** 10.1371/journal.pone.0358775.r001

17 Dec 2025

Attachment Submitted filename: MAMMS SERU Approval Letter_3May2019.pdf

# Peer review: Decision Letter 0

**DOI:** 10.1371/journal.pone.0358775.r002

**Authors:** Arupendra Mozumdar

23 Feb 2026

Dear Dr. Means,

Thank you for submitting your manuscript to PLOS ONE. After careful consideration, we feel that it has merit but does not fully meet PLOS ONE’s publication criteria as it currently stands. Therefore, we invite you to submit a revised version of the manuscript that addresses the points raised during the review process.

Please submit your revised manuscript by Apr 09 2026 11:59PM. If you will need more time than this to complete your revisions, please reply to this message or contact the journal office at plosone@plos.org. When you're ready to submit your revision, log on to https://www.editorialmanager.com/pone/ and select the 'Submissions Needing Revision' folder to locate your manuscript file.

- A letter that responds to each point raised by the academic editor and reviewer(s). You should upload this letter as a separate file labeled 'Response to Reviewers'.
- A marked-up copy of your manuscript that highlights changes made to the original version. You should upload this as a separate file labeled 'Revised Manuscript with Track Changes'.
- An unmarked version of your revised paper without tracked changes. You should upload this as a separate file labeled 'Manuscript'.

If you would like to make changes to your financial disclosure, please include your updated statement in your cover letter. Guidelines for resubmitting your figure files are available below the reviewer comments at the end of this letter.

If applicable, we recommend that you deposit your laboratory protocols in protocols.io to enhance the reproducibility of your results. Protocols.io assigns your protocol its own identifier (DOI) so that it can be cited independently in the future. For instructions see: https://journals.plos.org/plosone/s/submission-guidelines#loc-laboratory-protocols. Additionally, PLOS ONE offers an option for publishing peer-reviewed Lab Protocol articles, which describe protocols hosted on protocols.io. Read more information on sharing protocols at https://plos.org/protocols?utm_medium=editorial-email&utm_source=authorletters&utm_campaign=protocols.

We look forward to receiving your revised manuscript.

Kind regards,

Arupendra Mozumdar, Ph.D., M.Sc.

Academic Editor

PLOS One

Journal Requirements:

When submitting your revision, we need you to address these additional requirements.

1. Please ensure that your manuscript meets PLOS ONE's style requirements, including those for file naming. The PLOS ONE style templates can be found at

https://journals.plos.org/plosone/s/file?id=wjVg/PLOSOne_formatting_sample_main_body.pdf and https://journals.plos.org/plosone/s/file?id=ba62/PLOSOne_formatting_sample_title_authors_affiliations.pdf

2. Please include a complete copy of PLOS’ questionnaire on inclusivity in global research in your revised manuscript. Our policy for research in this area aims to improve transparency in the reporting of research performed outside of researchers’ own country or community. The policy applies to researchers who have travelled to a different country to conduct research, research with Indigenous populations or their lands, and research on cultural artefacts. The questionnaire can also be requested at the journal’s discretion for any other submissions, even if these conditions are not met. Please find more information on the policy and a link to download a blank copy of the questionnaire here: https://journals.plos.org/plosone/s/best-practices-in-research-reporting. Please upload a completed version of your questionnaire as Supporting Information when you resubmit your manuscript.

3. Thank you for stating in your Funding Statement:

[This work was supported by the Thrasher Research Foundation (ID#14656) and an unrestricted gift from Pamela H. Fowler.].

Please provide an amended statement that declares *all* the funding or sources of support (whether external or internal to your organization) received during this study, as detailed online in our guide for authors at http://journals.plos.org/plosone/s/submit-now.  Please also include the statement “There was no additional external funding received for this study.” in your updated Funding Statement.

Please include your amended Funding Statement within your cover letter. We will change the online submission form on your behalf.

4. In the online submission form, you indicated that [The datasets used and/or analysed during the current study are available from the corresponding author upon request].

All PLOS journals now require all data underlying the findings described in their manuscript to be freely available to other researchers, either 1. In a public repository, 2. Within the manuscript itself, or 3. Uploaded as supplementary information.

This policy applies to all data except where public deposition would breach compliance with the protocol approved by your research ethics board. If your data cannot be made publicly available for ethical or legal reasons (e.g., public availability would compromise patient privacy), please explain your reasons on resubmission and your exemption request will be escalated for approval.

5. Please include a copy of Table 3 which you refer to in your text on page 18.

6. We note that this manuscript consists of interview transcripts. Can you please confirm that all participants gave consent for interview transcript to be published?

If they DID provide consent for these transcripts to be published, please also confirm that the transcripts do not contain any potentially identifying information (or let us know if the participants consented to having their personal details published and made publicly available). We consider the following details to be identifying information:

- Names, nicknames, and initials

- Age more specific than round numbers

- GPS coordinates, physical addresses, IP addresses, email addresses

- Information in small sample sizes (e.g. 40 students from X class in X year at X university)

- Specific dates (e.g. visit dates, interview dates)

- ID numbers

Or, if the participants DID NOT provide consent for these transcripts to be published:

- Provide a de-identified version of the data or excerpts of interview responses

- Provide information regarding how these transcripts can be accessed by researchers who meet the criteria for access to confidential data, including:

a) the grounds for restriction

b) the name of the ethics committee, Institutional Review Board, or third-party organization that is imposing sharing restrictions on the data

c) a non-author, institutional point of contact that is able to field data access queries, in the interest of maintaining long-term data accessibility.

d) Any relevant data set names, URLs, DOIs, etc. that an independent researcher would need in order to request your minimal data set.

For further information on sharing data that contains sensitive participant information, please see: https://journals.plos.org/plosone/s/data-availability#loc-human-research-participant-data-and-other-sensitive-data

If there are ethical, legal, or third-party restrictions upon your dataset, you must provide all of the following details (https://journals.plos.org/plosone/s/data-availability#loc-acceptable-data-access-restrictions):

1. A complete description of the dataset

2. The nature of the restrictions upon the data (ethical, legal, or owned by a third party) and the reasoning behind them

3. The full name of the body imposing the restrictions upon your dataset (ethics committee, institution, data access committee, etc)

4. If the data are owned by a third party, confirmation of whether the authors received any special privileges in accessing the data that other researchers would not have

5. Direct, non-author contact information (preferably email) for the body imposing the restrictions upon the data, to which data access requests can be sent

7. Please include captions for your Supporting Information files at the end of your manuscript, and update any in-text citations to match accordingly. Please see our Supporting Information guidelines for more information: http://journals.plos.org/plosone/s/supporting-information.

If the reviewer comments include a recommendation to cite specific previously published works, please review and evaluate these publications to determine whether they are relevant and should be cited. There is no requirement to cite these works unless the editor has indicated otherwise.

Additional Editor Comments:

Title:

Please write the full form of MUAC in the title.

Introduction,

Line #23. It is not clear, what does it mean by Family MUAC behavior. MUAC is an acronym and it is preceded with another noun ‘family’ and followed by another noun ‘behavior’. How a behavior linked to a circumference measurement is not clear. I would like to request authors to rephrase ‘Family MUAC behavior’ throughout the manuscript.

Moreover, throughout the manuscript there are other nouns attached to Family MUAC e.g. ‘Family MUAC program’. I guess authors want to mean program involving Family MUAC here. But the text are not clear.

Material and Method,

Line #44. Please use the full form of quan and qual. Kindly write clearly what do you mean by quantitative -> qualitative.

Line#61. Author mentioned that children with MUAC measurements between 12.5 and 14.0 cm were eligible to participate. Are those cutoff measurements at the time of recruitment? Or any time throughout the study. Please clarify.

Line #68: Please mention what are the cutoffs for measurements corresponding “red”, “yellow”, and “green” color.

Line#78. Not clear what does it mean by “study exit”. Is it after end of the study? Or did they discontinue their participation in the study.

Line #86-89. Please provide some insights on response rates of the caregivers so that the reader could get an idea on caregivers involvement in the program and how justified the cutoffs of more than 90% (high interactors) and less than 30% (low interactors) as criteria for participation.

Results

Please provide a table on number of participants in each FGD and classify the discussions by type of participants—caregivers and healthcare workers.

Please provide, how did you select the high interactors and low interactors. What were the criteria for such selection? This information would be helpful in future research and the replicability of the study.

Line 172. The number of participants of IDIs are relatively less; therefore, please provide breakdowns of number of participants in numbers instead of percentages.

Line 198. In continuation of my last comment, I recommend to rephrase the sentence as “XX caregivers out of YY, who were classified into the intervention group, felt comfortable or somewhat comfortable…”.

Line 276. Suggesting to rephrase. “…measurements was preferred over other platforms, such as WhatsApp or Facebook, mainly due to the ability to send SMS…”--adding a comma after Facebook.

Discussion

Please write how the learning could be incorporated into the current nutrition program in western Kenya.

[Note: HTML markup is below. Please do not edit.]

Reviewers' comments:

Reviewer's Responses to Questions

**Comments to the Author**

1. Is the manuscript technically sound, and do the data support the conclusions?

Reviewer #1: Yes

Reviewer #2: Yes

Reviewer #3: Yes

Reviewer #4: Partly

Reviewer #5: Yes

Reviewer #6: Yes

Reviewer #7: Yes

**********

2. Has the statistical analysis been performed appropriately and rigorously?

Reviewer #1: Yes

Reviewer #2: Yes

Reviewer #3: N/A

Reviewer #4: No

Reviewer #5: Yes

Reviewer #6: N/A

Reviewer #7: Yes

**********

3. Have the authors made all data underlying the findings in their manuscript fully available?

The PLOS Data policy

Reviewer #1: Yes

Reviewer #2: Yes

Reviewer #3: Yes

Reviewer #4: Yes

Reviewer #5: Yes

Reviewer #6: Yes

Reviewer #7: Yes

**********

4. Is the manuscript presented in an intelligible fashion and written in standard English?

Reviewer #1: Yes

Reviewer #2: Yes

Reviewer #3: Yes

Reviewer #4: No

Reviewer #5: Yes

Reviewer #6: Yes

Reviewer #7: Yes

**********

Reviewer #1: The manuscript is well written and adds important contextual and perceptive information to important quantitative results, and to improve the health of communities at lower costs to the health sector, by smartly using widely existent cellular phones. However, there are a few places where some additional descriptions and/or organization of the several contextual expressions from the FGDs/IDIs may be required for better understanding by the readers, to make it publishable. I have suggested a few of these improvements in the attached file. Thanks.

Reviewer #2: Thank you for submitting this article for review. I have no concerns about dual publication, ethics or content. It would have been helpful to have made reference to the original RCT that took place, prior to this qualitative research study.

Reviewer #3: This manuscript presents qualitative data from caregivers and care providers who participated in an explanatory sequential mixed methods trial assessing the acceptability, feasibility, uptake and cost perceptions of a Family MUAC (mid-upper arm circumference) approach for early diagnosis of pediatric wasting. The trial intervention, a two-way SMS communication component between caregiver and provider, was found to reduce wasting in children 5-12 months by 37% compared to standard of care. Qualitative data provided a more nuanced perspective on the intervention.

The manuscript is clear and well-structured. The qualitative study design, data collection and analysis have multiple strengths, including reference to the Theoretical Domains Framework for both topic guide and codebook development, use of interviewers who were not part of the intervention or trial, analysis by a two-person team representing both US and Kenya investigators with strategies to ensure intercoder agreement.

The findings are interesting and well-balanced. I was particularly struck by the sense that, for some caregivers, the SMS intervention increased their sense of empowerment, strengthened bonds between both caregiver and child - and caregiver and health worker. In a sense, the intervention strengthened some of the more positive outcomes of a "self-care" approach.

I did wonder whether "low interactors" were more likely to be individuals with lower literacy or with less financial means (since it seems like some had to share phones and found the intervention difficult.)

While the discussion touches somewhat on these points, I'd like to propose several minor points to consider:

1) Would it be possible to present some socio-demographic information in a tabular form overall, by level of interaction with the intervention (low vs high) - at least for the SSIs? (I acknowledge that the numbers will be small, but interesting to see whether any trends.)

2) In the discussion, you might consider including some discussion about self-care strategies, which can either enhance care for those with higher self-efficacy, or could burden and worsen care for others who don't possess the financial or technological means to use the intervention.

3) Finally, it might be useful to more directly reference findings from the MUAC trial in the introduction in order to clarify where the 37% reduction statistic comes from.

Otherwise, really nice paper!

Reviewer #4: � The term "wasting" is used without explanation, which might confuse readers unfamiliar with the term.

� The exact functioning of the SMS system (frequency, caregiver engagement, etc.) is not fully explained.

� It’s not clear whether the SMS component or the MUAC monitoring had a more significant effect on reducing wasting.

� The study mentions the importance of understanding behavioral factors but doesn’t delve into what those factors are.

� The findings are based on a specific region (western Kenya), limiting the ability to apply them universally.

� Focus is primarily on caregivers, with less emphasis on the role and insights of health workers in implementing Family MUAC.

� The study mentions cost savings but lacks a detailed cost-benefit analysis of the intervention.

� The reliance on caregiver-reported MUAC measurements could introduce inaccuracies.

� The participant sample is drawn from specific hospitals, limiting diversity in socio-economic and geographical contexts.

� The study only assesses short-term outcomes, missing insight into the long-term impact of the intervention.

� The study does not explore how cultural factors might influence caregiver behavior or the uptake of Family MUAC.

� The study focuses on caregivers in specific regions of Kenya (Homa Bay and Migori Counties), which might limit the ability to generalize findings to other regions or countries with different healthcare systems and socio-cultural contexts.

� Caregivers’ self-reported experiences, especially regarding the ease of using MUAC tapes and the effectiveness of the SMS system, may introduce bias, as caregivers may overstate their satisfaction or success.

� While the study distinguishes between high and low interactors, there is no clear explanation of how these groups were identified beyond their response rates to SMS messages, which could affect the interpretation of engagement drivers.

� The study highlights that low interactors faced challenges like confusion with inconsistent measurements and social stigma, but the causes of these barriers (e.g., lack of understanding or community influence) could be explored more deeply.

� While some caregivers faced societal resistance, especially from family members like mothers-in-law, the study could provide a more in-depth exploration of these cultural barriers and their impact on engagement.

� There was inconsistency in measurements (e.g., discrepancies like 13.8 cm and 14.0 cm for the same child), but the study doesn’t provide a clear strategy for addressing these measurement errors or reinforcing accurate techniques.

� The study does not explore long-term outcomes or sustainability beyond the 6-month period, which limits insight into whether behaviors and practices related to MUAC monitoring persist over time.

� The study attributes high acceptability and success largely to the SMS system, but it is not clear whether the SMS messages themselves, the MUAC monitoring, or both were equally responsible for reducing malnutrition.

� While health workers’ perceptions were explored, there is limited discussion about the practical challenges they faced, particularly in terms of workload (e.g., 30-90 minutes daily to respond to messages) and how this might impact the feasibility of scaling the intervention.

� Although the study mentions various healthcare worker roles (e.g., counselors, nurses, nutritionists), it doesn't analyze how these different roles impact the effectiveness or feasibility of the intervention.

� he interviews and focus groups were conducted at different times, which could introduce variability in responses and may not capture a consistent view of caregiver experiences over the course of the intervention.

Reviewer #5: This is an interesting study conducted among caregivers and health care workers to assess the acceptability, feasibility, appropriateness, uptake and cost perceptions of family MUAC supported by two way SMS in western Kenya.

The paper is well written, however, the authors need to clarify the following;

1. You mention that all participants invited to the IDIs participated, what informed the number of participants in the IDIs?

2. What were the demographic characteristics of the participants in the FGDs and IDIs?

3. Have a separate section for conclusion and limitations.

Reviewer #6: Very well written and important paper.

Review of the literature in the Introduction and Discussion sections: Very limited literature review on factors affecting uptake. While it is true that there is no other literature on uptake of Family MUAC specifically, there is literature on other studies that involve both measurement at the household / community levels, and reporting through mHealth applications. It would be informative to compare the findings of this study with these other studies, at least with regard to acceptability / feasibility of the reporting through the mHealth application.

I appreciate that the data analysis was conducted on translated transcripts. Nevertheless, it would be of interest to learn and understand how the MUAC measurement and the measuring tape were labeled, perceived and discussed in Luo, Kuria and//or Kiswahili. Sometimes the local translation of technologies can affect perceptions and uptake. For example, antenatal care is commonly translated as "weighing" in a number of African languages, leading women to conclude that weighing is the main activity, and not fully appreciating what else is involved.

Editorial comments

Lines 2 and 3

Nearly half of all deaths of children under five years of age are linked to undernutrition, primarily in low-and-middle-income countries

=> Change "low-and-middle-income countries" to "low- and middle-income countries"

Lines 78-79

After study exit, a purposive sample of caregivers were invited to participate in face-to-face summative FGDs and in-depth interviews (IDI)

=> What is the meaning of "summative FGD"?

Vocabulary: Use, used, using and because. There words occur very frequently e.g., because 22 times, used 10 times, using 12 times. When occurring multiple times in a sentence or paragraph, this can become distracting. In most cases, the words use, used and using can be eliminated altogether. For because, could be alternated with other words. Some examples are below.

Lines 93-95

Current: The individual interviews were used to assess health worker perceptions of Family MUAC supported by two way SMS, potential barriers to sustaining or scaling the program, and opportunities to improve the program moving forward.

Proposed: The individual interviews assessed health worker perceptions of Family MUAC supported by two way SMS, potential barriers to sustaining or scaling the program, and opportunities to improve the program moving forward.

Lines 109-114 => Used occurs three times in the paragraph

Lines 200-202

Current: Most caregivers were content to use MUAC to measure their child and considered the MUAC tape easy to use (TDF construct: beliefs about capability).

Proposed: Most caregivers were content to measure MUAC on their child, and considered the procedure with the MUAC tape easy to do (TDF construct: beliefs about capability).

Reviewer #7: Overall, I found the project very interesting and many strengths in the manuscript. A job well done! I do recommend though that the manuscript be shortened. I feel there are areas where the text can be condensed. I also feel it is important to include graphics and/or tables of your findings which would ease reading. I know this is a qualitative study where tables and graphics are less common in general, but there are still many possibilities to create tables and graphics or written materials – at least to represent the domains found. I know you have one (on line 194 which is good but could still be made a little more succinct) And I do see you organized the results section according to the categories of results. It just still has too much text in each section. You could even create another graphic with bubbles included the quoted responses supporting each category.

I have several other comments related to the manuscript:

Lines 38-40 I do not think are necessary in the introduction. I feel the introduction should end exclusively on the objective – this information is also described in the methods therefore I think you can just delete lines 38-40.

Regarding the methods section, I see you describe the Mama Aweza study which is definitely necessary, yet I think you can cite the original materials describing the mama Aweza trial simply after its first mention on line 54 and I would only describe the intervention and control group and would even recommend representing this information via graphic. I would also describe more the SMS intervention as this is really what you are addressing mainly in your results section.

Regarding the inclusion and exclusion criteria, here I would highlight this exclusively where you describe your study and not that of the Mama Aweza trial.

On line 74, be sure to cite the Kenya guidelines for treating malnutrition.

As I stated above, cite the mama Aweza trial sources the first time you mention the trial, and then you can simply delete lines 75-76.

On line 82, you can state the criteria for acute malnourishment which I understand was a MUAC below 11.5 cm?

Lines 98-100 I don’t think it is necessary to state the researcher’s credentials here. And conflicts of interest are usually reported to the journal and therefore I do not find it necessary to include it in the manuscript

Lines 122-134 I feel can be condensed

In general, I understand the desire to explain all the coding process, but it also makes the manuscript a bit lengthy and hard to read.

The results really focus on SMS intervention but the methods focuses more on MUAC measurements and the process of the interviewing which is important but minimizes the focus on the SMS interventions which the results address. Maybe describe more about the content of the interview which could include just an outline of the topic categories and more importantly other aspects of interest pertaining to the SMS intervention.

I do see that on lines 64-66 you mention 2 weekly SMS – maybe highlight who reached out via SMS, how these arrangements were made in one line or two – excerpt or line to education message they received or a simple image.

As I mentioned above, the results still very long – I would shorten it to address specific arguments you wish to highlight.

Regarding the section on cost savings, I feel you should be a little more cautious of your interpretation as you have no quantitative analysis attached.

I found the discussion to be quite well written!

I am not sure why IRB approval letters are included? Of more interest would be graphics representing the results and study materials.

**********

what does this mean?). If published, this will include your full peer review and any attached files.

If you choose “no”, your identity will remain anonymous but your review may still be made public.

**Do you want your identity to be public for this peer review?**  For information about this choice, including consent withdrawal, please see our Privacy Policy

Reviewer #1: **Yes:** Dr. Alfredo L. Fort

Reviewer #2: No

Reviewer #3: **Yes:** Elizabeth E. Tolley

Reviewer #4: No

Reviewer #5: No

Reviewer #6: No

Reviewer #7: No

**********

[NOTE: If reviewer comments were submitted as an attachment file, they will be attached to this email and accessible via the submission site. Please log into your account, locate the manuscript record, and check for the action link "View Attachments". If this link does not appear, there are no attachment files.]

To ensure your figures meet our technical requirements, please review our figure guidelines: https://journals.plos.org/plosone/s/figures

You may also use PLOS’s free figure tool, NAAS, to help you prepare publication quality figures: https://journals.plos.org/plosone/s/figures#loc-tools-for-figure-preparation.

NAAS will assess whether your figures meet our technical requirements by comparing each figure against our figure specifications.

Attachment Submitted filename: PONE-D-25-63080_reviewer-AF.pdf

# Author comment: Author response to Decision Letter 1

**DOI:** 10.1371/journal.pone.0358775.r003

30 Apr 2026

Dear PlosOne editor,

We thank the editor and reviewers for their thoughtful feedback. We have carefully reviewed the comments and revised the manuscript accordingly. Our responses to the comments are included after each reviewer comment below:

Journal Requirements:

When submitting your revision, we need you to address these additional requirements.

1. Please ensure that your manuscript meets PLOS ONE's style requirements, including those for file naming. The PLOS ONE style templates can be found at

https://journals.plos.org/plosone/s/file?id=wjVg/PLOSOne_formatting_sample_main_body.pdf and https://journals.plos.org/plosone/s/file?id=ba62/PLOSOne_formatting_sample_title_authors_affiliations.pdf

We adhere to all the PLOS One style requirements including updating the file names.

2. Please include a complete copy of PLOS’ questionnaire on inclusivity in global research in your revised manuscript. Our policy for research in this area aims to improve transparency in the reporting of research performed outside of researchers’ own country or community. The policy applies to researchers who have travelled to a different country to conduct research, research with Indigenous populations or their lands, and research on cultural artefacts. The questionnaire can also be requested at the journal’s discretion for any other submissions, even if these conditions are not met. Please find more information on the policy and a link to download a blank copy of the questionnaire here: https://journals.plos.org/plosone/s/best-practices-in-research-reporting. Please upload a completed version of your questionnaire as Supporting Information when you resubmit your manuscript.

The copy of PLOS’ questionnaire on inclusivity in global research has been uploaded as Supporting Information as requested.

3. Thank you for stating in your Funding Statement:

[This work was supported by the Thrasher Research Foundation (ID#14656) and an unrestricted gift from Pamela H. Fowler.].

Please provide an amended statement that declares *all* the funding or sources of support (whether external or internal to your organization) received during this study, as detailed online in our guide for authors at http://journals.plos.org/plosone/s/submit-now. Please also include the statement “There was no additional external funding received for this study.” in your updated Funding Statement.

The funding statement has been included in the cover letter and updated as suggested to:

This work was supported by the Thrasher Research Foundation (ID#14656) and an unrestricted gift from Pamela H. Fowler. There was no additional external funding received for this study.

Please include your amended Funding Statement within your cover letter. We will change the online submission form on your behalf.

4. In the online submission form, you indicated that [The datasets used and/or analysed during the current study are available from the corresponding author upon request].

All PLOS journals now require all data underlying the findings described in their manuscript to be freely available to other researchers, either 1. In a public repository, 2. Within the manuscript itself, or 3. Uploaded as supplementary information.

This policy applies to all data except where public deposition would breach compliance with the protocol approved by your research ethics board. If your data cannot be made publicly available for ethical or legal reasons (e.g., public availability would compromise patient privacy), please explain your reasons on resubmission and your exemption request will be escalated for approval.

We provide de-identified excerpts of participant responses throughout the manuscript. Participants agreed to share de-identified excerpts from interviews and discussions but not entire transcripts. Since transcripts contain identifying information (e.g., caregiver names, location of interview, child names), we have included relevant de-identified transcript excerpts within the manuscript. Data can be accessed upon reasonable request from researchers who have obtained approvals to use it.

5. Please include a copy of Table 3 which you refer to in your text on page 18.

Thank you for bringing this to our attention. The reference to Table 3 has been removed

6. We note that this manuscript consists of interview transcripts. Can you please confirm that all participants gave consent for interview transcript to be published?

If they DID provide consent for these transcripts to be published, please also confirm that the transcripts do not contain any potentially identifying information (or let us know if the participants consented to having their personal details published and made publicly available). We consider the following details to be identifying information:

- Names, nicknames, and initials

- Age more specific than round numbers

- GPS coordinates, physical addresses, IP addresses, email addresses

- Information in small sample sizes (e.g. 40 students from X class in X year at X university)

- Specific dates (e.g. visit dates, interview dates)

- ID numbers

Or, if the participants DID NOT provide consent for these transcripts to be published:

- Provide a de-identified version of the data or excerpts of interview responses

- Provide information regarding how these transcripts can be accessed by researchers who meet the criteria for access to confidential data, including:

a) the grounds for restriction

b) the name of the ethics committee, Institutional Review Board, or third-party organization that is imposing sharing restrictions on the data

c) a non-author, institutional point of contact that is able to field data access queries, in the interest of maintaining long-term data accessibility.

d) Any relevant data set names, URLs, DOIs, etc. that an independent researcher would need in order to request your minimal data set.

For further information on sharing data that contains sensitive participant information, please see: https://journals.plos.org/plosone/s/data-availability#loc-human-research-participant-data-and-other-sensitive-data

If there are ethical, legal, or third-party restrictions upon your dataset, you must provide all of the following details (https://journals.plos.org/plosone/s/data-availability#loc-acceptable-data-access-restrictions):

1. A complete description of the dataset

2. The nature of the restrictions upon the data (ethical, legal, or owned by a third party) and the reasoning behind them

3. The full name of the body imposing the restrictions upon your dataset (ethics committee, institution, data access committee, etc)

4. If the data are owned by a third party, confirmation of whether the authors received any special privileges in accessing the data that other researchers would not have

5. Direct, non-author contact information (preferably email) for the body imposing the restrictions upon the data, to which data access requests can be sent

We confirm that participants agreed to share de-identified excerpts from interviews and discussions but not entire transcripts. Since there is identifying information throughout transcripts (e.g., children’s names), we have included relevant de-identified transcript excerpts within the manuscript. Data can be accessed upon reasonable request from researchers who have obtained approvals to use it.

7. Please include captions for your Supporting Information files at the end of your manuscript, and update any in-text citations to match accordingly. Please see our Supporting Information guidelines for more information: http://journals.plos.org/plosone/s/supporting-information.

We have updated captions for Supporting Information files at the end of the manuscript and in-text citations to match

If the reviewer comments include a recommendation to cite specific previously published works, please review and evaluate these publications to determine whether they are relevant and should be cited. There is no requirement to cite these works unless the editor has indicated otherwise.

Additional Editor Comments:

Title:

Please write the full form of MUAC in the title.

We have updated the manuscript to address this comment. The title now reads: “Acceptability, feasibility, appropriateness, uptake and cost perceptions of Family Mid-Upper Arm Circumference supported by two-way SMS in western Kenya”

Introduction,

Line #23. It is not clear, what does it mean by Family MUAC behavior. MUAC is an acronym and it is preceded with another noun ‘family’ and followed by another noun ‘behavior’. How a behavior linked to a circumference measurement is not clear. I would like to request authors to rephrase ‘Family MUAC behavior’ throughout the manuscript.

Thank you for pointing this out. We have removed the word ‘behavior’ describing Family MUAC to make this more clear. An example of the revised text can be found on page 3:

“To address this evidence gap, we conducted an explanatory sequential mixed methods study to understand determinants of Family MUAC in study sites in western Kenya.”

Moreover, throughout the manuscript there are other nouns attached to Family MUAC e.g. ‘Family MUAC program’. I guess authors want to mean program involving Family MUAC here. But the text are not clear.

Thank you for this comment. We have removed references to Family MUAC behaviors and updated the language to clarify about what is meant by Family MUAC programs. An example of the revised text can be found on page 2:

“This evidence is necessary for designing programs promoting Family MUAC at scale that are culturally and contextually appropriate, and most likely to prompt targeted behaviors.”

Material and Method,

Line #44. Please use the full form of quan and qual. Kindly write clearly what do you mean by quantitative -> qualitative.

Thank you for this feedback, our notation uses standard nomenclature for mixed methods studies. However, we provided additional explanation for what we mean by quan → QUAL. Specifically, we included the text below to provide additional clarity (page 3):

“In this approach, we first collected and analyzed quantitative data (quan), followed by the collection and analysis of qualitative data (QUAL) to explain, contextualize, or follow up on the initial quantitative results.”

Line#61. Author mentioned that children with MUAC measurements between 12.5 and 14.0 cm were eligible to participate. Are those cutoff measurements at the time of recruitment? Or any time throughout the study. Please clarify.

Thank you for this comment. These cutoffs are at the time of recruitment. This has been clarified in the manuscript and the text now reads as follows (page 4):

“Caregivers of children with MUAC measurements between 12.5 and 14.0 cm at the time of recruitment were eligible to participate.”

Line #68: Please mention what are the cutoffs for measurements corresponding “red”, “yellow”, and “green” color.

We have clarified this in the manuscript as requested. The revised text can be found on page 5:

“Measurements less than 11.5 cm were classified as red (severe acute malnutrition), between 11.5 cm and 12.5 cm as yellow (moderate acute malnutrition), and above 12.5 cm as green.”

Line#78. Not clear what does it mean by “study exit”. Is it after end of the study? Or did they discontinue their participation in the study.

To provide clarity, we have revised the text and removed the “study exit” term. The revised text can be found on page 5:

“After the 6 month follow up assessments had been completed, a purposive sample of caregivers were invited to participate in face-to-face summative FGDs and in-depth interviews (IDI)…”

Line #86-89. Please provide some insights on response rates of the caregivers so that the reader could get an idea on caregivers involvement in the program and how justified the cutoffs of more than 90% (high interactors) and less than 30% (low interactors) as criteria for participation.

We have included response rates and the median number of messages sent per caregiver in our findings regarding program’s uptake (page 17):

“Families randomized to the intervention arm had high uptake of the intervention with 94% responding to one text message or more over the period of enrollment [7]. Caregivers in the intervention sent a median of 20 messages (interquartile range (IQR): 15-24).”

The cutoffs were determined to identify an adequate sample of high interactors and low interactors to interview. To maximize heterogeneity of participants, we identified participants on the opposite ends of the participation spectrum, a common qualitative research method involving purposive sampling to understand drivers of adoption. To this end, we were able to juxtapose responses from those who interacted regularly with the SMS MAMMS system and those who chose to rarely interact with the system to identify barriers and facilitators to participation.

We have also provided further details in the Methods section to make this clearer (pages 5-6):

“We purposively sampled caregivers who interacted more with the platform and those who interacted less with the platform to identify factors that influence engagement among these two groups. We defined high interactors as caregivers who responded to >90% of the automated messages with MUAC measurements within seven days, while low interactors were caregivers who responded to <30% of the automated messages with MUAC measurements within seven days.”

Results

Please provide a table on number of participants in each FGD and classify the discussions by type of participants—caregivers and healthcare workers.

Thank you for this suggestion. We included two tables where we provide a) the number of caregivers participating in focus group discussions by study arm (Table 1), and b) the number of participants in the IDI by interview location and title (Table 2). These can be found on page 10.

Please provide, how did you select the high interactors and low interactors. What were the criteria for such selection? This information would be helpful in future research and the replicability of the study.

This has been clarified on pages 5-6. The text now reads as follows:

“We purposively sampled caregivers who interacted more with the platform and those who interacted less with the platform to identify factors that influence engagement among these two groups. We defined high interactors as caregivers who responded to >90% of the automated messages with MUAC measurements within seven days, while low interactors were caregivers who responded to <30% of the automated messages with MUAC measurements within seven days”.

Line 172. The number of participants of IDIs are relatively less; therefore, please provide breakdowns of number of participants in numbers instead of percentages.

Thank you for this suggestion. We changed the percentages to numbers and the revised text can be found on page 10:

“Seven high interactors and seven low interactors participated in IDIs which lasted 24 to 43 minutes. Additionally, 18 healthcare workers (HCW) participated in IDIs. Overall, seven were community health workers or counselors, five were nurses, two were nutritionists engaged in the study, and four were Kenyan Ministry of Health clinical staff. HCW interviews ran 25 to 85 minutes.”

We have also included Table 2 (page 10) where we provide the number of IDI participants by interview location and title.

Line 198. In continuation of my last comment, I recommend to rephrase the sentence as “XX caregivers out of YY, who were classified into the intervention group, felt comfortable or somewhat comfortable…”.

We have updated the text as requested. The revised text can be found on page 12:

“Of the caregivers randomized to the intervention group, 528 caregivers out of 532 felt very comfortable or somewhat comfortable using the MUAC tape.”

Line 276. Suggesting to rephrase. “…measurements was preferred over other platforms, such as WhatsApp or Facebook, mainly due to the ability to send SMS…”--adding a comma after Facebook.

We have revised the text as suggested (page 16):

“The use of SMS to send and receive measurements was preferred over other platforms, such as WhatsApp or Facebook, mainly due to the ability to send SMS without an internet connection.”

Discussion

Please write how the learning could be incorporated into the current nutrition program in western Kenya.

Thank you for this suggestion. We have expanded the discussion section and included the following information (page 26):

“In Kenya, mHealth solutions to support healthcare delivery have been outlined in the National eHealth Policy and can be a key strategy to reach rural areas across the country taking advantage of the high coverage of Kenya’s mobile networks [25].”

[Note: HTML markup is below. Please do not edit.]

Reviewers' comments:

Reviewer's Responses to Questions

Comments to the Author

Reviewer #1:

Was there an instruction as to carry out some medical intervention in case one measurement in the control group goes too low? (because ethically you cannot ignore a potential illness or mortality...)

Yes, when a measurement in the control group showed the child as acutely malnourished, HCW followed the CMAM Kenyan National Guidelines to provide medical intervention when needed and referral to a hospital for further care. On page 5, we have added, “Children diagnosed with wasting at any visit in both arms were provided treatment according to Kenya’s National Guidelines [16], with their progress monitored through scheduled follow-up visits.

It might be better to put these themes in a table, with a generic description, for better identification by the reader?...perhaps this is what is in Figure 1. Perhaps then try to anticipate that it will be combined in Fig 1...

Thank you for this thought. We have numbered the themes as suggested (in other comments) and presented them in Figure 1 for easier identification by the reader. We have also pulled out additional quotes for themes in separate tables (Tables 3-6).

Would it be possible to draw a figure highlighting how the determinants are related to the themes? (might be a bit complex but see if it can be made readable...that way you anticipate what will be discussed below)

Thank you for this thoughtful comment. A previous version of this figure highlighted the connections as suggested, but it was deemed too cumbersome as the visual lines connecting determinants and themes distracted from the overall message of this figure. The TDF determinants work together to make the intervention acceptable, feasible, appropriate, and perceived as cost savings for caregivers/HCW which is why we decided to use the figure as presented.

An important limitation is the qualitative nature of the study. It is partly acknowledged about the representativeness of the small sample, but there should also be said that there may be other "reasons" for success or lack of it that may not have been expressed by the people interviewed (e.g., some in the FGD may not have been "brave enough" so express some conflicts or deficiencies affecting their follow up during the intervention). This is openly known so it's not a reason invalidating the study at all, but speaks of the sincerity and knowledge by the authors...

Thank you for this suggestion. We acknowledge and agree with your comment about the challenges of conducting FGDs. On the other hand, the study team made every effort to make caregivers feel comfortable by conducting FGDs in a quiet setting and in a language of their choosing (Luo, KiSwahili, Kuria, English). We worked with a Kenyan qualitative researcher who is extensively trained in facilitation methods with a successful track record of conducting FGDs. She made efforts to encourage quieter participants to share, managing participants who were more outspoken and used extensive probing techniques to encourage caregivers to provide detailed responses, especially about barriers or conflicts.

Reviewer #2:

Thank you for submitting this article for review. I have no concerns about dual publication, ethics or content. It would have been helpful to have made reference to the original RCT that took place, prior to this qualitative research study.

Throughout the manuscript we mention characteristics and results from the original RCT. We have cited the study accordingly and references to this paper are cited under Tickell 2023 in the manuscript.

Full citation: Tickell KD, Achieng C, Masheti M, Anyango M, Ndirangu A, Diakhate MM, et al. Family MUAC supported by a two-way SMS platform for identifying children with wasting: the Mama Aweza randomised controlled trial. Eclinicalmedicine. 2023.

Examples include (page 2):

“Integrating Family MUAC with a two-way SMS platform may therefore represent an effective and affordable strategy to improve early identification of childhood wasting. To test this approach, we conducted a randomized controlled trial to test the effectiveness of a caregiver-administered MUAC monitoring system with direct communication to healthcare workers via short message system (SMS). The study found that Family MUAC supported by two-way SMS was associated with a 37% reduction in wasting in children 5-12 months compared to standard of care in western Kenya [1].”

Reviewer #3:

I did wonder whether "low interactors" were more likely to be individuals with lower literacy or with less financial means (since it seems like some had to share phones and found the intervention difficult.)

Thank you for this insightful observation. We agree that factors such as literacy and financial resources may influence engagement with the intervention. To minimize potential socio-demographic differences across groups, we purposively sampled an equal number of low and high interactors from each hospital site. However, we did not collect individual-level socio-demographic data as part of the qualitative interviews, which limits our ability to formally assess these differences. We have now acknowledged this limitation in the Discussion section (page 29).

While the discussion touches somewhat on these points, I'd like to propose several minor points to consider:

1) Would it be possible to present some socio-demographic information in a tabular form overall, by level of interaction with the intervention (low vs high) - at least for the SSIs? (I acknowledge that the numbers will be small, but interesting to see whether any trends.)

Thank you for this suggestion. We have added additional demographic information including the interview location for the semi-structured interviews by engagement level (Table 2; page 10). We sampled an equal number of high and low interactors from each facility (2 from Migori hospital, 3 from Homa Bay hospital, 2 from St. Joseph hospital) to ensure representation across sites. However, beyond interview location, no additional demographic information was collected from caregivers in the qualitative sample, in part to protect confidentiality. As such, we are unable to provide further tabulated socio-demographic comparisons between groups. We have now acknowledged this as a limitation in the Discussion section. We also agree that the qualitative sample size is small which makes it difficult to draw quantitative comparisons between the two groups of interactors.

2) In the discussion, you might consider including some discussion about self-care strategies, which can either enhance care for those with higher self-efficacy, or could burden and worsen care for others who don't possess the financial or technological means to use the intervention.

Thank you for this valuable suggestion. We have expanded our Discussion section and have now included discussion about self-care strategies as suggested. This can be found on page 29:

“These findings can also be interpreted through a self-care lens. Family MUAC supported by two-way SMS represents a form of guided self-care, where caregivers are empowered to monitor and respond to their child’s health needs with remote support from the health system. For caregivers with adequate resources, knowledge, and social support, this model can enhance self-efficacy, promote timely care-seeking, and reduce reliance on facility-based services [36]. However, for caregivers facing structural constraints, including limited access to mobile technology, low literacy, or competing financial demands, such interventions may inadvertently increase burden or exacerbate existing inequities [36]. This dual potential highlights the need for careful consideration of equity in the design and implementation of self-care interventions, ensuring that additional support mechanisms are in place for those with fewer resources.”

3) Finally, it might be useful to more directly reference findings from the MUAC trial in the introduction in order to clarify where the 37% reduction statistic comes from.

Thank you, we have added references to the MUAC trial manuscript (Tickell 2023) to the introduction.

Examples include (page 2):

“Integrating Family MUAC with a two-way SMS platform may therefore represent an effective and affordable strategy to improve early identification of childhood wasting. To test this approach, we conducted a randomized controlled trial to test the effectiveness of a caregiver-administered MUAC monitoring system with direct communication to healthcare workers via short message system (SMS). The study found that Family MUAC supported by two-way SMS was associated with a 37% reduction in wasting in children 5-12 months compared to standard of care in western Kenya [1].”

Full citation: Tickell KD, Achieng C, Masheti M, Anyango M, Ndirangu A, Diakhate MM, et al. Family MUAC supported by a two-way SMS platform for identifying children with wasting: the Mama Aweza randomised controlled trial. Eclinicalmedicine. 2023.

Reviewer #4:

� The term "wasting" is used without explanation, which might confuse readers unfamiliar with the term.

Thank you for pointing this out. We have further clarified the term at its first occurrence both in the abstract and in the introduction.

The revised text in the abstract now reads as follows:

“Early identification and treatment of acute malnutrition can decrease mortality and morbidity related to wasting, a form of malnutrition characterized by low weight-for-height.”

The revised text in the introduction can be found on page 1:

“Over 45 million children are estimated to be acutely malnourished or wasted (a form of malnutrition characterized by low weight-for-height) globally”

� The exact functioning of the SMS system (frequency, caregiver engagement, etc.) is not fully explained.

Thank you for highlighting this gap. In response, we have added more detail about the SMS system function under the Mama Aweza Trial description. The revised text can be found on pages 4-5:

“The SOC group did not receive MUAC tapes or SMS support. The weekly health-related educational SMS messages included content based on topics including developmental milestones, recommended hygiene and sanitation practices, vaccination schedules, home and kitchen gardening, prevention of malaria, and childhood illness danger signs and were tailored to the child’s developmental stage. SMS messages also encouraged caregivers to report their child’s measurement either with the MUAC measurement color (red, yellow, green) or MUAC measurement number in centimeters. Measurements less than 11.5 cm were classified as red (severe acute malnutrition), between 11.5 cm and 12.5 cm as yellow ( moderate acute malnutrition), and above 12.5 cm as green. When caregivers reported a child with a red or yellow measure, or MUAC below 12.5 cm, study staff including nutritionists and community health workers replied with a message to bring the child to the health facility for care. Messages were sent in a language chosen by caregivers (Luo, Kiswahili, Kuria or English). Caregivers sent and received messages at no cost to them.”

� It’s not clear whether the SMS component or the MUAC monitoring had a more significant effect on reducing wasting.

You are correct. This is because our study did not aim to disentangle the effects of the specific intervention components. Our study evaluated the acceptability, feasibility, appropriateness, uptake and cost perceptions of the two interventions components together (Family MUAC supported by two-way SMS).

� The study mentions the importance of understanding behavioral factors but doesn’t delve into what those factors are.

We applied the Theoretical Domains Framework (TDF), which is an integrative framework that captures behavioral factors influencing implementation. The TDF draws from behavioral theories and lays out 84 behavioral constructs across the 14 domains. Figure 1 (page 11) summarizes key behavioral factors under TDF determinants influencing caregiver engagement with family MUAC supported by SMS. For example, beliefs about capability, in particular self-efficacy, was a driving behavioral factor influencing acceptability of family MUAC. Caregivers considered the procedure with the MUAC tape easy to perform and were content measuring MUAC on their child (page 12). Another behavioral determinant was storing MUAC tape in a particular place making it easier for caregivers to measure their children reducing the chance of misplacing the tape (page 20) (TDF determinant: memory, attention, decision processes).

� The findings are based on a specific region (western Kenya), limiting the ability to apply them universally.

Thank you for this comment. We have acknowledged in the manuscript that given our sample characteristics, transferability of our findings to other contexts may be limited. However, it is important to note that the goal of qualitative research is not to produce universally generalizable results, but rather to explore topics in depth and provide rich, context-specific insights that can inform understanding of the issues under study.

� Focus is primarily on caregivers, with less emphasis on the role and insights of health workers in implementing Family MUAC.

Thank you for this comment. While we center caregiver perspectives throughout the manuscript, we have added insights from health care workers on the actions to make the intervention more sustainable and on the acceptability, feasibility, appropriateness, and uptake of the intervention. We highlighted their perspectives about integrating the intervention into workflows, the improvements to HCW-caregiver relationships from Family MUAC+SMS, and what is needed to improve implementing Family MUAC in the future.

� The study mentions cost savings but lacks a detailed cost-benefit analysis of the intervention.

Thank you for this insight. It was not within our scope to conduct a detailed cost-benefit analysis of the intervention. Our intention was to indicate that we were assessing perceptions of cost savings, as reflected in the study’s title. We observed that in some sentences this distinction was unclear, and we have revised the text to clarify this point.

For example, on page 3:

“The objectives of the caregiver focus group discussions (FGDs) and interviews were to assess acceptability, feasibility, appropriateness, and uptake of Family MUAC supported by two-way SMS, as well as caregivers’ perceptions of cost savings and drivers of engagement with the platform.”

Additionally, we included the following statement in the last paragraph of the discussion section (page 30):

“Moreover, we collected perceptions about cost savings from caregivers but do not report on a full economic evaluation to quantify these perceived cost savings. A cost-effectiveness analysis or cost-benefit analysis could provide further evidence of cost savings.”

� The reliance on caregiver-reported MUAC measurements could introduce inaccuracies.

We agree with this comment, as it was something discussed by the healthcare workers. We fully discuss this issue and noted this limitation in the discussion section.

Page 27:

“Despite these benefits, concerns regarding the accuracy and consistency of MUAC measurements emerged as a challenge. While many caregivers reported confidence in their ability to perform the measurements, concerns about consistency and accuracy were common. Since training on MUAC use was only provided at study onset, low interactors expressed frustration and confusion resulting from inconsistencies in measurement, which may have contributed to lower levels of engagement with the program. Ongoing training and regular refresher sessions could help to enhance the reliability of caregiver-collected data. HCWs emphasized the need for continuous guidance to address caregiver questions and ensure accurate measurements. Prior studies have also underscored the necessity of sustained training in community-based health interventions to maintain data integrity and program effectiveness [18, 30-32].”

Page 29:

“Additionally, the reliance on baseline training for caregivers introduced the potential for measurement errors, highlighting the need for ongoing skill reinforcement.”

We recommend ongoing training and regular refresher settings to enhance the reliability and accuracy of caregiver-reported MUAC measurements. We posit that caregiver led MUAC measurements at home can support and strengthen community management of acute malnutrition programs (CMAM) within Kenya. CMAM programs promote active case identification with community health workers visiting children at home to take MUAC measurements. Thus, caregiver-reported MUAC measurements would exist within the overall health system that does not require reliance on caregiver-reported MUAC measurements to track child growth.

� The participant sample is drawn from specific hospitals, limiting diversity in socio-economic and geographical contexts.

We agree with the issue regarding socioeconomic data , and it has been noted in the limitations of the study (pages 29-30):

“We did not collect detailed socio-demographic characteristics of participants, which limits our ability to assess how factors such as literacy, socioeconomic status, or access to broader household resources may have influenced engagement with the intervention. Although we purposively sampled participants across sites and levels of engagement, the absence of these data restricts our ability to examine potential differences between groups.”

Regarding diversity in geographical contexts, as noted above, the goal of qualitative research is not to produce universally generalizable results, but rather to explore topics in depth.

� The study only assesses short-term outcomes, missing insight into the long-term impact of the intervention.

Thank you for this important observation. We agree that understanding the long-term impact of the intervention is critical. However, the primary objective of this study was to assess implementation outcomes, including acceptability, feasibility, appropriateness, uptake, and perceived costs, using a qualitative approach. These outcomes are particularly relevant during the early stages of intervention evaluation, as they provide insight into how and why an intervention is used, and inform decisions about refinement and scale-up.

� The study does not explore how cultural factors might influence caregiver behavior or the uptake of Family MUAC.

We thank the reviewer for this perspective. One of our findings highlighted that caregivers considered mothers to be appropriate people to take MUAC measurements, because they spent the majority of the day with the child (pages 15-16). This recognition of maternal responsibility may have influenced the uptake of family MUAC. Additionally, we reported that some mothers in law were resistant to the idea of measuring children with MUAC tapes, reflecting a cultural factor identified by caregivers (page 18). In line with qualitative research principles, we followed participants’ perspectives and reported the factors they highlighted as most influential, including those shaped by cultural norms.

In response to this comment, we have expanded our Discussion section to frame our findings as part of social and cultural dynamics. The expanded discussion can be found on page 28.

“Social and cultural dynamics within households further influenced caregiver engagement. While some caregivers benefited from supportive family environments, others reported resistance or skepticism from key decision-makers, including spouses and mothers-in-law. Caregivers with supportive family members were more likely to remain active in the program, while those facing negative social influences or lacking support experienced difficulties maintaining participation. Additionally, prevailing gender norms that position women as primary caregivers may increase their responsibility for child health while limiting their autonomy in decision-making or access to resources [36]. Evidence from maternal and child nutrition programs suggests that engaging additional family members during program implementation, including male partners and senior female relatives, could enhance caregiver involvement and overall program success [21].”

� The study focuses on caregivers in specific regions of Kenya (Homa Bay and Migori Counties), which might limit the ability to generalize findings to other regions or countries with different healthcare systems and socio-cultural contexts.

In the limitations section of the discussion (page 29) we noted that the transferability of our study findings to contexts with different characteristics may be limited. However, as indicated above, consistent with qualitative research principles, the goal is not to produce universally generalizable results, but rather to explore topics in depth and provide rich, context-specific insights that may support transferability to similar settings.

� Caregivers’ self-reported experiences, especially regarding the ease of using MUAC tapes and the effectiveness of the SMS system, may introduce bias, as caregivers may overstate their satisfaction or success.

Thank you for this helpful thought. You are correct self-reported experience may introduce bias, which is one reason why we included interviews with health professionals who provide a more objective view of caregiver-led MUAC measurement. For example, as previously indicated, we noted that HCWs considered the initial training on caregiver use of MUAC as insufficient and recommended retraining (page 22):

“HCWs offered a different perspective on skills development. They mentioned that the initial training offered to caregivers on measuring MUAC was often insufficient to ensure high accuracy and reliability of measurements throughout the six-month study period. They emphasized the importance of building measurement skills through retraining.”

� While the study distinguishes between high and low interactors, there is no clear explanation of how these groups were identified beyond their response rates to SMS messages, which could affect the interpretation of engagement drivers.

We purposively sampled the same number of high and low interactors from each site (2 from Migori Hospital, 3 from Homa Bay hospital, 2 from St. Joseph Hospital, respectively). We added this detail in the methods section under Participant sampling (pages 5-6). However, other than by site and response rates to SMS, we did not use any other criteria for identifying high and low interactors. We have now acknowledged this limitation in the Discussion section (pages 29-30):

“We did not collect detailed socio-demographic characteristics of participants, which limits our ability to assess how factors such as literacy, socioeconomic status, or access to broader household resources may have influenced engagement with the intervention. Although we purposively sampled participants across sites and levels of engagement, the absence of these data restricts our ability to examine potential differences between groups.”

� The study highlights that low interactors faced challenges like confusion with inconsistent measurements and social stigma, but the causes of these barriers (e.g., lack of understanding or community influence) could be explored more deeply.

Thank you, this is an interesting observation. We will build more probing into future studies. Our objective was to identify barriers and facilitators of engagement with family MUAC with 2-way SMS but did not attempt to pursue a root cause analysis. We will note this to explore causes of these barriers for future work.

� While some caregivers faced societal resistance, especially from family members like mothers-in-law, the study could provide a more in-depth exploration of these cultural barriers and their impact on engagement.

Thank you for this important feedback. This is noted for future steps. We expanded our discussion on social and cultural dynamics that influenced our findings (page 28).

� There was inconsistency in measurements (e.g., discrepancies like 13.8 cm and 14.0 cm for the same child), but the study doesn’t provide a clear strategy for addressing these measurement errors or reinforcing accurate techniques.

As indicated above, we noted that HCWs considered the initial training on caregiver use of MUAC as insufficient and recommended retraining caregivers (page 22):

“HCWs offered a different perspective on skills development. They mentioned that the initial training offered to caregivers on measuring MUAC was often insufficient to ensure high accuracy and reliability of measurements throughout the six-month study period. They emphasized the importance of building measurement skills through retraining.”

We also highlighted the need to address these measurement errors through further caregiver training in the discussion section (page 27):

“Despite these benefits, concerns regarding the accuracy and consistency of MUAC measurements emerged as a challenge. While many caregivers reported confidence in their ability to perform the measurements, concerns about consistency and accuracy were common. Since training on MUAC use was only provided at study onset, low interactors expressed frustration and confusion resulting from inconsistencies in measurement, which may have contributed to lower levels of engagement with the program. Ongoing training and regular refresher sessions could help to enhance the reliability of caregiver-collected data. HCWs emphasized the need for continuous guidance to address caregiver questions and ensure accurate measurements. Prior studies have also underscored the necessity of sustained training in community-based health interventions to maintain data integrity and program effectiveness [18, 30-32].”

� The study does not explore long-term outcomes or sustainability beyond the 6-month period, which limits insight into whether behaviors and practices related to MUAC monitoring persist over time.

Again, thank you for this observation. The primary objective of this study was to assess implementation outcomes, including acceptability, feasibility, appropriateness, uptake, and perceived costs, using a qualitative approach. These outcomes are particularly relevant during the early stages of intervention evaluation, as they provide insight into how and why an intervention is used, and inform decisions about refinement and scale-up.

� The study attributes high acceptability and success largely to the SMS system, but it is not clear whether the SMS messages themselves, the MUAC monitoring, or both were equally responsible for reducing malnutrition.

Thank you for this comment. As indicated above, our study did not aim to disentangle the specific component that had a more significant effect on reducing malnutrition. Our study evaluated the acceptability, feasibility, appropriateness, uptake and cost perceptions of the two interventions components together (Family MUAC supported by two-way SMS).

� While health workers’ perceptions were explored, there is limited discussion about the practical challenges they faced, particularly in terms of workload (e.g., 30-90 minutes daily to respond to messages) and how this might impact the feasibility of scaling the intervention.

Thank you for this observation. While healthcare workers mentioned it took time to read and respond to messages on a daily basis, each provider was able to establish a workflow to engage with SMS messages in a way that fit their schedules. They reported that, with experience, responding to SMS messages became more efficient and feasible within routine workflows. Shifting follow-up communication and basic screening outside the clinic reduced pressure on high-volume MCH services. Healthcare workers mentioned that the intervention reduced workloads and the need for in-person follow-up and basic screening (page 24). Nonetheless, we acknowledge that to feasibly scale the intervention, additional demands may be placed on HCWs to review and respond to messages. We have added to page 27 to reflect these considerations:

“providing ongoing support through two-way SMS platforms may introduce additional demands on HCWs, particularly in terms of time required to review and respond to caregiver messages. Without careful program design, including appropriate staffing, task-shifting strategies, or automation of routine responses, these added responsibilities could increase workload and potentially limit the feasibility and scalability of such interventions within already resource-constrained health systems.”

� Although the study mentions various healthcare worker roles (e.g., counselors, nurses, nutritionists), it doesn't analyze how these different roles impact the effectiveness or feasibility of the intervention.

Thank you for this observation. It was not within the scope of our study to analyze how these different roles impacted the effectiveness of the intervention. We obtained overall impressions about using the SMS system and gathered feedback on feasibility of caregiver-led MUAC measurements. This is noted for future studies to elucidate how particular roles may have contributed to the effectiveness of the intervention.

� The interviews and focus groups were conducted at different times, which could introduce variability in responses and may not capture a consistent view of caregiver experiences over the course of the intervention.

Caregiver focus groups and interviews were conducted within two weeks (November 29 - December 7, 2021) to capture a consistent caregiver perspective. We conducted caregiver interviews in the same period to capture a cross-sectional snapshot of experiences. Healthcare worker interviews were carried out in July 2022, close to the end of the study, to capture overall insights from healthcare workers about interacting with the SMS system and feedback on caregiver-led MUAC measurement. These health worker interviews were intentionally held at this time to record overall impressions about the health care worker role over the course of the entire Mama Aweza trial period.

Reviewer #5: This is an interesting study conducted among caregivers and health care workers to assess the acceptability, feasibility, appropriateness, uptake and cost perceptions of family MUAC supported by two way SMS in western Kenya.

The paper is well written, however, the authors need to clarify the following;

1. You mention that all participants invited to the IDIs participated, what informed the number of participants in the IDIs?

Participants were invited based upon predefined inclusion criteria (e.g. role as a healthcare worker, nutritional status of child, SMS responsiveness of caregiver), which influenced the number of invitations extended. We invited all the staff who were involved in the study roll out and interacted with the SMS system to participate in the IDIs. The number of IDIs with healthcare workers was informed by the number of healthcare workers directly involved in the Mama Aweza trial in each site. For caregiver IDIs we sought to reach saturation with high/low engagers while balancing timing and budget. In focus groups, we aimed to have 5-7 participants per group, and the final number of participants in any given FGD was shaped in part by timing and availability.

2. What were the demographic characteristics of the participants in the FGDs and IDIs?

We did not collect demographic characteristics of the FGD and IDI participants. We have added a table detailing out the site where each IDI participant was sampled from (Table 2). We acknowledge the value of socio-demographic information for contextualizing findings and have now explicitly noted this as a limitation in the Discussion section (page 28).

3. Have a separate section for conclusion and limitations.

Thank you for this suggestion. We have separated out the conclusion into a separate section (page 30) that is distinct from the limitations section.

Reviewer #6: Very well written and important paper.

Review of the literature in the Introduction and Discussion sections: Very limited literature review on factors affecting uptake. While it is true that there is no other literature on uptake of Family MUAC specifically, there is literature on other studies that involve both measurement at the household / community levels, and reporting through mHealth applications. It would be informative to compare the findings of this study with these other studies, at least with regard to acceptability / feasibility of the reporting through the mHealth application.

Thank you very much for this important comment. We have revised both the Introduction and Discussion sections and expanded the literature review on the rationale for this study and factors affecting uptake. Some examples are included below:

Page 26:

“These findings are consistent with prior studies showing that mHealth interventions can reduce out-of-pocket expenditures and improve access to care by enabling remote consultations and follow-up [21, 22], particularly in rural and hard-to-reach settings [23, 24]. In many LMIC contexts, geographic distance and transportation challenges are key deterrents to care seeking, and mHealth solutions have been shown to mitigate these barriers by facilitating timely communication between caregivers and providers [24].”

Page 26:

“Moreover, having direct access to HCWs via SMS increased caregivers’ acceptance of the program and fostered improved patient-provider relationships, which has been identified as a key determinant of sustained engagement in digital health interventions [26].”

Page 28:

“Similar barriers have been identified in other mHealth studies [17, 19, 33, 34], where limited device ownership and concerns about privacy when using shared phones hinder participant engagement [34].”

I appreciate that the data analysis was conducted on translated transcripts. Nevertheless, it would be of interest to learn and understand how the MUAC measurement and the measuring tape were labeled, perceived and discussed in Luo, Kuria and//or Kiswahili. Sometimes the local translation of technologies can affect perceptions and uptake. For example, antenatal care is commonly translated as "weighing" in a number of African languages, leading women to conclude that weighing is the main activity, and not fully appreciating what else is involved.

MUAC measurement was explained to caregivers in their preferred language (Luo, Kiswahili, and, where applicable, Kuria) during training. The MUAC tape itself was not translated, as it uses standardized numeric values and color-coded zones (red, yellow, green) that support consistent and accurate reporting across participants, regardless of language or literacy level. The meanings of the colors and measurements were translated and clearly explained using locally appropriate terms, and training materials were provided in local languages to reinforce understanding. Caregivers generally described the process as measuring the child’s arm and identifying the color result, and we did not observe evidence of misinterpretation or inaccurate reporting related to language.

Editorial comments

Lines 2 and 3

Nearly half of all deaths of children under five years of age are linked to undernutrition, primarily in low-and-middle-income countries

=> Change "low-and-middle-income countries" to "low- and middle-income countries"

We have revised the text accordingly. It now reads as follows (page 1):

“Nearly half of all deaths of children under five years of age are linked to undernutrition, primarily in low-and middle-income countries (LMICs).”

Lines 78-79

After study exit, a purposive sample of caregivers were invited to participate in face-to-face summative FGDs and in-depth interviews (IDI)

=> What is the meaning of "summative FGD"?

Summative FGDs took place after study participation and intended to capture the full experience participating in the study involving sending and receiving text messages and use of MUAC to measure children.

Vocabulary: Use, used, using and because. There words occur very frequently e.g., because 22 times, used 10 times, using 12 times. When occurring multiple times in a sentence or paragraph, this can become distracting. In most cases, the words use, used and using can be eliminated altogether. For because, could be alternated with other words.

Thank you for pointing this out. We have carefully assessed the manuscript to avoid (excessively) using these words and alternated the word “because” with synonyms when appropriate.

Some examples are below.

Lines 93-95

Current: The individual interviews were used to assess health worker perceptions of Family MUAC supported by two way SMS, potential barriers to sustaining or scaling the program, and opportunities to improve the program moving forward.

Proposed: The individual interviews assessed health worker perceptions of Family MUAC supported by two way SMS, potential barriers to sustaining or scaling the program, and opportunities to improve the program moving forward.

This sentence has been updated.

Lines 109-114 => Used occurs three times in the paragraph

Thank you this paragraph has been updated.

Lines 200-202

Current: Most caregivers were content to use MUAC to measure their child and considered the MUAC tape easy to use (TDF construct: beliefs about capability).

Proposed: Most caregivers were content to measure MUAC on their child, and considered the procedure with the MUAC tape easy to do (TDF construct: beliefs about capability).

Thank you for this suggestion. We have updated the text just as suggested (Page 12).

Reviewer #7: Overall, I found the project very interesting and many strengths in the manuscript. A job well done! I do recommend though that the manuscript be shortened. I feel there are areas where the text can be condensed. I also feel it is important to include graphics and/or tables of your findings which would ease reading. I know this is a qualitative study where tables and graphics are less common in general, but there are still many possibilities to create tables and graphics or written materials – at least to represent the domains found. I know you have one (on line 194 which is good but could still be made a little more succinct) And I do see you organized the results section according to the categories of results. It just still has too much text in each section. You could even create another graphic with bubbles included the quoted responses supporting each category.

Thank you for your thoughtful feedback and kind words. We greatly appreciate your suggestions and have worked on shortening the manuscript’s Results to improve readability. We also carefully considered your recommendations regarding tables and have added Tables 3-6 to provide illustrative quotes to support and shorten the main manuscript text in tabular form.

I have several other comments related to the manuscript:

Lines 38-40 I do not think are necessary in the introduction. I feel the introduction should end exclusively on the objective – this information is also described in the methods therefore I think you can just delete lines 38-40.

Thank you for highlighting this. The referenced lines have been deleted.

Regarding the methods section, I see you describe the Mama Aweza study which is definitely necessary, yet I think you can cite the original materials describing the mama Aweza trial simply after its first mention on line 54 and I would only describe the intervention and control group and would even recommend representing this information via graphic. I would also describe more the SMS intervention as this is really what you are addressing mainly in your results section.

We have added greater description of the SMS intervention and cited the original study protocol and primary results paper as suggested.

Regarding the inclusion and exclusion criteria, here I would highlight this exclusively where you describe your study and not that of the Mama Aweza trial.

We used the same inclusion and exclusion criteria as the Mama Aweza trial. We purposively sampled from the Mama Aweza trial participants.

On line 74, be sure to cite the Kenya guidelines for treating malnutrition.

We added this citation as indicated.

As I stated above, cite the mama Aweza trial sources the first time you mention the trial, and then you can simply delete lines 75-76.

We updated the manuscript accordingly and have deleted the lines accordingly.

On line 82, you can state the criteria for acute malnourishment which I understand was a MUAC below 11.5 cm?

We have included additional information to clarify the criteria for acute malnourishment. It reads as follows (page 5):

“Measurements less than 11.5 cm were classified as red (severe acute malnutrition), between 11.5 cm and 12.5 cm as yellow ( moderate acute malnutrition), and above 12.5 cm as green.”

Lines 98-100 I don’t think it is necessary to state the researcher’s credentials here. And conflicts of interest are usually reported to the journal and therefore I do not find it necessary to include it in the manuscript

Thank you, this is a requirement for the COREQ checklist for qualitative research. This has been included to fulfill this requirement.

Lines 122-134 I feel can be condensed

We have condensed these lines, though noting that many of these details are required for the COREQ checklist for qualitative research.

In general, I understand the desire to explain all the coding process, but it also makes the manuscript a bit lengthy and hard to read.

This section has been condensed significantly to improve readability.

The results really focus on SMS intervention but the methods focuses more on MUAC measurements and the process of the interviewing which is important but minimizes the focus on the SMS interventions which the results address. Maybe describe more about the content of the interview which could include just an outline of the topic categories and more importantly other aspects of interest pertaining to the SMS intervention.

I do see that on lines 64-66 you mention 2 weekly SMS – maybe highlight who reached out via SMS, how these arrangements were made in one line or two – excerpt or line to education message they received or a simple image.

Thank you for highlighting this. We have added more detail on the SMS intervention including frequency and content of health messages included in texts. Modifications can be observed on page 4.

As I mentioned above, the results still very long – I would shorten it to address specific arguments you wish to highlight.

Thank you for this suggestion. We revised the manuscript accordingly to reduce the word count within the results section.

Regarding the section on cost savings, I feel you should be a little more cautious of your interpretation as you have no quantitative analysis attached.

Thank you for pointing this out. We took care to mention that participants perceived they saved money and made sure that throughout the manuscript this refers to perceptions. Additionally, in the Discussion section we have included the following statement, (page 30):

“Moreover, we collected perceptions about cost savings from caregivers but do not report on a full economic evaluation to quantify these perceived cost savings. A cost effectiveness analysis or cost-benefit analysis could provide further evidence of cost savings.”

I am not sure why IRB approval letters are included? Of more interest would be graphics representing the results and study materials.

The IRB approval letters were requested by PlosOne during submission, we will not be including them in as Supporting Information. We have included additional tables that represent the results.

Attachment Submitted filename: MAMMS_SERU_Approval_Letter_3May2019_auresp_1.pdf

# Peer review: Decision Letter 1

**DOI:** 10.1371/journal.pone.0358775.r004

**Authors:** Arupendra Mozumdar

19 Jul 2026

Dear Dr. Means,

Thank you for submitting your manuscript to PLOS ONE. After careful consideration, we feel that it has merit but does not fully meet PLOS ONE’s publication criteria as it currently stands. Therefore, we invite you to submit a revised version of the manuscript that addresses the points raised during the review process.

Please submit your revised manuscript by Sep 02 2026 11:59PM. If you will need more time than this to complete your revisions, please reply to this message or contact the journal office at plosone@plos.org. When you're ready to submit your revision, log on to https://www.editorialmanager.com/pone/ and select the 'Submissions Needing Revision' folder to locate your manuscript file.

- A letter that responds to each point raised by the academic editor and reviewer(s). You should upload this letter as a separate file labeled 'Response to Reviewers'.
- A marked-up copy of your manuscript that highlights changes made to the original version. You should upload this as a separate file labeled 'Revised Manuscript with Track Changes'.
- An unmarked version of your revised paper without tracked changes. You should upload this as a separate file labeled 'Manuscript'.

If you would like to make changes to your financial disclosure, please include your updated statement in your cover letter. Guidelines for resubmitting your figure files are available below the reviewer comments at the end of this letter.

If applicable, we recommend that you deposit your laboratory protocols in protocols.io to enhance the reproducibility of your results. Protocols.io assigns your protocol its own identifier (DOI) so that it can be cited independently in the future. For instructions see: https://journals.plos.org/plosone/s/submission-guidelines#loc-laboratory-protocols. Additionally, PLOS ONE offers an option for publishing peer-reviewed Lab Protocol articles, which describe protocols hosted on protocols.io. Read more information on sharing protocols at https://plos.org/protocols?utm_medium=editorial-email&utm_source=authorletters&utm_campaign=protocols.

As the corresponding author, your ORCID iD is verified in the submission system and will appear in the published article. PLOS supports the use of ORCID, and we encourage all coauthors to register for an ORCID iD and use it as well. Please encourage your coauthors to verify their ORCID iD within the submission system before final acceptance, as unverified ORCID iDs will not appear in the published article. *Only*  the individual author can complete the verification step; PLOS staff *cannot*  verify ORCID iDs on behalf of authors.

We look forward to receiving your revised manuscript.

Kind regards,

Arupendra Mozumdar, Ph.D., M.Sc.

Academic Editor

PLOS One

Journal Requirements:

If the reviewer comments include a recommendation to cite specific previously published works, please review and evaluate these publications to determine whether they are relevant and should be cited. There is no requirement to cite these works unless the editor has indicated otherwise.

Please review your reference list to ensure that it is complete and correct. If you have cited papers that have been retracted, please include the rationale for doing so in the manuscript text, or remove these references and replace them with relevant current references. Any changes to the reference list should be mentioned in the rebuttal letter that accompanies your revised manuscript. If you need to cite a retracted article, indicate the article’s retracted status in the References list and also include a citation and full reference for the retraction notice.

Additional Editor Comments :

We have received the comments and suggestions from the reviewers on the revised manuscript. I would like to request the authors to address the comments and incorporate the valuable suggestions from the second round of the review.

[Note: HTML markup is below. Please do not edit.]

Reviewers' comments:

Reviewer's Responses to Questions

**Comments to the Author**

Reviewer #8: (No Response)

Reviewer #9: All comments have been addressed

Reviewer #10: All comments have been addressed

Reviewer #11: All comments have been addressed

Reviewer #12: All comments have been addressed

Reviewer #13: (No Response)

**********

2. Is the manuscript technically sound, and do the data support the conclusions?

Reviewer #8: Partly

Reviewer #9: Partly

Reviewer #10: Yes

Reviewer #11: Yes

Reviewer #12: Yes

Reviewer #13: (No Response)

**********

3. Has the statistical analysis been performed appropriately and rigorously?

Reviewer #8: N/A

Reviewer #9: N/A

Reviewer #10: Yes

Reviewer #11: Yes

Reviewer #12: Yes

Reviewer #13: (No Response)

**********

4. Have the authors made all data underlying the findings in their manuscript fully available?

The PLOS Data policy

Reviewer #8: Yes

Reviewer #9: Yes

Reviewer #10: Yes

Reviewer #11: Yes

Reviewer #12: Yes

Reviewer #13: (No Response)

**********

5. Is the manuscript presented in an intelligible fashion and written in standard English?

Reviewer #8: Yes

Reviewer #9: Yes

Reviewer #10: Yes

Reviewer #11: Yes

Reviewer #12: Yes

Reviewer #13: (No Response)

**********

Reviewer #8: Comment 1

The study relies exclusively on self-reported perceptions of cost savings rather than a formal economic evaluation. While caregivers expressed that the intervention reduced transportation expenses, no cost-effectiveness or cost-benefit analysis was conducted to substantiate these claims. This limits the ability to draw robust conclusions about the actual financial impact of the intervention.

Comment 2

The definition of high and low engagement was based solely on response rates to SMS messages (above 90% versus below 30%). However, response frequency does not necessarily reflect meaningful engagement, understanding, or correct performance of MUAC measurements. A more nuanced composite measure of engagement would have strengthened the analysis.

Comment 3

The purposive sampling strategy for interviews was clearly described, yet the absence of detailed sociodemographic data (e.g., literacy level, household wealth, maternal education) prevents an assessment of whether specific subgroups differentially benefited or struggled with the intervention. This omission weakens the ability to comment on equity implications.

Comment 4

The study acknowledges that caregivers needed to be literate or have access to someone who could read and send SMS messages. This represents a substantial selection bias, and the findings may not be transferable to settings with lower literacy rates or less access to mobile phones. The manuscript would benefit from a more explicit discussion of how this limits generalisability.

Comment 5

Concerns about measurement accuracy were raised primarily by low-interactor caregivers and health workers, yet the study did not independently verify caregiver-performed MUAC measurements against a gold standard. Without objective validation, it remains unclear whether the reported reductions in wasting were attributable to accurate home monitoring or to other factors.

Comment 6

The Theoretical Domains Framework was appropriately applied to guide data collection and analysis. However, the presentation of results would be strengthened by a more systematic mapping of specific TDF constructs to individual themes, rather than the relatively high-level grouping currently provided.

Comment 7

The finding that some caregivers mistakenly believed SMS messages incurred a cost, which reduced engagement, is important. Yet the manuscript does not report whether any efforts were made during the trial to correct this misunderstanding proactively. This represents a missed opportunity to improve intervention design.

Comment 8

Health workers reported spending between 30 minutes and 1.5 hours daily responding to messages, with peaks of up to 50 messages per day. The feasibility of scaling this intervention to routine health system settings, where staff already face heavy workloads, was not adequately explored. This raises concerns about sustainability outside of a research context.

Comment 9

Social support from family members, particularly husbands and children, emerged as a strong facilitator of engagement, while resistance from mothers-in-law was a barrier. The study does not propose specific strategies to address negative social influences or to engage resistant family members in future implementations.

Comment 10

The intervention's potential to empower caregivers and transform them into community health advocates is compelling. However, the data supporting this claim are largely anecdotal, consisting of a few illustrative quotes. No systematic attempt was made to measure the extent or impact of this community-level diffusion of knowledge and skills.

Comment 11

Phone sharing was identified as a significant barrier to uptake, yet the manuscript does not discuss whether alternative communication channels (e.g., voice calls, community health worker visits, or group messaging) were considered or tested for caregivers without private phone access. This limits the practical relevance of the findings for the most disadvantaged populations.

Comment 12

The conclusion states that the intervention is "acceptable, feasible, and appropriate" based on qualitative data from a single region in western Kenya. While these findings are valuable for hypothesis generation, the lack of quantitative generalizability data or a formal implementation outcomes framework means the conclusions should be framed more cautiously, acknowledging the context-specific nature of the results.

Reviewer #9: Introduction:

Page 3 Line 43: It seems that the present study is only qualitative and not a mixed methods one. No quantitative data is provided.

Methods:

How was validity and reliability of qualitative data gathered determined?

Results:

By presenting the number of each group in the text, Table 1 can be removed:

“(1) caregivers in the intervention group whose child did not develop acute malnutrition (n=12), (2) caregivers in the intervention group whose child became acutely malnourished (n=13), (3) SOC participants whose child did not develop acute malnutrition (n=13), and (4) SOC participants whose child became acutely malnourished over the duration of the study (n=13).”

Despite the claim of authors for using mixed method, only the results of qualitative study have been presented.

Although tables have been added to the text of qualitative findings at the suggestion of the previous reviewers, the way of presenting the findings is not appropriate and it is better to present qualitative tables containing themes and sub-themes.

Discussion:

The following related article is not referenced:

Abdissa, H.G.; Duguma, G.B.; Gerbaba, M.; Noll, J.; Sori, D.A.; Koricha, Z.B. Acceptability, Feasibility, and Appropriateness of Mobile Phone Messaging-Based Message-Framing Intervention for Promoting Maternal and Newborn Care Practices. Int. J. Environ. Res. Public Health 2025, 22, 864. https://doi.org/10.3390/ijerph22060864.

Reviewer #10: In review of the article, the authors have taken into account the vast majority of previous peer review comments and made the requested improvements where noted in the article. In particular, the explanations in the methods section, the more detailed presentation of participant characteristics, the expansion of limitations, and the clarification of various conceptual points have increased the publishability of the study.

However, although the Discussion section has been supplemented with additional sources as mentioned in the previous revision, the improvement remains limited, and a more detailed discussion of the similarities and differences between the findings and previous studies, particularly regarding caregiver involvement, measurement accuracy, social support mechanisms, and the sustainability of the intervention, would be better.

Also, in response to the previous revision, while tables regarding the number of FGD participants have been added, reporting the number of participants in each focus group interview separately would be a more transparent approach.

Furthermore, the lack of sociodemographic data for participants is a significant limitation of the study. Therefore, it was not possible to assess potential differences between high and low-interaction participants in terms of age, education level, socioeconomic status, or other individual characteristics. This partially limits the interpretation and transferability of the findings.

Overall, the revisions made are satisfactory, and the remaining issues are minor improvements. Therefore, I believe the article can be published after minor revisions.

Reviewer #11: The methodology section presented in " PONE-D-25-63080_R1.docx" is highly rigorous, well-structured, and directly aligned with your study’s objectives. The choice of an explanatory sequential mixed-methods design (quan → QUAL) is highly appropriate for contextualising the quantitative trial results through the lived experiences of the caregivers.

Furthermore, your precise, metrics-driven purposive sampling strategy which uses stratifying by clinical outcomes and utilizing strict mathematical thresholds for high and low platform engagement significantly strengthens the comparative validity of your findings. The systematic application of the Theoretical Domains Framework (TDF) combined with a robust dual-coder analysis framework ensures excellent theoretical and analytical depth.

two minor comments:

1. Page 20, title: it would be better if you avoid using the acronym SMS in the title to improve readability.

2. As a minor formatting note for the final layout, please ensure that the title and caption for Figure 1 are placed directly below the figure, as is customarily done in academic writing.

Reviewer #12: The authors have responded comprehensively to the reviewers' comments and have addressed the majority of the concerns raised during peer review. Clarifications regarding participant eligibility, MUAC color classifications, study procedures, intervention implementation, limitations, and contextualization of the findings have substantially improved the manuscript.

However, I still have two minor concerns. First, the relationship between the previously published RCT and the current qualitative study remains somewhat unclear. I think some clarity is required in study design. I believe the manuscript is essentially a qualitative follow-up study informed by trial data.

I want to know the qualitative component was planned from the outset as part of a mixed-methods design, or the qualitative study was conducted later as a separate follow-up study. Because the relationship between the current qualitative study and the previously published RCT is not immediately clear from the abstract unless a reader is familiar with the earlier publication.

The manuscript would benefit from greater clarity regarding the overall study design specially for readers not familiar with previous publication if authors can please clarify whether the study was originally conceptualized and conducted as a mixed-methods study (with both quantitative and qualitative components planned a priori) or whether the qualitative work was undertaken subsequently?

Second, while the authors have acknowledged the absence of socio-demographic information as a limitation, factors such as literacy, education, phone ownership, and socioeconomic status appear highly relevant to understanding differences between high and low interactors in an SMS-based intervention. Consideration of these characteristics would have strengthened interpretation of the findings and identification of barriers to engagement.

I also have a suggestion regarding title and abstract: Authors should mention study design in the abstract

Abstract word count is 315. I suppose it shoudnt exceed 300? The abstract doesn’t reflect it is a mixed method clearly!

The title may benefit from replacing Family MUAC with "family MUAC screening" in the study title like “Acceptability, feasibility, appropriateness, uptake and cost perceptions of Family Mid- Upper Arm Circumference screening supported by two-way SMS in western Kenya” to more clearly convey that the intervention involves caregiver-led screening for acute malnutrition rather than MUAC measurement alone.

One more point Please use consistent terminology throughout the manuscript (e.g., 'health workers' or 'healthcare workers') to improve clarity.

Apart from these points, I believe the authors have responded satisfactorily to the reviewers' comments and the manuscript has been considerably improved.

Reviewer #13: The comparison between high and low interactors is a central component of the study. However, no socio-demographic characteristics are presented for these groups. Without such information, it is difficult to determine whether differences in engagement reflect behavioral determinants identified through the TDF or underlying socio-economic differences. Please discuss this limitation more explicitly.

The statement that the World Health Organization (WHO) recommends integrating early detection and treatment of child wasting into routine primary health care services appears to be supported by a UNICEF guidance document. Please verify the citation and either provide an appropriate WHO reference or revise the text to accurately reflect the source of the recommendation.

The abbreviation SMS is defined differently in the manuscript. In the Abstract, SMS is expanded as “short message system,” whereas in the Introduction it is defined as “short message service.” Please ensure consistent and correct terminology throughout the manuscript.

The manuscript would benefit from additional information regarding the qualifications and training of the research and healthcare personnel involved in the study. Although the interviewer’s educational background and experience in qualitative data collection are described, it would be helpful to clarify whether specific training was provided on the study procedures, use of the Theoretical Domains Framework, and qualitative interviewing techniques. In addition, please indicate whether the healthcare workers involved in the intervention had prior training and expertise in child nutrition, MUAC assessment, and malnutrition management, as these factors may have influenced implementation and participant experiences.

The comparison between high and low interactors is a central component of the study. However, no socio-demographic characteristics are presented for these groups. Without such information, it is difficult to determine whether differences in engagement reflect behavioral determinants identified through the TDF or underlying socio-economic differences. Please discuss this limitation more explicitly.

For readability, please avoid beginning the description of the interviewer with initials only ("MO"). Consider introducing the individual's full name and role at first mention and then using initials thereafter, if preferred.

The manuscript states that seven focus group discussions were conducted with healthcare workers from different professional backgrounds. However, it is unclear whether the focus groups were profession-specific or composed of mixed cadres. Please provide additional details regarding the composition of each focus group, as group structure may influence participant interaction and the nature of the data generated.

Since the intervention combines caregiver-led MUAC monitoring and two-way SMS support, it is difficult to disentangle which component contributed most strongly to acceptability and uptake. This limitation should be emphasized more clearly.

The manuscript provides valuable insights into the acceptability and implementation of the intervention. However, the discussion could more explicitly connect the identified themes to the positive outcomes observed in the parent trial. For example, which determinants of engagement (e.g., increased caregiver self-efficacy, earlier detection of wasting, improved caregiver–healthcare worker communication) are most likely to have contributed to the reported reduction in wasting? Strengthening this link would enhance the explanatory value of the qualitative findings.

**********

what does this mean?). If published, this will include your full peer review and any attached files.

If you choose “no”, your identity will remain anonymous but your review may still be made public.

**Do you want your identity to be public for this peer review?**  For information about this choice, including consent withdrawal, please see our Privacy Policy

Reviewer #8: No

Reviewer #9: No

Reviewer #10: No

Reviewer #11: **Yes:** Samidi Nirasha Navaratna, MD

Reviewer #12: No

Reviewer #13: **Yes:** hülya oğur

**********

[NOTE: If reviewer comments were submitted as an attachment file, they will be attached to this email and accessible via the submission site. Please log into your account, locate the manuscript record, and check for the action link "View Attachments". If this link does not appear, there are no attachment files.]

To ensure your figures meet our technical requirements, please review our figure guidelines: https://journals.plos.org/plosone/s/figures

You may also use PLOS’s free figure tool, NAAS, to help you prepare publication quality figures: https://journals.plos.org/plosone/s/figures#loc-tools-for-figure-preparation.

NAAS will assess whether your figures meet our technical requirements by comparing each figure against our figure specifications.

# Author comment: Author response to Decision Letter 2

**DOI:** 10.1371/journal.pone.0358775.r005

2 Sep 2026

Dear Editor,

Thank you for sharing additional reviewer comments on our manuscript, “Acceptability, feasibility, appropriateness, uptake and cost perceptions of Family Mid-Upper Arm Circumference supported by two-way SMS in western Kenya”. Please find our responses to each comment below:

Reviewer #8: Comment 1

The study relies exclusively on self-reported perceptions of cost savings rather than a formal economic evaluation. While caregivers expressed that the intervention reduced transportation expenses, no cost-effectiveness or cost-benefit analysis was conducted to substantiate these claims. This limits the ability to draw robust conclusions about the actual financial impact of the intervention.

Response: Thank you for this comment. We acknowledge that a formal economic evaluation was not conducted and acknowledge this limitation in the discussion section on page 30:

“we collected perceptions about cost savings from caregivers but do not report on a full economic evaluation to quantify these perceived cost savings. A cost-effectiveness analysis or cost-benefit analysis could provide further evidence of cost savings.”

Comment 2

The definition of high and low engagement was based solely on response rates to SMS messages (above 90% versus below 30%). However, response frequency does not necessarily reflect meaningful engagement, understanding, or correct performance of MUAC measurements. A more nuanced composite measure of engagement would have strengthened the analysis.

Response: Thank you for this thoughtful suggestion. We were interested in capturing engagement with the two-way SMS system between caregivers and healthcare workers. According to the data collected from program participants, this was captured through response frequency. You are correct that a more nuanced definition of engagement could have been applied, but we did not aim to capture accuracy of measurements as part of engagement. Instead, our engagement measure sought to capture caregivers’ sustained and consistent sharing of MUAC measurements via the two-way SMS system over the course of the study period.

Comment 3

The purposive sampling strategy for interviews was clearly described, yet the absence of detailed sociodemographic data (e.g., literacy level, household wealth, maternal education) prevents an assessment of whether specific subgroups differentially benefited or struggled with the intervention. This omission weakens the ability to comment on equity implications.

Response: Thank you for this suggestion. We did not attempt to comment on equity implications due to our inability to present sociodemographic data on sampled participants. We acknowledge this as a limitation in our study. On page 29, we indicate:

“We did not collect detailed socio-demographic characteristics of participants, which limits our ability to assess how factors such as literacy, socioeconomic status, or access to broader household resources may have influenced engagement with the intervention. Although we purposively sampled participants across sites and levels of engagement, the absence of these data restricts our ability to examine potential differences between groups.”

Comment 4

The study acknowledges that caregivers needed to be literate or have access to someone who could read and send SMS messages. This represents a substantial selection bias, and the findings may not be transferable to settings with lower literacy rates or less access to mobile phones. The manuscript would benefit from a more explicit discussion of how this limits generalisability.

Response: Interestingly, no caregivers were excluded from the Mama Aweza study because they were unable to read or write or did not have someone who knew how to read or write. Reportedly, caregivers were not excluded because they did not have mobile phones but only that their phones did not use the compatible service provider (Safaricom; the most common provider in the region). Nonetheless, you are correct that this is a limitation of the intervention, and we acknowledge that this intervention may perform less well in areas of low literacy. We thank you for your comment and consider it pertinent, so we have added to our limitations section on page 30:

“In addition, the study enrolled caregivers who were literate or had access to someone to help them read and send SMS, which may limit transferability to other contexts with lower literacy rates or less access to mobile phones.”

Comment 5

Concerns about measurement accuracy were raised primarily by low-interactor caregivers and health workers, yet the study did not independently verify caregiver-performed MUAC measurements against a gold standard. Without objective validation, it remains unclear whether the reported reductions in wasting were attributable to accurate home monitoring or to other factors.

Response: To clarify, when a caregiver screened a child as wasted (red or yellow colored section of the MUAC tape) at home, caregivers were asked to bring the child to the health facility where a trained health professional confirmed the MUAC measurement. All caregiver-performed MUAC measurements identified as moderately or severely wasted were objectively verified by a study staff/trained health professional. If the caregiver reported yellow/red and it was verified by the trained staff to not be red/yellow, the caregiver received refresher MUAC training. Additional details have been added on page 5:

“Caregiver-performed MUAC measurements identified as moderate or severely wasted were verified by a trained health professional.”

Additionally: “children diagnosed with wasting, as confirmed by a health professional at any visit in both arms, were provided treatment according to Kenya’s National Guidelines, with their progress monitored through scheduled follow-up visits.”

Comment 6

The Theoretical Domains Framework was appropriately applied to guide data collection and analysis. However, the presentation of results would be strengthened by a more systematic mapping of specific TDF constructs to individual themes, rather than the relatively high-level grouping currently provided.

Response: Thank you, we identified the specific constructs that stood out as outlined in Figure 1 and in the additional tables added to the manuscript. For example, on Table 2 we highlighted the construct of person x environment interaction under the TDF domain environmental context and resources and provided illustrative quotes to support this finding. On Figure 1 we specified the construct Automatic/routine/habit under the domain of behavioral regulation.

Comment 7

The finding that some caregivers mistakenly believed SMS messages incurred a cost, which reduced engagement, is important. Yet the manuscript does not report whether any efforts were made during the trial to correct this misunderstanding proactively. This represents a missed opportunity to improve intervention design.

Response: Your observation has been noted. These qualitative interviews and focus groups were conducted after the trial concluded and therefore we were unable to provide feedback during trial implementation to correct these misunderstandings. These findings can be helpful to help improve the design of other mobile SMS interventions.

Comment 8

Health workers reported spending between 30 minutes and 1.5 hours daily responding to messages, with peaks of up to 50 messages per day. The feasibility of scaling this intervention to routine health system settings, where staff already face heavy workloads, was not adequately explored. This raises concerns about sustainability outside of a research context.

Response: We agree that the additional workload associated with responding to caregiver messages is an important consideration for the feasibility and sustainability of scaling this intervention within routine health system settings. Although health care workers in our study were able to establish workflows that allowed them to read and respond to messages within their schedules and generally perceived the intervention as feasible to integrate into their workstreams, our findings also indicate that the time and staffing demands associated with providing ongoing two-way SMS support would need to be carefully considered before scale-up. We have highlighted this consideration in the Discussion, noting that appropriate staffing, task-shifting strategies, or automation of routine responses may be important for supporting the feasibility and sustainability of this approach at scale (page 27):

“providing ongoing support through two-way SMS platforms may introduce additional demands on HCWs, particularly in terms of time required to review and respond to caregiver messages. Without careful program design, including appropriate staffing, task-shifting strategies, or automation of routine responses, these added responsibilities could increase workload and potentially limit the feasibility and scalability of such interventions within already resource-constrained health systems.”

Comment 9

Social support from family members, particularly husbands and children, emerged as a strong facilitator of engagement, while resistance from mothers-in-law was a barrier. The study does not propose specific strategies to address negative social influences or to engage resistant family members in future implementations.

Response: Thank you for this timely comment. We agree that engaging mothers-in-law and fathers could improve adoption of this intervention. There are various ways to engage resistant family members within child nutrition programs. We have added additional insights based on your comment on pages 28-29:

“Evidence from maternal and child nutrition programs suggests that engaging additional family members during program implementation, including male partners and senior female relatives, could enhance caregiver involvement and overall program success [21]. Approaches such as grandmother or husband support groups, digital health interventions that engage non-primary caregiver household members, and facility-based interventions such as couples counseling sessions during routine health visits could increase involvement and engagement in child nutrition programs [21].”

Comment 10

The intervention's potential to empower caregivers and transform them into community health advocates is compelling. However, the data supporting this claim are largely anecdotal, consisting of a few illustrative quotes. No systematic attempt was made to measure the extent or impact of this community-level diffusion of knowledge and skills.

Response: This is correct, it was not within our scope to measure the extent of community-level diffusion of empowering caregivers as health advocates. Since the interviews and focus groups only involved caregivers who participated in the trial, we did not aim to measure community member perceptions of caregivers involved in the intervention. This should not undermine the finding that caregivers felt increased agency due to their participation in the Family MUAC + SMS intervention and the internal motivation they felt to measure their children, and children in their neighborhoods.

Comment 11

Phone sharing was identified as a significant barrier to uptake, yet the manuscript does not discuss whether alternative communication channels (e.g., voice calls, community health worker visits, or group messaging) were considered or tested for caregivers without private phone access. This limits the practical relevance of the findings for the most disadvantaged populations.

Response: Thank you for this insightful comment. As SMS was a central component of our intervention (along with participation in Family MUAC), we targeted women who had any access to a phone – either individually owned or a shared phone. The two-way SMS approach was being evaluated as a potentially more scalable and less resource-intensive alternative to the routine CHW visits that are part of the standard of care in Kenya. Our findings highlight phone sharing as an important barrier to equitable participation in digital health interventions. While testing alternative communication channels or providing phones was beyond the scope of this study, future research should examine strategies such as phone provision or alternative communication modalities to address this barrier and support equitable scale-up.

Comment 12

The conclusion states that the intervention is "acceptable, feasible, and appropriate" based on qualitative data from a single region in western Kenya. While these findings are valuable for hypothesis generation, the lack of quantitative generalizability data or a formal implementation outcomes framework means the conclusions should be framed more cautiously, acknowledging the context-specific nature of the results.

Response: We appreciate your comment. We applied the Theoretical Domains Framework, an implementation science framework to structure data analysis and identify themes in a systematic way. Applying an implementation science framework is a common way to rigorously assess the implementation outcomes of acceptability, feasibility, appropriateness, perceived cost of a program or intervention. On the note about the “generalizability” of the data, we have added that the findings apply to the western Kenyan context on page 25 and we noted that the transferability of our study findings to contexts with different characteristics may be limited on page 30. However, consistent with qualitative research principles, the goal is not to produce universally generalizable results, but rather to explore topics in depth and provide rich, context-specific insights that may support transferability to similar settings.

Reviewer #9: Introduction:

Page 3 Line 43: It seems that the present study is only qualitative and not a mixed methods one. No quantitative data is provided.

Response: Thank you for this insightful comment. We used quantitative cut-offs to define the sampling frame for engagement, but we do acknowledge that most data and analysis presented are qualitative. Our quantitative data analyses were minimal and served to set up the qualitative analyses. Examples of the quantitative data we analyzed can be found on page 17:

“Families in the intervention arm had high uptake of the intervention with 94% responding to one text message or more over the period of enrollment [1]. Caregivers in the intervention sent a median of 20 messages (interquartile range (IQR): 15-24) over the six-month enrollment period.”

However, due to the limited nature of our quantitative analysis compared to qualitative data and results, we have revised the study design to reflect a qualitative study and removed the study’s description as mixed methods as reflected in the Abstract on page 2,

“This qualitative study involved eight focus group discussions with caregivers enrolled in the Mama Aweza trial and fourteen in-depth interviews with caregivers who participated in the intervention arm”

Additionally, on page 3, “We employed a qualitative design to investigate the determinants of engagement with Family MUAC supported by two-way SMS intervention for purposes of development and expansion. We used qualitative data to identify and compare responses between caregivers whose children did and did not become acutely malnourished during the study.”

Methods:

How was validity and reliability of qualitative data gathered determined?

Response: Thank you for this comment. To enhance the rigor and trustworthiness of the qualitative data and analysis, all IDIs and FGDs were audio-recorded, transcribed, and translated into English. To ensure transcription quality, investigators who did not conduct the original transcriptions performed random spot checks for each transcript by comparing the audio recordings with the transcripts. Any discrepancies prompted a full review of the transcript. Additionally, two primary coders independently coded the first two transcripts using the a priori TDF-based codebook and inductively identified new codes. Emerging codes and differences in code application were discussed in consensus meetings involving additional members of the research team, and a third researcher acted as a tiebreaker when consensus could not be reached. Code definitions and inclusion and exclusion criteria were refined throughout this iterative process. Finally, the final themes and conceptual framework were discussed among the coders and research team to ensure that they reflected the patterns identified across the FGDs and IDIs.

Results:

By presenting the number of each group in the text, Table 1 can be removed:

“(1) caregivers in the intervention group whose child did not develop acute malnutrition (n=12), (2) caregivers in the intervention group whose child became acutely malnourished (n=13), (3) SOC participants whose child did not develop acute malnutrition (n=13), and (4) SOC participants whose child became acutely malnourished over the duration of the study (n=13).”

Response: Thank you, this has been modified as suggested, and Table 1 was removed.

Despite the claim of authors for using mixed method, only the results of qualitative study have been presented.

Although tables have been added to the text of qualitative findings at the suggestion of the previous reviewers, the way of presenting the findings is not appropriate and it is better to present qualitative tables containing themes and sub-themes.

Response: We have taken into consideration your feedback and revised the study design to a qualitative study. Additionally, Figure 1 depicts themes and sub-themes for this study through a schematic. Tables 2-5 included at the request of previous reviewers present additional data and constructs organized by theme. While Figure 1 is the overarching summary of themes and constructs, the additional tables provide more nuanced insights under each theme.

Discussion:

The following related article is not referenced:

Abdissa, H.G.; Duguma, G.B.; Gerbaba, M.; Noll, J.; Sori, D.A.; Koricha, Z.B. Acceptability, Feasibility, and Appropriateness of Mobile Phone Messaging-Based Message-Framing Intervention for Promoting Maternal and Newborn Care Practices. Int. J. Environ. Res. Public Health 2025, 22, 864. https://doi.org/10.3390/ijerph22060864.

Response: Thank you for this suggestion. We cited this reference to support our insights on phone sharing as a barrier in mHealth interventions.

Reviewer #10: In review of the article, the authors have taken into account the vast majority of previous peer review comments and made the requested improvements where noted in the article. In particular, the explanations in the methods section, the more detailed presentation of participant characteristics, the expansion of limitations, and the clarification of various conceptual points have increased the publishability of the study.

However, although the Discussion section has been supplemented with additional sources as mentioned in the previous revision, the improvement remains limited, and a more detailed discussion of the similarities and differences between the findings and previous studies, particularly regarding caregiver involvement, measurement accuracy, social support mechanisms, and the sustainability of the intervention, would be better.

Response: Thank you for this comment and for highlighting these gaps in our discussion. We added more to the discussion to reflect on the similarities and differences between our study findings and previous studies. In particular, differences with other studies involving caregiver involvement and social support mechanisms are now highlighted on page 28,

“Evidence from maternal and child nutrition programs suggests that engaging additional family members during program implementation, including male partners and senior female relatives, could enhance caregiver involvement and overall program success [22]. Approaches such as grandmother or husband support groups, digital health interventions that engage non-primary caregiver household members, and facility-based interventions such as couples counseling sessions during routine health visits could increase involvement and engagement in child nutrition programs [22].”

On page 28, “HCWs emphasized the need for continuous guidance to address caregiver questions and ensure accurate measurements. Prior studies have also underscored the necessity of sustained training in community-based health interventions to maintain data integrity and program effectiveness, and to improve sustainability.”

Additionally, we demonstrate similarities between our study and previous studies on page 27, “Caregivers reported increased confidence with monitoring their child’s health and became health educators within their communities, a finding supported by previous studies in South Africa and Niger using a Family MUAC approach.”

Also, in response to the previous revision, while tables regarding the number of FGD participants have been added, reporting the number of participants in each focus group interview separately would be a more transparent approach.

Response: Thank you for suggesting this clarification. We have added on page 9, “We conducted eight FGDs (51 total FGD participants with group size ranging from 6-7 participants)” to clarify the number of participants in each group.

Furthermore, the lack of sociodemographic data for participants is a significant limitation of the study. Therefore, it was not possible to assess potential differences between high and low-interaction participants in terms of age, education level, socioeconomic status, or other individual characteristics. This partially limits the interpretation and transferability of the findings.

Response: We acknowledge this as a limitation of the study. We did not collect additional demographic information from caregivers in the qualitative sample, in part to protect confidentiality. We have outlined the lack of reporting on demographic characteristics for interview participants in the discussion, on page 29:

“We did not collect detailed socio-demographic characteristics of participants, which limits our ability to assess how factors such as literacy, socioeconomic status, or access to broader household resources may have influenced engagement with the intervention. Although we purposively sampled participants across sites and levels of engagement, the absence of these data restricts our ability to examine potential differences between groups.”

Though we did collect the same number of participants from each facility to ensure all sites had equal representation.

Overall, the revisions made are satisfactory, and the remaining issues are minor improvements. Therefore, I believe the article can be published after minor revisions.

Reviewer #11: The methodology section presented in " PONE-D-25-63080_R1.docx" is highly rigorous, well-structured, and directly aligned with your study’s objectives. The choice of an explanatory sequential mixed-methods design (quan → QUAL) is highly appropriate for contextualising the quantitative trial results through the lived experiences of the caregivers.

Furthermore, your precise, metrics-driven purposive sampling strategy which uses stratifying by clinical outcomes and utilizing strict mathematical thresholds for high and low platform engagement significantly strengthens the comparative validity of your findings. The systematic application of the Theoretical Domains Framework (TDF) combined with a robust dual-coder analysis framework ensures excellent theoretical and analytical depth.

two minor comments:

1. Page 20, title: it would be better if you avoid using the acronym SMS in the title to improve readability.

Response: Thank you for this suggestion. We have considered this recommendation; however, we have retained the acronym “SMS” in the title because it is a widely recognized and commonly used term in the digital health literature. We believe that retaining “SMS” improves conciseness without compromising readability.

2. As a minor formatting note for the final layout, please ensure that the title and caption for Figure 1 are placed directly below the figure, as is customarily done in academic writing.

Response: Thank you for flagging this for editors to consider when typesetting the document.

Reviewer #12: The authors have responded comprehensively to the reviewers' comments and have addressed the majority of the concerns raised during peer review. Clarifications regarding participant eligibility, MUAC color classifications, study procedures, intervention implementation, limitations, and contextualization of the findings have substantially improved the manuscript. However, I still have two minor concerns.

First, the relationship between the previously published RCT and the current qualitative study remains somewhat unclear. I think some clarity is required in study design. I believe the manuscript is essentially a qualitative follow-up study informed by trial data. I want to know the qualitative component was planned from the outset as part of a mixed-methods design, or the qualitative study was conducted later as a separate follow-up study. Because the relationship between the current qualitative study and the previously published RCT is not immediately clear from the abstract unless a reader is familiar with the earlier publication.

The manuscript would benefit from greater clarity regarding the overall study design specially for readers not familiar with previous publication if authors can please clarify whether the study was originally conceptualized and conducted as a mixed-methods study (with both quantitative and qualitative components planned a priori) or whether the qualitative work was undertaken subsequently?

Response: Both the quantitative and quantitative components were planned a priori as outlined in the trial registration (clinicaltrials.gov: NCT03967015) and published protocol paper. This qualitative component was planned from the outset, at the design stage for the Mama Aweza trial. The research team leveraged caregivers enrolled in the parent trial to purposively sample participants and to identify and interview caregivers with high and low engagement. We confirm that the qualitative study informed by trial data was pre-planned before the Mama Aweza trial was carried out. We also revised the abstract and methods sections to reflect the qualitative design of this present study.

Second, while the authors have acknowledged the absence of socio-demographic information as a limitation, factors such as literacy, education, phone ownership, and socioeconomic status appear highly relevant to understanding differences between high and low interactors in an SMS-based intervention. Consideration of these characteristics would have strengthened interpretation of the findings and identification of barriers to engagement.

Response: Thank you for highlighting this consideration. We acknowledge this is a significant limitation of this study and have expanded the discussion to capture this constraint more fully. Although the qualitative interviews identified important differences in barriers and facilitators of engagement between high and low interactors, including challenges related to phone ownership and phone sharing, we were unable to systematically assess whether differences in characteristics such as literacy, education, socioeconomic status, and other household resources contributed to differences in engagement. We have clarified that the absence of these data limits our ability to interpret differences between engagement groups and identify potential sociodemographic barriers to participation.

We have included additions on page 30,

“Although we purposively sampled participants across sites and levels of engagement, the absence of these data restricts our ability to examine potential differences between groups. Consideration of these characteristics could provide further insights into barriers to engagement.”

I also have a suggestion regarding title and abstract: Authors should mention study design in the abstract

Abstract word count is 315. I suppose it shoudnt exceed 300? The abstract doesn’t reflect it is a mixed method clearly!

Response: We added that the study is a qualitative study in the abstract and reduced the Abstract to under 300 words.

The title may benefit from replacing Family MUAC with "family MUAC screening" in the study title like “Acceptability, feasibility, appropriateness, uptake and cost perceptions of Family Mid- Upper Arm Circumference screening supported by two-way SMS in western Kenya” to more clearly convey that the intervention involves caregiver-led screening for acute malnutrition rather than MUAC measurement alone.

Response: Thank you for this suggestion. We have retained Family MUAC in the title because it is the established term used in the literature to describe this caregiver-led approach to screening children for acute malnutrition through mid-upper arm circumference measurement. Therefore, we believe that adding “screening” to the title would be somewhat redundant. However, we agree that it is important to clearly convey the caregiver-led screening nature of the intervention and have ensured that this is clearly described in the manuscript.

One more point Please use consistent terminology throughout the manuscript (e.g., 'health workers' or 'healthcare workers') to improve clarity.

Response: We have corrected terminology to refer to healthcare workers throughout the manuscript.

Apart from these points, I believe the authors have responded satisfactorily to the reviewers' comments and the manuscript has been considerably improved.

Reviewer #13: The comparison between high and low interactors is a central component of the study. However, no socio-demographic characteristics are presented for these groups. Without such information, it is difficult to determine whether differences in engagement reflect behavioral determinants identified through the TDF or underlying socio-economic differences. Please discuss this limitation more explicitly.

Response: We acknowledge this as a limitation of the study. We did not collect additional demographic information from caregivers in the qualitative sample, in part to protect confidentiality. We have outlined the lack of reporting on demographic characteristics for interview participants in the discussion, on page 29:

“We did not collect detailed socio-demographic characteristics of participants, which limits our ability to assess how factors such as literacy, socioeconomic status, or access to broader household resources may have influenced engagement with the intervention. Although we purposively sampled participants across sites and levels of engagement, the absence of these data restricts our ability to examine potential differences between groups.”

We did however collect the same number of participants from each facility to ensure all sites had equal representation.

The statement that the World Health Organization (WHO) recommends integrating early detection and treatment of child wasting into routine primary health care services appears to be supported by a UNICEF guidance document. Please verify the citation and either provide an appropriate WHO reference or revise the text to accurately reflect the source of the recommendation.

Response: Thank you for flagging this citation. We have updated the citation to reflect the WHO document reflecting this recommendation.

The abbreviation SMS is defined differently in the manuscript. In the Abstract, SMS is expanded as “short message system,” whereas in the Introduction it is defined as “short message service.” Please ensure consistent and correct terminology throughout the manuscript.

Response: Thank you we have corrected the Abstract to reflect the correct definition of SMS, which is Short Message Service.

The manuscript would benefit from additional information regarding the qualifications and training of the research and healthcare personnel involved in the study. Although the interviewer’s educational background and experience in qualitative data collection are described, it would be helpful to clarify whether specific training was provided on the study procedures, use of the Theoretical Domains Framework, and qualitative interviewing techniques. In addition, please indicate whether the healthcare workers involved in the intervention had prior training and expertise in child nutrition, MUAC assessment, and malnutrition management, as these factors may have influenced implementation and participant experiences.

Response: We have added details about the qualitative researcher and health professionals who participate in the intervention as suggested. These additions can be found on page 6-7:

“Prior to study commencement, the RA received training on study procedures, qualitative data collection guides, and the application of the Theoretical Domains Framework,”

and page 5:

“These trained health professionals involved in the intervention held prior training and experience in child nutrition, MUAC assessment, and management of acute malnutrition.”

For readability, please avoid beginning the description of the interviewer with initials only ("MO"). Consider introducing the individual's full name and role at first mention and then using initials thereafter, if preferred.

Response: This has been updated.

The manuscript states that seven focus group discussions were conducted with healthcare workers from different professional backgrounds. However, it is unclear whether the focus groups were profession-specific or composed of mixed cadres. Please provide additional details regarding the composition of each focus group, as group structure may influence participant interaction and the nature of the data generated.

Response: Focus groups were only conducted with caregivers. No healthcare workers participated in focus group discussions. Healthcare workers were invited to participate in in-depth interviews by cadre as presented in Table 2 (pasted below). Interviews were profession specific.

IDI participants Interview location Total interviews

Migori County referral

Hospital Homa Bay County Hospital St. Joseph Mission Hospital

High SMS interactors (caregivers) 2 3 2 7

Low SMS interactors (caregivers) 2 3 2 7

Community health workers/counselors 3 2 2 7

Nurses 2 2 1 5

Study nutritionists 1 1 2

Ministry of Health staff/partners/nutritionists 2 1 1 4

TOTAL 32

Since the intervention combines caregiver-led MUAC monitoring and two-way SMS support, it is difficult to disentangle which component contributed most strongly to acceptability and uptake. This limitation should be emphasized more clearly.

Response: Thank you for this insight. We agree that the intervention combined caregiver-led Family MUAC monitoring with two-way SMS support. The objective of this study was to assess acceptability, uptake, and other perceptions of the intervention as a whole, rather than to disentangle the individual contributions of the Family MUAC and two-way SMS components. Therefore, we do not attribute the observed perceptions or levels of uptake to either component individually.

The manuscript provides valuable insights into the acceptability and implementation of the intervention. However, the discussion could more explicitly connect the identified themes to the positive outcomes observed in the parent trial. For example, which determinants of engagement (e.g., increased caregiver self-efficacy, earlier detection of wasting, improved caregiver–healthcare worker communication) are most likely to have contributed to the reported reduction in wasting? Strengthening this link would enhance the explanatory value of the qualitative findings.

Response: Thank you for this helpful comment. We agree that the qualitative findings can provide insight into potential pathways through which the intervention may have supported the positive outcomes observed in the parent trial. However, the objective and design of the present study were not intended to quantify or establish causal relationships between individual determinants of engagement and the reduction in wasting. Therefore, we cannot determine which specific determinants contributed most strongly to the trial outcome. The qualitative findings should instead be interpreted as identifying potential mechanisms and contextual factors that may help explain how caregivers engaged with the intervention, rather than establishing the contribution of individual determinants to the reduction in wasting.

Attachment Submitted filename: Response to Reviewers.docx

# Editor report: Decision Letter 2

**DOI:** 10.1371/journal.pone.0358775.r006

**Authors:** Arupendra Mozumdar

7 Sep 2026

Acceptability, feasibility, appropriateness, uptake and cost perceptions of Family Mid-Upper Arm Circumference supported by two-way SMS in western Kenya

PONE-D-25-63080R2

Dear Dr. Means,

We’re pleased to inform you that your manuscript has been judged scientifically suitable for publication and will be formally accepted for publication once it meets all outstanding technical requirements.

Within one week, you’ll receive an e-mail detailing the required amendments. When these have been addressed, you’ll receive a formal acceptance letter and your manuscript will be scheduled for publication.

An invoice will be generated when your article is formally accepted. Please note, if your institution has a publishing partnership with PLOS and your article meets the relevant criteria, all or part of your publication costs will be covered. Please make sure your user information is up-to-date by logging into Editorial Manager at Editorial Manager® and clicking the ‘Update My Information' link at the top of the page. For questions related to billing, please contact billing support.

If your institution or institutions have a press office, please notify them about your upcoming paper to help maximize its impact. If they’ll be preparing press materials, please inform our press team as soon as possible -- no later than 48 hours after receiving the formal acceptance. Your manuscript will remain under strict press embargo until 2 pm Eastern Time on the date of publication. For more information, please contact onepress@plos.org.

Kind regards,

Arupendra Mozumdar, Ph.D., M.Sc.

Academic Editor

PLOS One

Additional Editor Comments (optional):

The manuscript addresses a critical public health issue, namely acute malnutrition and its management, through an innovative approach implemented at a large scale. The paper effectively documents the implementation and optimal use of the Family MUAC approach, supported by simple digital technology, to reduce nutritional deficiencies in low-resource settings.

The manuscript has improved significantly following two rounds of revision. The authors have carefully addressed the comments raised by the reviewers and have appropriately incorporated their suggestions. I find the revised version of the manuscript to be of sufficient quality for publication and therefore recommend its acceptance.

Reviewers' comments:

# Editor report: Acceptance letter

**DOI:** 10.1371/journal.pone.0358775.r007

**Authors:** Arupendra Mozumdar

PONE-D-25-63080R2

PLOS One

Dear Dr. Means,

I'm pleased to inform you that your manuscript has been deemed suitable for publication in PLOS One. Congratulations! Your manuscript is now being handed over to our production team.

At this stage, our production department will prepare your paper for publication. This includes ensuring the following:

* All references, tables, and figures are properly cited

* All relevant supporting information is included in the manuscript submission,

* There are no issues that prevent the paper from being properly typeset

You will receive further instructions from the production team, including instructions on how to review your proof when it is ready. Please keep in mind that we are working through a large volume of accepted articles, so please give us a few days to review your paper and let you know the next and final steps.

Lastly, if your institution or institutions have a press office, please let them know about your upcoming paper now to help maximize its impact. If they'll be preparing press materials, please inform our press team within the next 48 hours. Your manuscript will remain under strict press embargo until 2 pm Eastern Time on the date of publication. For more information, please contact onepress@plos.org.

You will receive an invoice from PLOS for your publication fee after your manuscript has reached the completed accept phase. If you receive an email requesting payment before acceptance or for any other service, this may be a phishing scheme. Learn how to identify phishing emails and protect your accounts at https://explore.plos.org/phishing.

If we can help with anything else, please email us at customercare@plos.org.

Thank you for submitting your work to PLOS One and supporting open access.

Kind regards,

PLOS One Editorial Office Staff

on behalf of

Dr. Arupendra Mozumdar

Academic Editor

PLOS One
